# A QTL Study for Regions Contributing to *Arabidopsis thaliana* Root Skewing on Tilted Surfaces

**DOI:** 10.1534/g3.111.000331

**Published:** 2011-07-01

**Authors:** Laura M. Vaughn, Patrick H. Masson

**Affiliations:** Laboratory of Genetics, University of Wisconsin–Madison, Madison, Wisconsin 53706

**Keywords:** *Arabidopsis*, root, skewing, waving, *cis*-prenyltransferase

## Abstract

Plant root systems must grow in a manner that is dictated by endogenous genetic pathways, yet sensitive to environmental input. This allows them to provide the plant with water and nutrients while navigating a heterogeneous soil environment filled with obstacles, toxins, and pests. Gravity and touch, which constitute important cues for roots growing in soil, have been shown to modulate root architecture by altering growth patterns. This is illustrated by *Arabidopsis thaliana* roots growing on tilted hard agar surfaces. Under these conditions, the roots are exposed to both gravity and touch stimulation. Consequently, they tend to skew their growth away from the vertical and wave along the surface. This complex growth behavior is believed to help roots avoid obstacles in nature. Interestingly, *A. thaliana* accessions display distinct growth patterns under these conditions, suggesting the possibility of using this variation as a tool to identify the molecular mechanisms that modulate root behavior in response to their mechanical environment. We have used the Cvi/L*er* recombinant inbred line population to identify quantitative trait loci that contribute to root skewing on tilted hard agar surfaces. A combination of fine mapping for one of these QTL and microarray analysis of expression differences between Cvi and L*er* root tips identifies a region on chromosome 2 as contributing to root skewing on tilted surfaces, potentially by modulating cell wall composition.

Healthy roots take on an architecture that securely anchors the plant in soil and allows for dynamic growth toward adequate water and nutrients. The heterogeneous nature of soil leads to gradients in elements that the plant may need to acquire or avoid, and obstacles such as rocks must be bypassed to continue a downward trajectory. These environmental inputs combine with intrinsic developmental cues to determine root growth behavior. Understanding the genetics behind primary root elongation and how it is affected by various environmental stimuli may help in the breeding of crops better suited to their local soil conditions.

A common way to observe root growth behavior is to germinate seedlings on a hard agar surface in a petri dish. The root is more easily observed than it would be in a soil environment, and the hard surface provides an obstacle as the roots grow along it, similar to that which a rock may present to a root in its natural environment. Also, if the plate is tilted back, the root tip will attempt to grow downward along the gravity vector. However, the hard medium is impenetrable, therefore, gravitropism will press the root tip against the agar causing stronger touch stimulus. The combined effect of gravity and touch stimuli on the root tip, along with other surface-derived stimuli and intrinsic cell machinery, cause characteristic root growth behaviors under these conditions (Okada and Shimura 1990; Rutherford and Masson 1996, Simmons *et al.* 1996).

One such behavior is root skewing or slanting, whereby roots tend to deviate their growth progressively away from the gravity vector, always in the same direction (typically rightward, when viewed from the back of the plate, through the agar). Rightward skewing is often accompanied by a left-handed rotation of the root tip about its axis, visible as a left-handed rotation of the epidermal cell files. The degree of skewing varies in wild-type roots of *Arabidopsis thaliana*, with some accessions showing little or none and others displaying a distinct rightward skew on tilted surfaces (Simmons *et al.*1995; Rutherford and Masson 1996). Mutants have been discovered that have enhanced right skew or a pronounced leftward skew, the latter of which has never been seen in a wild type *A. thaliana* accession. Often, these skewing mutants display epidermal cell file rotations (CFR), the degree of which correlates with the level of skewing. The handedness of the CFR also correlates with the direction of skewing: left- handed CFR for rightward skewing and right-handed CFR for leftward skewing (Rutherford and Masson 1996; Furutani *et al.* 2000; Whittington *et al.* 2001; Sedbrook *et al.* 2002; Thitamadee *et al.* 2002; Yuen *et al.* 2003; Ishida *et al.* 2007; Korolev *et al.* 2007; Wang *et al.* 2007; Bisgrove *et al.* 2008; Nakamura and Hashimoto 2009). However, this correlation between CFR, and level of skewing is not observed in all mutants. For instance, the *spr2* mutant has a strong helical growth phenotype, yet its epidermal cell file rotation remains wild-type (Furutani *et al.* 2000).

Many skewing mutants also have abnormal cortical microtubule arrays. In wild-type *A. thaliana* interphase cells in the epidermal and lateral cap layers of the root elongation zone, cortical microtubules are arranged mostly in a transverse orientation. This arrangement correlates with the cells in this region expanding anisotropically, in a direction perpendicular to the transverse arrays (reviewed in Harrison *et al.* 2008). When the cells approach the shootward portion of the elongation zone (as defined in Baskin *et al.* 2010), the cortical arrays will briefly shift to oblique right-handed helical arrangements. A left-handed cell file rotation is often visible at this region of the root, corresponding to the right-handed arrays (Okada and Shimura, 1990; Rutherford and Masson, 1996). As the cells continue to the maturation zone, the arrays shift again, this time to form a mostly longitudinal network. Finally, the microtubules become disorganized as the cell stops elongating (Sugimoto *et al.* 2000).

Several skewing mutants have cortical microtubule arrays that fall into helical patterns before reaching the basal side of the elongation zone, the handedness of which correlates to the handedness of the skewing. As with CFR correlations, not all skewing mutants have altered cortical microtubule arrays, including *spr2* as well as *eb1* and *spr1* (Furutani *et al.* 2000; Sedbrook *et al.* 2004; Bisgrove *et al.* 2008). Furthermore, altered arrays in root slanting mutants are not always found in the form of helical patterns. In some cases, arrays are simply disorganized (Bichet *et al.* 2001; Whittington *et al.* 2001; Burk and Ye 2002; Webb *et al.* 2002; Bouquin *et al.*, 2003).

Most skewing mutants found to date have changes in tubulin genes or in genes that produce microtubule-associated proteins. These studies have shown that changes in the structure of α-, β-, and γ- tubulin subunits can affect microtubule assembly and cause helical root growth behavior, often accompanied by abnormal helical microtubule arrays (Thitamadee *et al.* 2002; Pastuglia *et al.* 2006; Ishida *et al.* 2007; Buschmann *et al.* 2009). Also, changes in proteins that regulate microtubule polymerization, bundling, and severing can alter the organization and dynamic instability of microtubules and lead to changes in root skewing (reviewed in Sedbrook and Kaloriti 2008; Wasteneys and Ambrose 2009). A few skewing mutants have been discovered that do not directly affect microtubules, but instead are predicted to have an impact on cell wall composition or signaling cascades that contribute to proper anisotropic cell expansion during root growth (Sedbrook *et al.* 2002; Hu *et al.* 2003; Yuen *et al.* 2005).

In addition to skewing, roots may exhibit waving behavior when grown on hard surfaces. This behavior is common to many plants exposed to tilted hard surfaces and has been studied by several scientists over the years, including Charles Darwin (reviewed in Vaughn *et al.* 2010; Darwin and Darwin 1880). In this complex manner of growth, the root tip changes direction at regular intervals as it elongates on the surface, producing a sinusoidal wave pattern along the root’s length. The waving pattern may include alternating left- and right-handed epidermal cell file rotation, at least under some conditions (Buer *et al.* 2003). Several models have been proposed to explain wavy growth, but in general it is agreed to be a consequence of gravitropism, thigmotropism, intrinsic cellular programs, and possibly other stimuli. The behavior may be important for roots trying to navigate through various soil microenvironments (Okada and Shimura 1990; Simmons *et al.*1995; Rutherford and Masson 1996; Migliaccio and Piconese 2001; Buer *et al.* 2003; Thompson and Holbrook 2004). As with skewing, mutants have been identified that display altered waving behavior. Studies of these mutants have highlighted the importance of signaling by the hormones auxin and ethylene as well as other molecules like small peptides in regulating the behavior (reviewed in Migliaccio and Piconese 2001; Oliva and Dunand, 2007).

While mutants have been helpful in elucidating the genetics behind the root growth behaviors on hard surfaces, our knowledge of the mechanisms underlying these complex growth responses remain rudimentary. Another way to gain new insights into root growth patterns is to use the natural variation that exists among members of plant species. Variation among the *A. thaliana* accessions Columbia, Landsberg *erecta*, and Wassilewskija for skewing has already been noted (Simmons *et al.* 1996). Also, we know that defined genetic backgrounds modify waving because in at least one case the same mutant allele (*spr1/sku6*) results in distinct phenotypes when introgressed into different genetic backgrounds (Sedbrook *et al.* 2004).

In this study, we used a recombinant inbred line (RIL) population created from the *A. thaliana* accessions Cape Verde Islands (Cvi) and Landsberg *erecta* (L*er*) (Alonso-Blanco *et al.* 1998) for quantitative trait locus (QTL) mapping of root growth behaviors on agar surfaces. Our results suggest that a region on chromosome 2 is contributing the most to the difference in skewing between these accessions, although other loci are also implicated. No loci in the candidate region have previously been associated with root skewing. We also performed a microarray analysis using Cvi and L*er* root tips from seedlings grown on tilted hard agar to identify genes with differing expression between these accessions in genomic locations that overlap with the QTL, leading to interesting candidate loci between 9.3 and 11.2 Mb on chromosome 2.

## Materials and Methods

### Plant materials and soil growth conditions

**Figure 1:**
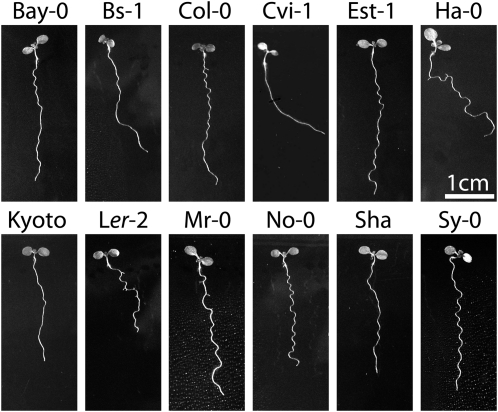
Natural variation in *Arabidopsis thaliana* root growth behaviors. Representative seedlings from a variety of *A. thaliana* accessions that were grown on tilted hard agar plates. View is from the back of the plate, through the agar.

QTL analysis was carried out with the following stocks from the Arabidopsis Biological Resource Center (ABRC, Columbus, OH): Cvi-1 (CS8580), L*er*-2 (CS8581), and Cvi/L*er* recombinant inbred lines (RILs) (CS22000). This population consists of 162 inbred lines that were genotyped at 293 marker loci (Alonso-Blanco *et al.* 1998). Ten plants per RIL plus multiple Cvi and L*er* plants were propagated side by side in a soil composed of Jiffy mix and vermiculite (4:1 volumetric ratio), using a 22°C growth room with a 16/8-hr light/dark cycle, to bulk seeds for analysis. Light was provided by cool-white fluorescent bulbs at a fluence rate of 35-50 µmol⋅m^−2^⋅s^−1^. Seed stocks used in fine mapping include near isogenic lines (NILs) HGI2.1, 0.2, 0.3, 0.4, and 0.5 provided by Julin Maloof (University of California–Davis, Davis, CA; derived from the LIGHT2 NIL in Borevitz *et al.* 2002). The DOG NILs, first used in studies of delay of germination (hence DOG), were provided by Leonie Bentsink (Wageningen University, Wageningen, Netherlands; Alonso-Blanco *et al.* 2003). The NILs LCN2-4, -6, -7, -8, and -9 and LCN4-6 and -7 (Keurentjes *et al.* 2007) were obtained from the Nottingham Arabidopsis Stock Center (Nottingham, UK; N17079, N17081, N17082, N17083, N17084, N17115, and N17116). The seeds for Figure 1 were obtained from Christopher Schwartz (University of Wisconsin–Madison, Madison, WI; Sy-0) and the ABRC (Bay-0/CS57923, Bs-1/CS6627, Col/CS60000, Est-1/CS1151, Ha-0/CS1218, Kyoto/CS3964, Mr-0/CS1373, No-O/CS1394, Sha/CS57924). For all experiments, the genotypes to be compared were planted in the growth room at the same time and harvested on or near the same day.

### The wave assay

A 1/2 strength LSPS medium (LSP04-1LT; Caisson Labs, North Logan, UT) was supplemented with 1.5% agar type E (Sigma-Aldrich, St. Louis, MO) and poured into square petri dishes to form a hard medium mostly impenetrable to *A. thaliana* roots. Seeds were surface sterilized with 20% bleach + 0.01% SDS solution then rinsed four times with water. The seeds were sewn on the surface of the medium, and plates were sealed with micropore tape (3M; St. Paul, MN; #1530-0). Seeds were stratified on the plates for 4-6 days at 4°C, wrapped in two layers of aluminum foil, to overcome the dormancy phenotype of Cvi. Plates were then placed in a vertical orientation in either a Conviron TC16 growth chamber (Conviron, Winnipeg, Manitoba, Canada) for QTL trial 1, or in an Enconair AC60 growth chamber (Enconair, Winnipeg, Manitoba, Canada) for all other trials. The temperature was set at 22°C in both chambers. Light was provided by cool white fluorescent tubes at a fluence rate of 45-50 µmol⋅m^−2^⋅s^−1^ in the Conviron chamber (QTL Trial 1), and 80-125 µmol⋅m^−2^⋅s−^1^ in the Enconair chamber (all other assays), following a 16/8-hr light/dark cycle. Seedlings were grown under these conditions for 3 days. Then, the locations of the root tips of all germinated seedlings were marked with a black felt-tip marker on the back of the plate, and the plates were tilted back at a 30° angle. After 2 days of growth at the 30° tilt, images were taken of the roots through the medium from the back of the plates with a digital camera.

### Measurements of the roots

**Figure 2:**
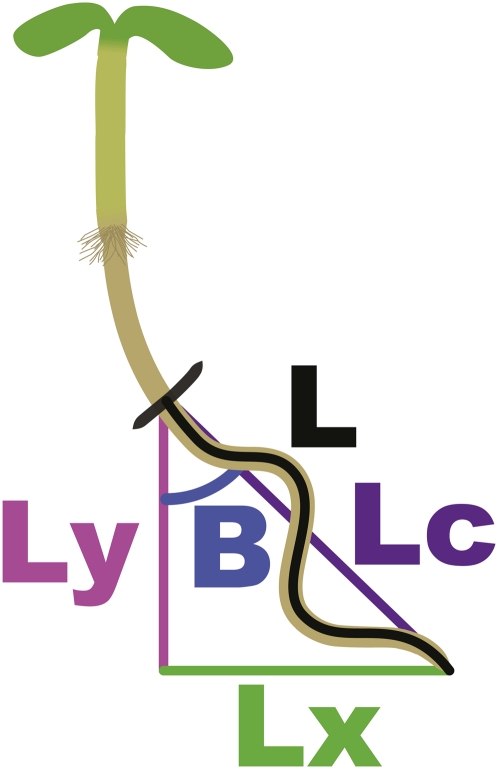
Measurements taken of seedlings (after Grabov *et al.* 2005). Measurements were started at the black felt-tip marker line indicating the position of the root tip on the back of the plate after 3 days of vertical growth. After 2 more days of growth at a 30° tilt and image capture, a line was traced along each root from the black mark to the tip to get primary root length (L). Then, a line was drawn directly from the black mark to the root tip to get the chord of growth, Lc. Dividing Lc by L gives an indication of the root’s straightness along its growth trajectory. Angle B is calculated as the angle of root tip’s displacement from the vertical, with the vertical being 0° and increasing to 90° as you move counterclockwise to the right. The trigonometric relationships between Lc and angle B allow the calculation of the Ly and Lx portions of the right triangle. Ly in a ratio with L gives the quantity known as vertical growth index (VGI), and similarly Lx divided by L gives horizontal growth index (HGI), an indication of root skewing.

NIH *Image* (http://rsb.info.nih.gov/nih-image/) version 1.62 was used to determine the following parameters as diagrammed in Figure 2: L, Ly, Lx, Lc, and the angle B. These measurements are used to compute the primary root’s length (L), vertical growth index (VGI), horizontal growth index (HGI), angle of displacement of the root tip (B), and root straightness (Lc/L) (Grabov *et al.* 2005). Length is measured by tracing the root with the “segmented line” tool from the black felt-tip markerline along the root to the root tip. VGI is the ratio of the displacement of the root tip along the y axis, Ly, divided by L. This parameter takes into account the total length of the root and modifies it based on its vertical displacement. A root whose growth depended solely on gravity but was impeded by the agar would be expected to grow straight down along the tilted agar. VGI is part of a description of the deviation of the root from this gravitropic null hypothesis of growth. HGI is the ratio of the displacement of the root tip along the x axis, Lx, divided by L. HGI specifically addresses the amount of skewing (horizontal displacement) undergone by the root. A measurement of the arccosine of angle of overall root tip displacement, B, is taken by dividing Ly by Lc. Straightness is measured by the ratio of the chord that forms a straight line between the black mark at the start of the measurement to the root tip, Lc, divided by L. The more a root is waving and deviating from the path of Lc, the lower the straightness value. However, it should be noted that a root growth path that is meandering away and then returning to Lc will have a lower straightness ratio even if it does not have a regular waving pattern. Together, these measurements give a quantifiable way to measure root morphology on the agar surface (Grabov *et al.* 2005). Calculations of the means for each of these traits were done using Microsoft Excel. Analysis of variance of the RILs was used to quantify and compare variance between and within RILs. Broad sense heritability (H^2^) was calculated by dividing the latter by the former [(variance within RILs)/(variance between RILs)].

### QTL mapping

The seed plating for the three QTL trials varied between trial 1 and trials 2/3. For QTL trial 1, each RIL was represented by one plate that contained three rows of five seeds each of the particular RIL, flanked with two Cvi seeds on one side and two L*er* seeds on the other. The plates were positioned in the Conviron TC16 chamber and exposed to the wave-assay conditions described above. For QTL trials 2 and 3, nine seeds of the particular RIL or parental control line were placed in one row near the top of the plate. These trials were performed in the Enconair AC60 chamber, also as described above. Plate arrangement was randomized in the growth chamber. For all trials, photographs were taken on day 5, and the roots were measured with NIH *Image* version 1.62. It should be noted that only the top two rows of seedlings were used for QTL analysis in trial 1 as the growth parameters of Cvi and L*er* were found to be significantly different for the top and middle rows *vs.* the bottom row (*P* < 0.01), possibly due to lower light intensity on the bottoms of the plate. Means for each trait along with genotypic data (Alonso-Blanco *et al.* 1998) were entered for QTL analysis into WinQTL Cartographer version 2.5 (http://statgen.ncsu.edu/qtlcart/WQTLCart.htm; Wang *et al.* 2010) and the R/qtl package (Broman *et al.* 2003) in R (http://www.r-project.org). For WinQTL Cartographer, maps were created using the Kosambi map function (Kosambi 1944). During composite interval mapping, the model incorporated a 0.5 step rate with a 1.0-cM window size and 10 control markers. LOD thresholds were determined by 1000 permutations (Doerge and Churchill 1996) for each of the traits. In R/qtl, the function “scanone” was used for interval mapping, and “scantwo” was used to generate the two-dimensional plots. Significance levels for each trait at the additive and epistatic levels were determined by 10,000 permutations implementing the EM algorithm for HGI and straightness, and 1000 permutations for VGI, angle B, and length. The “scantwo” output was plotted such that the additive model for a trait is below the diagonal, and the epistatic or interaction model is above it.

### Heterozygotes, NILs, and fine mapping

Seed plating for the phenotyping of NILs, various heterozygotes, and the mapping population was done in the same manner as QTL trials 2 and 3. All statistical tests were performed in Microsoft Excel. Primer design for fine mapping on chromosome 2 (supporting information, Table S4) was based on multiple sources including sequences provided by Julin Maloof (University of California–Davis), polymorphisms listed on TAIR (http://www.arabidopsis.org), polymorphisms described by Monsanto/CEREON between Columbia and Landsberg *erecta* (Jander *et al.* 2002), and polymorphic region predictions on POLYMORPH among the accessions Columbia, Burren, and Tsu (http://polymorph.weigelworld.org/; Clark *et al.* 2007; Zeller *et al.* 2008).

### Tissue collection and RNA isolation for microarray-based expression profiling

Three biological repeats with two processing replicates each were plated for the wave assay for the following lines: Cvi, L*er*, and HGI2.1. Each replicate consisted of two rows of 60 seeds per row over five plates for a total of ∼600 seedlings per replicate. The seeds on the plates were stratified for 8 days, then placed in a 22°C, 16/8-hr light/dark growth chamber for our standard wave assay. On the fifth day, approximately 7 mm of root tip was harvested for each seedling and pooled for each replicate. The order of sample collection was randomized. The root tips were flash frozen in liquid nitrogen then stored at −80°C until the time of RNA extraction. Frozen tissue was ground using a shaker with 2 ml eppendorf tube holder attachments and one 5-mm stainless steel bead (Qiagen, Valencia, CA) per tube, shaken for 30 sec at 20 beats per second. RNA extraction of the ground tissue was carried out with the RNAeasy kit (Qiagen). RNA yields were 19–32.5 μg, quantified on a Nanodrop spectrophotometer (ThermoScientific, Wilmington, DE).

### Microarray hybridization

One of the above technical replicates, consisting of three biological repeats each of Cvi, L*er*, and HGI2.1, was used for microarray hybridization. The hybridization was carried out by the Gene Expression facility at the University of Wisconsin–Madison. RNA integrity was verified with the Agilent 2100 BioAnalyzer (Agilent, Santa Clara, CA). Biotin-labeled cDNA was generated using the MessageAmp II kit (Ambion, Austin, TX). For each sample, the labeled cDNA was hybridized overnight on an ATH1 GeneChip (Affymetrix, Santa Clara, CA) in an AFX HybOven 480 (Affymetrix). Post processing was done on an automated Fluidics 450 Station (Affymetrix), and the chips were scanned with the GC 3000 G7 scanner (Affymetrix). The scanned images were preprocessed with the AFX Expression Console software. Primary data are publicly available at Gene Expression Omnibus (NCBI; accession number GSE28275).

### Microarray analysis

Quality of microarray hybridization was determined using the Affymetrix MAS5 algorithm. Expression differences were analyzed using ArrayStar 3 (DNAStar, Madison, WI). The data were preprocessed using RMA and a quantile normalization method. For statistical comparisons of expression levels, the Student’s *t*-test option was employed with a FDR (Benjamini-Hochberg correction) multiple testing correction algorithm. From the output, expression differences between the samples being compared were requested at 90%, 95%, or 99% confidence levels.

## Results

### *A. thaliana* accessions display diverse and specific growth behaviors on hard surfaces

When grown on tilted hard agar during the wave assay (see *Materials and Methods*), different *A. thaliana* accessions show a variety of root skewing and waving phenotypes (Vaughn *et al.* 2010; Figure 1). The accessions Cape Verde Islands (Cvi) and Landsberg *erecta* (L*er*) were chosen for the QTL mapping study because of their distinctive root morphologies on the wave assay, and because a genotyped RIL population was available for them at the time we initiated these experiments. Cvi has a strong skew to the right (viewed from the back of the plate) and no structured pattern of waving, whereas L*er* has a weaker right skew and a more regular pattern of waving.

Although the skewing and waving differences between Cvi and L*er* are qualitatively obvious, we needed to be able to reliably quantify the traits for the QTL study. For skewing, we used both the angle of deviation of the root tip from the vertical and a ratio known as horizontal growth index (HGI) (Grabov *et al.* 2005; Figure 2*; Materials and Methods*). The greater the angle or the greater the HGI, the greater the skew of the root to the right. We found these two measurements to be highly correlated (R^2^ = 0.93), and in this study we will predominantly use HGI as the indicator of root skew. Along with length and HGI, we also calculated vertical growth index (VGI) and the straightness of the root along the chord of growth (Lc/L) (Figure 2; *Materials and Methods*). Straightness is a rough measure of root waving since the more a root waves, the less direct its path along the axis of growth, Lc, which lowers the Lc/L ratio.

**Figure 3:**
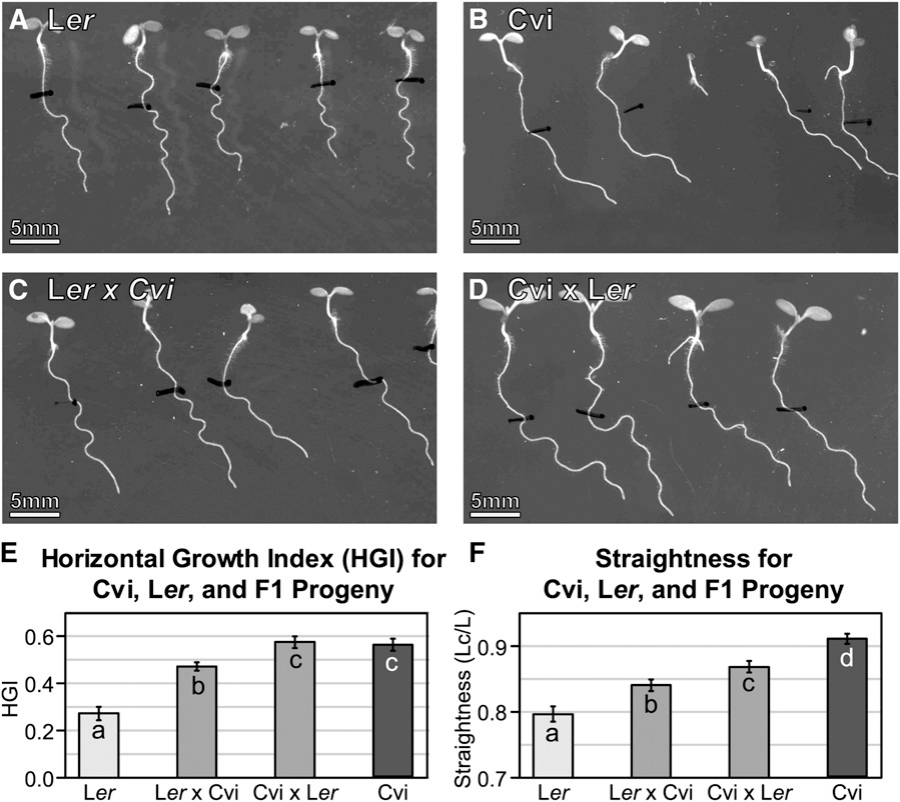
HGI and straightness phenotypes for L*er*, Cvi, and F1 progeny of crosses between Cvi and L*er*. Representative roots for each genotype grown on the wave assay are shown in panels A–D. The means for each genotype for horizontal growth index (HGI) and straightness (Lc/L) are shown in panels E and F. Statistically significant differences between genotypes are represented by distinct letter symbols in the corresponding graph bars (*P* < 0.05, pairwise *t*-test). Bars are ± SE. 41 to 77 seedlings of each type were measured. White bar = 5 mm.

The differences between Cvi and Ler for HGI and straightness on the wave assay are highly significant (Figure 3, A, B, E, and F). Cvi roots skew more strongly than L*er* as indicated by HGI, and they grow straighter along their paths as indicated by a higher Lc/L value. To get an idea of the dominance of the alleles contributing to skewing and waving in Cvi and L*er*, crosses were made to create heterozygotes. For skewing, the F1 seedlings from L*er* × Cvi display a mean root HGI that is intermediate to both parent lines and significantly different from both (Figure 3, C and E). The reciprocal cross of Cvi × L*er* produces seedlings with a mean HGI that is significantly greater than both L*er* and the L*er* × Cvi F1’s, but statistically similar to the Cvi parent (Figure 3, D and E). This indicates that maternal effects may contribute to some of the large skew diplayed by Cvi roots on hard surfaces. For straightness, both F1 crosses result in an Lc/L ratio that is intermediate to and significantly different from both parents (Figure 3F). The Cvi × L*er* cross produces a Lc/L ratio that is higher than the L*er* × Cvi F1’s at a *P* = 0.02 level, so maternal effects may be contributing to this trait as well.

### QTL analysis of root length and root growth behaviors in the Cvi/L*er* RIL population

**Figure 4:**
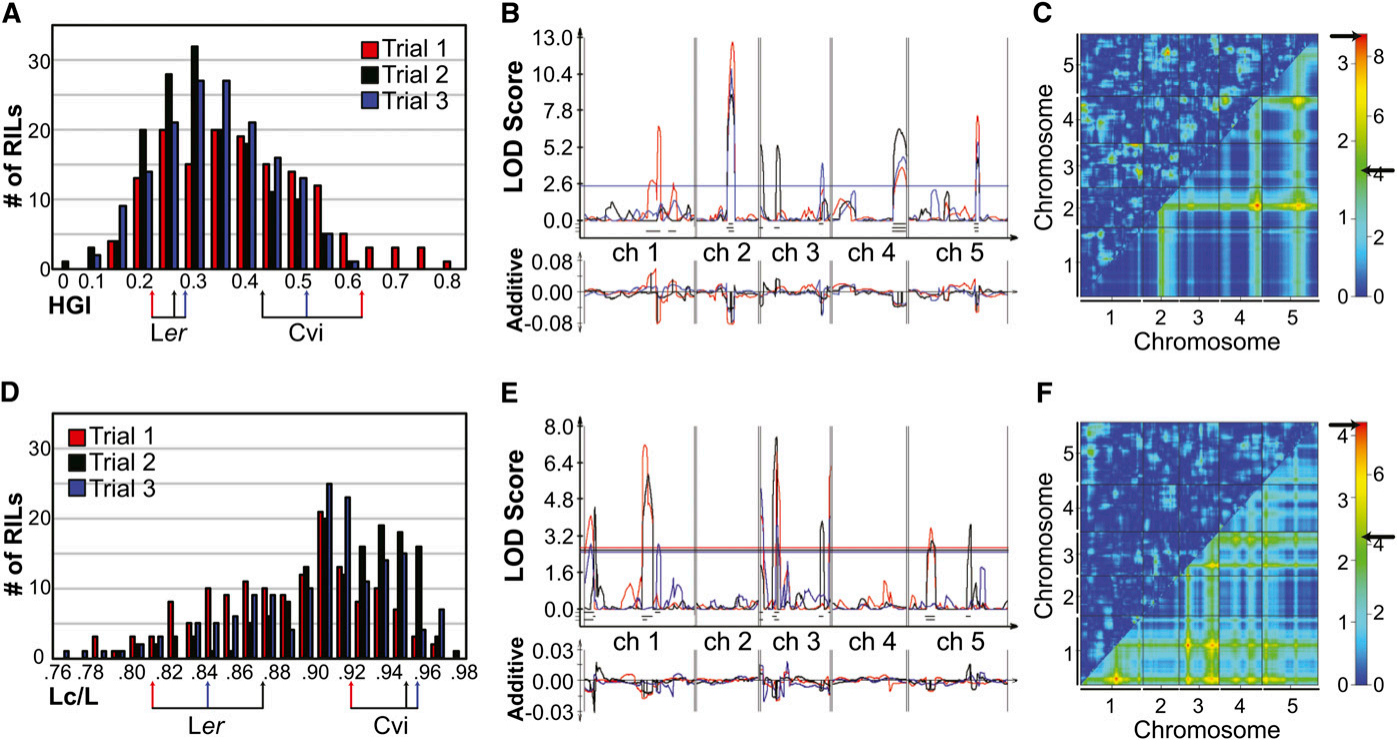
QTL analysis of skewing and straightness in the Cvi/L*er* RIL population. (A, D) Histograms of RIL means over the three QTL trials for HGI and Lc/L, respectively. The color-coded arrows indicate the means for the Cvi and L*er* parents for each trial; (B, E) Composite interval mapping (CIM) analysis over three trials for HGI (B) and Lc/L (E). The top portion of the graph gives the LOD score across the five chromosomes of *A. thaliana*. LOD significance thresholds determined by 1000 permutations for each trial are shown as horizontal lines across the graphs. 2-LOD intervals for significant QTL peaks are black bars beneath the peaks. The bottom graph is an indication of the additive value toward the phenotype of each genomic region with respect to the L*er* allele. In panels A, B, D, and E, trial 1 is represented in red, trial 2 in black, and trial 3 in blue. (C, F) Two-dimensional QTL scans for HGI (C) and Lc/L (F). Plots are for trial 2. The x- and y-axes represent positions along the five *A. thaliana* chromosomes. The region of the plot below the diagonal gives the additive QTL model, while the region above the diagonal shows epistatic interaction analysis. Heat map LOD significance thresholds were determined by 10000 permutations. The black arrow on the left LOD scale is the significance threshold for the epistatic portion of the plot, and the arrow on the right side is the threshold for the additive part.

Differences between Cvi and L*er* for various root growth behavior parameters, particularly those describing skew and straightness, made them good candidates for a QTL study on these traits. The Cvi/L*er* recombinant inbred line (RIL) population was subjected to the wave assay for three trials. Of the available 162 RILs, 149, 153, and 156 individuals were used in trials 1, 2, and 3, respectively, due to poor germination of some of the lines. Measurements made on the seedling roots include root length, HGI, VGI, angle B, and straightness. For each of these phenotypes, RIL means showed transgression with respect to parental means, and broad sense heritabilities ranged from 0.29 to 0.62 (Figure 4, A and D; Figure S1; Table S1). Both of these factors indicate the population is well-suited to QTL mapping for these traits.

The phenotype information we compiled from these measurements was paired with the genotype information already generated by Alonso-Blanco *et al.* (1998) to create QTL maps. Using composite interval mapping, significant QTL peaks were found for all traits measured (Figure 4, B and E; Figure S1, Table S2). Many of the QTL were consistently significant in all three trials. Two-dimensional (2D) scans were also performed on the traits for all three trials. As a whole, the 2D scans were relatively consistent from trial to trial for the additive portion, and consistent with the composite interval maps. The epistatic interaction portions of the graphs were more variable among the three trials. 2D scans for trial 2 for all the traits are presented here (Figure 4, C and F; Figure S1) as well as the 2D scans for trials 1 and 3 (Figure S2 and Figure S3, respectively).

Because the maternal effects may be contributing to the values of some of the traits as seen in the F1 crosses of Cvi and L*er*, we also evaluated the means of the traits for each of the three trials with respect to RIL cytoplasm donor. 117 of the RILs of the Cvi/L*er* population were created using Cvi as the maternal parent, and 45 RILs have L*er* as the maternal parent (Alonso-Blanco *et al.* 1998). None of the traits are significantly different with respect to cytoplasm for trials 1 and 2. However, in trial 3, straightness, length, and vertical growth index all showed significant differences with respect to cytoplasm donor (Figure S4). It is surprising that HGI showed no significant differences with respect to cytoplasm in the RILs considering Cvi and L*er* reciprocal heterozygotes had shown a possible maternal effect for this trait (Figure 3).

### Fine mapping of HGI QTL

We decided to focus on fine mapping the QTL for HGI due to our interest in skewing and the insights mutant studies have given us into the involvement of microtubule dynamics and cell wall composition, among others, in modulating this behavior. For HGI, all three trials predicted significant QTL in the same positions on chromosomes 2, 4, and 5 (Figure 4B). In particular, the locus centered at 40 cM on chromosome 2 was a good candidate for fine mapping due to its reproducibility over all three trials, a relatively large predicted phenotypic effect accounting for 16%–20% of the phenotypic difference between Cvi and L*er* (Table S2), and the fact that no known tubulin or microtubule-associated proteins resided in the predicted 2-LOD interval. The additive effects map shown at the bottom of the QTL-map graph (Figure 4B) indicates that having a L*er* allele in this region lowers HGI value (*i.e.*, lessens skew).

A series of NILs was used to analyze the effects of Cvi segments on skewing in the three HGI QTL regions on chromosomes 2, 4, and 5. The NILs have Cvi segments of variable lengths introgressed into the area of interest in an otherwise L*er* background. For the QTL at the end of chromosome 4, LCN4-6 is weakly significant for greater skew than L*er* (*P* = 0.05) as would be predicted if its Cvi introgression overlaps with the causative locus. However, LCN4-7 (Keurentjes *et al.* 2007) did not show a skew different from that of L*er* (Figure S5). This result may narrow the candidate interval for this QTL. However, it will be difficult to further map the causative locus because its effect on skewing is barely above significance threshold.

For the QTL centered around position 78-80 on chromosome 5, some the DOG17 NILs (Alonso-Blanco *et al.* 2003) were found to be significantly different from L*er* for HGI and several of the other measured traits (Table S3). This would be predicted as all of the traits measured over three trials had significant QTL peaks for this region of chromosome 5 (Figure 4, B and E; Figure S1), and for some epistatic interactions are predicted to affect some of these traits (Figure 4C; Figure S2, B, D, and E; Figure S3, B and E). This region seems to be a very complex contributor to root growth behavior, and further dissection is needed to narrow the regions contributing to the phenotypes and their relative additive and interactive effects.

**Figure 5:**
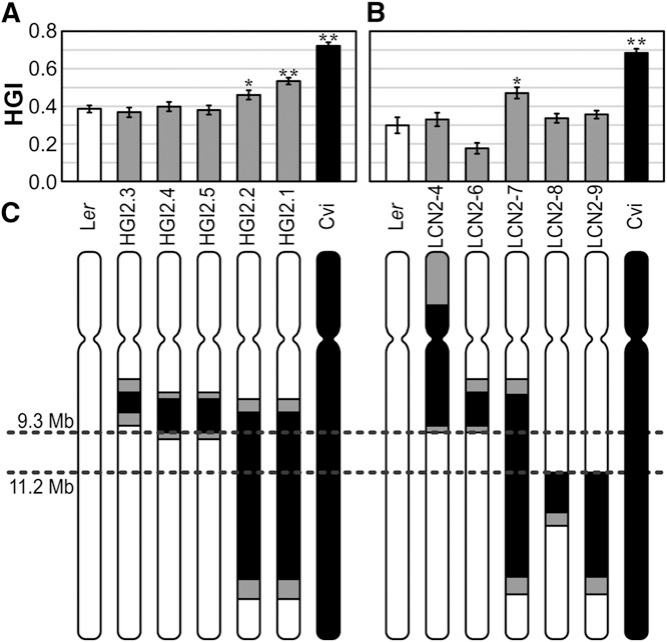
Skewing phenotypes of chromosome 2 NILs and fine mapping. (A, B) HGI means for two sets of chromosome 2 NILs. At least 30 seedlings per genotype were analyzed. ^*^Significantly different skew from L*er* (*P* < 0.01); ^**^highly significant (*P* < 0.0001). Bars are ± SE. (C) Schematic of the fine mapping of the NILs for chromosome 2. White bars are confirmed L*er* chromosomal regions, black are Cvi, and gray is where the breakpoint is undetermined. The parallel dashed lines across the chromosomes flank the probable region of a QTL for skewing.

For the chromosome 2 HGI QTL, NILs HGI2.1, 2.2, 2.3, 2.4, and 2.5 (derived from LIGHT2 in Borevitz *et al.* 2002) and LCN lines 2-4, 2-6, 2-7, 2-8, and 2-9 (Keurentjes *et al.* 2007) were analyzed. All lines have Cvi chromosome 2 segments of various lengths introgressed into an otherwise L*er* background (Figure 5C). The mean HGI values of HGI2.1, HGI2.2, and LCN2-7 were significantly greater than L*er* over several trials (Figure 5, A and B). These three lines therefore contain a segment of Cvi able to significantly increase skew over L*er* values, as predicted by the additive effect chart (Figure 4B). However, the three NILs have HGI values significantly lower than Cvi, indicating other regions of the genome are factors in Cvi’s large skewing value, including some of the QTL identified in this study. In two out of five trials with the LCN lines, LCN2-9’s skew was significantly greater than L*er* (*P* = 0.05 and *P* = 0.02; data not shown).

We developed insertion-deletion and CAPs primers identifying polymorphisms between Cvi and L*er* on Chromosome 2 (Table S4 and Table S5) and used them to define the breakpoints between introgressed Cvi and L*er* segments in these NILs. The combination of this genotypic data with the phenotypic observations for HGI of the NILs indicate the causative QTL is located between 9.3 and 11.2 Mb (schematic in Figure 5C).

**Figure 6:**
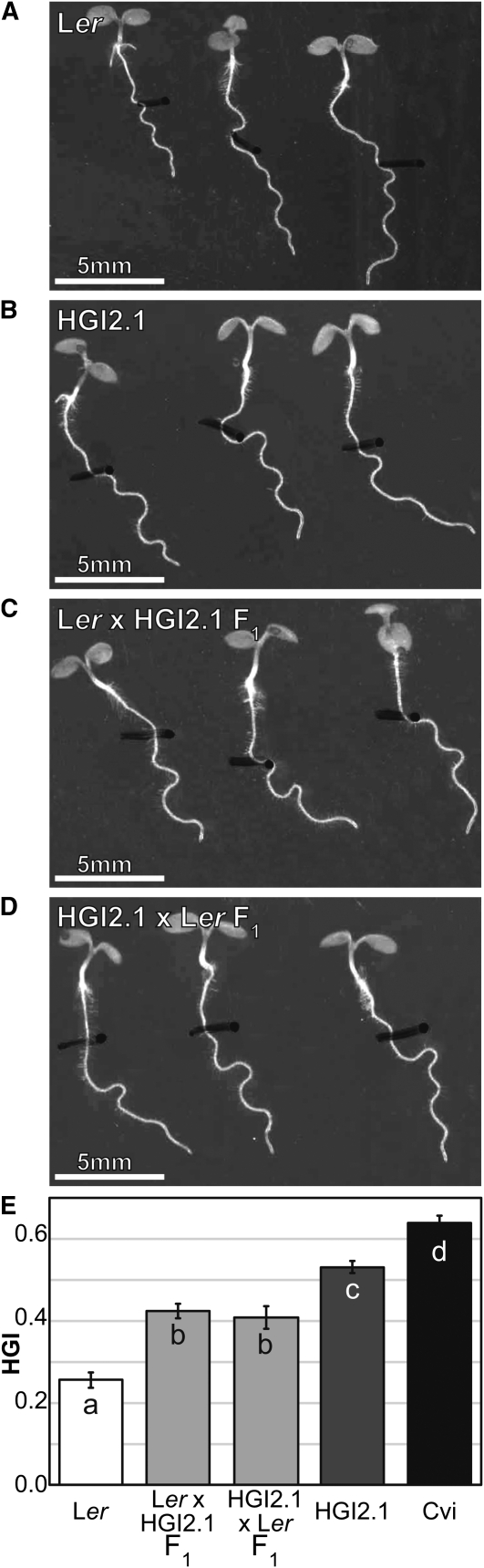
Phenotypes of L*er*, HGI2.1, and F1 progeny from crosses between L*er* and HGI2.1. (A–D) Representative roots for each genotype grown on the wave assay. (E) Means for each genotype for HGI. Statistically significant differences between genotypes are represented by distinct letter symbols in the corresponding graph bars (*P* < 0.05, pairwise *t*-test). Bars are ± SE. 28 to 93 seedlings of each type were measured. White bar = 5 mm.

To further break down this interval, HGI2.1 and HGI2.2 were back-crossed into L*er* as the first step to creating a mapping population. We took this opportunity to look at HGI in the progeny of this cross, as well as the reciprocal. The phenotype of both F1s is intermediate to and significantly different from both parents, indicating the QTL in this region is semidominant (Figure 6). In this case, the cross direction does not appear to influence the HGI value of the F1. Some of the heterozygotes from the L*er* × HGI2.1 and L*er* × HGI2.2 back-crosses were selfed to generate a mapping population of over 1300 individuals with multiple recombination events in the region of interest. However, fine mapping with the F2 lines has been difficult, leading us to believe the region is more complex than first indicated by the QTL maps. The three NILs that continually show a greater skew than L*er* over multiple trials all have very large Cvi introgression segments. When that segment is broken up in the mapping population within the candidate region, some lines are found to have significantly greater skew than L*er*, but less so than HGI2.1 or 2.2. This could mean that another part of the Cvi introgression is necessary for the full effect, and the two loci act in an additive manner. When separated, skew is lessened and more subject to environmental variance. The second region may lie in the introgression segment of LCN2-9 since it sometimes has a significantly greater HGI value than L*er*. If the second locus is contained in the LCN2-9 introgression, it would likely be toward the distal end where the LCN2-8 and LCN2-9 introgressions no longer overlap, because LCN2-8 never produced an HGI value significantly greater than L*er*. Work is underway to map the predicted causative locus in the region between 9.3 and 11.2 Mb.

### Expression differences Between Cvi, L*er*, and HGI2.1 root tips subjected to the wave assay

Because many QTL mapped to date have been due to changes in gene regulatory regions (for review, see Alonso-Blanco *et al.* 2009), we decided to look at gene expression differences in Cvi and L*er* roots subjected to the wave assay. The combination of the microarray data and the QTL data could narrow the pool of candidate genes. We also included HGI2.1 in the analysis. If the locus/loci causing increased skew from chromosome 2 were due to changes in expression, than HGI2.1 should also have those changes compared to L*er*.

For each of the three genotypes, Cvi, L*er*, and HGI2.1, 3 biological replicates of about 600 pooled root tips each were hybridized to Affymetrix ATH1 arrays. Linear correlations within replicates ranged from 0.90 to 0.91 for Cvi, 0.93 for L*er*, and 0.92 for HGI2.1. At the 95% confidence interval, 699 probe sets showed differential expression between Cvi and L*er* (Table S6). Of these sets, nine fell in the interval on chromosome 2 between 9.3 and 11.2 Mb, with a twofold or more change in expression level between the accessions (Table 1).

**Table 1:** Nine probe sets from a segment of chromosome 2 located between positions 9.3 and 10.2 Mb (Figure 6) show twofold or more differential expression between Cvi and L*er* at 95% confidence

Probe set ID	Gene Symbol	AGI	L*er* over Cvi Fold Change	log_2_ Expression Level			Description
				HGI2.1	L*er*	Cvi	
265438_at		AT2G20970	5.989 down	3.28873	0.60115	3.18369	Unknown protein
257432_at		AT2G21850	7.834 up	6.80522	10.3497	7.37979	Zinc finger
267131_at		AT2G23400	28.735 up	1.39873	6.62919	1.78445	Dolichol biosynthesis
267137_at	ACPT	AT2G23410	4.785 up	8.91825	10.50796	8.24931	Dolichol biosynthesis
267286_at		AT2G23640	4.172 down	5.00491	3.97473	6.03559	Reticulon family
263788_at		AT2G24580	2.695 down	10.28372	9.51868	10.94939	Sarcosine oxidase
263537_at	COL3	AT2G24790	2.768 up	9.78917	11.12704	9.65781	*constans*-like
263526_at		AT2G24830	2.655 up	7.9491	9.22268	7.81372	Zinc finger
263539_at	TAT3	AT2G24850	5.468 up	6.33535	7.97801	5.5268	Tyrosine aminotransferase

## Discussion

### Many loci are predicted to contribute to root growth behavior differences Between Cvi and L*er*

We observed a great deal of variation in the root growth behaviors of *A. thaliana* accessions on hard agar surfaces. One of the most noticeable behaviors was seen in the accession Cvi, which had the greatest rightward skew of all the lines that were assayed. The TAIR website (www.arabidopsis.org) documents that this accession was first collected growing on a “rocky wall with moss.” It may be that the skewing pattern its root takes along the hard agar surface reflects an adaptation to this type of soil environment where the root must grow over long spans of rocks for periods of time.

Our goal was to use natural genetic variation to find regions of the genome that contribute to root growth behaviors on hard surfaces in *Arabidopsis thaliana*. The accessions Cvi and L*er* differ in both skewing and waving, and so were good candidates for use in a QTL study to try and determine the genetic contributors to these differences. Through QTL mapping with the Cvi/L*er* RIL population, we have pinpointed several chromosomal regions predicted to modify root skewing and waving behaviors, as well as root length. A QTL for skewing on chromosome 2 is being pursued by fine mapping and expression analysis of the candidate interval.

For the traits we studied, many of the QTL were repeatable over three trials. QTL that did not appear in all studies are more likely to be subject to environmental and experimental variances, or are simply false positives. The differences could also be due to maternal effects that are affected by environmental conditions. Our results indicate there may be a cytoplasmic component to some of the traits we evaluated. Root skewing differed between reciprocal heterozygotes of Cvi and L*er*. Using Cvi as the female led to significantly greater skewing than that seen in crosses with L*er* as the female parent. In the QTL trials, only trial 3 showed any significant differences for trait means with respect to RIL cytoplasm donor. Because three traits were affected by maternal lineage in this trial, but not in the others, it may be that the experimental conditions of trial 3 were such that they exposed environmentally responsive maternal contributors to root behaviors.

We focused our efforts on characterizing the loci contributing to the difference in root skewing, in particular a region centered near 40 cM on chromosome 2. With the help of two sets of NILs, the candidate region has been narrowed to the area between 9.3 and 11.2 Mb. All of the NILs have L*er* cytoplasm, so there should be no maternal effects on skewing between them and L*er*. This was seen in reciprocal crosses between L*er* and HGI2.1.

Further fine mapping of the candidate region is underway. From the over 1300-line mapping population, we have identified 143 F3 lines that have a crossover in this interval (between markers LMV 166/167 and LMV 176/177; see Table S4) and should help us further locate genes that contribute to this QTL. Preliminary studies of this region indicate the possibility of multiple loci contributing to the QTL, acting in an additive fashion. We have looked at the wave assay phenotypes of several mapping population lines with Cvi introgressions of various lengths, broken down from the HGI2.1 introgression. Lines with nonoverlapping segments of Cvi in both the proximal and distal portions of the introgression have shown significantly greater HGI than L*er*. Also, the lines with a proximal or distal segment allowing for skew greater than L*er* usually skew significantly less the HGI2.1, indicating both segments are needed for the greater HGI seen in HGI2.1. The hypothesis that multiple regions on chromosome 2 are contributing to greater skew over Ler is also supported by the data from NIL2-7 and 2-9. As mentioned in results, LCN2-7 consistently showed a greater skew than L*er* over five trials, and LCN2-9 had a significantly greater skew than L*er* in two of these trials. So, along with the region between 9.3 and 11.2 Mb, a more distal segment on chromosome 2 that is contained in the Cvi introgression of LCN2-9, but not LCN2-8, appears to be contributing additively to root skewing on surfaces.

There are several cases described in plants where multiple changes in a gene region contribute to a particular QTL, for example the tb1 promoter region in maize (Clark *et al.* 2006) and others involving combinations of promoter and coding region changes (for review, see Alonso-Blanco *et al.* 2009). In this case, the loci seem to be further apart, involving a region encompassing more than just a single gene and its regulatory sequences. We hope to utilize the mapping population to separate and quantify the effects of the each QTL contributor, if possible.

A microarray study using root tips from Cvi, L*er*, and the NIL HGI2.1 predicted 699 expression differences between Cvi and L*er*. It should be noted that the probes on the ATH1 GeneChip (Affymetrix) are based on Columbia sequence, so probe-binding differences could also be due to polymorphisms changing hybridization efficiency. This may be helpful to identify polymorphisms within regions between the accessions. In the candidate region for the chromosome 2 QTL, nine genes are predicted to be differentially expressed.

### The chromosome 2 skewing QTL region contains candidate genes that may contribute to the QTL

There are 541 annotated genes in the region between 9.3 and 11.2 Mb, and, to our knowledge, none of them have been described previously as contributing to root skewing behavior. However, the region does contain a few genes related to known skew effectors, including a *SKU5*-similar gene, *SKS16*, and an extra large G protein *XLG1*. Both *sku5* and the G protein mutants *xlg3* and *agb1* have skewing phenotypes (Sedbrook *et al.* 2002, Pandey *et al.* 2008). The wave assay for several *sks16* T-DNA insertion lines did not show any discernable skewing phenotype (data not shown), and *xlg1* does not have a skewing phenotype as a single mutant (Pandey *et al.* 2008).

From our microarray study, the most interesting candidates are the adjacent loci At2g23400 and At2g23410. Probe sets associated with these loci displayed higher hybridization signals with root tip RNA derived from L*er* than Cvi and HGI2.1. These genes encode *cis*-prenyltransferase enzymes (Cunillera *et al.* 2000), which are part of the dolichol and dolichyl phosphate biosynthesis pathway (Grabiñska and Palamarczky 2002). At2g23410 has confirmed *cis*-prenyltransferase activity, and At2g23400 is predicted to have activity by sequence identity (Cunillera *et al.* 2000). From studies in yeast, the reaction catalyzed by *cis*-prenyltranferase commits the cell to dolichol biosynthesis (Adair and Cafmeyer 1987). One branch of this pathway produces dolichyl-phosphate-mannose, which is used for the GPI-anchoring of proteins, among other things (reviewed in Burda and Aebi, 1999). GPI serves as a membrane anchor for a variety of cell wall–associated proteins. One such GPI-anchored protein is SKU5. One of the *A. thaliana cis*-prenyltransferases, LEW1 (At1g11755), is predicted to glycosylate SKU5 (Zhang *et al.* 2008). When the *SKU5* coding region is mutated such that no protein is produced, seedlings exhibit an enhanced skewing over the wild-type (Sedbrook *et al.* 2002). SKU5 has been proposed to contribute to enzymatic reactions at the cell wall, and this could possibly affect the changes in cell wall composition when root cells are undergoing cell expansion. COBRA, another GPI-anchored protein, affects the patterning of cell wall microfibrils (Roudier *et al.* 2005). Mutant *cob* seedlings have defective anisotropic cell expansion and increased root skewing on hard surfaces (Benfey *et al.* 1993; Schindelman *et al.* 2001, Roudier *et al.* 2005). There is growing evidence that cortical microtubule arrays and cell wall microfibrils must act in concert to produce normal anisotropic cell expansion patterns (Paredez *et al.* 2006; Paredez *et al.* 2010). If Cvi is producing less of or altered forms of two *cis*-prenyltransferases, this could be affecting the amount of GPI donors available for proteins that should be anchored to interact with the cell wall. This change in cell wall composition would manifest itself in the root as an increase in root skewing to the right.

To begin the analysis of changes that may exist between Cvi and L*er* at the *cis*-prenyltransferase loci, we used POLYMORPH (http://polymorph-clark20.weigelworld.org/; Ossowski *et al.*, personal communication; Clark *et al.* 2007; Zeller *et al.* 2008). The output predicts many polymorphisms throughout the *At2g23400* gene and at the 3′ end of *At2g23410*. It also predicts polymorphic regions within the *At2g23400* gene, including one amino acid substitution, between L*er* and Columbia or Cvi (Figure S6).

It should be cautioned here that the Affymetrix ATH1 microarray contains probes for only 376 of the 591 annotated genes present on the QTL interval we mapped on chromosome 2. Therefore, it is also possible that other, yet unidentified genes within that interval are differentially expressed between Cvi an L*er* root tips, including additional potentially interesting candidates for this QTL. Further mapping and transgenic-rescue experiments will be needed to address this possibility.

This study has shown that natural variation can be harnessed to give us new information on *A. thaliana* root skewing and waving behaviors. A region on chromosome 2 between 9.3 and 11.2 Mb, which has no characterized skewing mutants, contributes to greater rightward skewing on tilted hard agar plates in Cvi compared to L*er*. Further experiments are underway to characterize the causative locus or loci, and preliminary analyses indicate two genes involved in dolichol/dolichyl biosynthesis may be contributing to the skewing difference, possibly by changing cell wall composition. The use of natural genetic variation to gain a greater understanding of how roots respond to gravity, touch, and other tropic stimuli may benefit us in the breeding of plants more suited to particular soil environments. As we try to produce high-yielding crops in diverse conditions, our knowledge of root growth behaviors and their modulation by external cues can help us in the endeavor.

## Supplementary Material

Supporting Information
