# Supplementary material for: A QTL Study for Regions Contributing to Arabidopsis thaliana Root Skewing on Tilted Surfaces
Source: G3 (Bethesda). 2011 Jul 1;1(2):105–15. doi: 10.1534/g3.111.000331 (PMC3276130; doi:10.1534/g3.111.000331)
Supplement: Supporting Information [file supp_1_2_105__index.html]

Supporting Information 

# A QTL Study for Regions Contributing to *Arabidopsis thaliana* Root Skewing on Tilted Surfaces

## Supporting Information for Vaughn and Masson, 2011

**Files in this Data Supplement:**

- Supporting Information - Figures S1-S6 and Tables S1-S9 (PDF, 2.4 MB)
- Figure S1 - QTL analysis of L, VGI, and angle B in the Cvi/*Ler* RIL population (PDF, 416 KB)
- Figure S2 - 2-D scans for all root growth parameters for trial 1 (PDF, 312 KB)
- Figure S3 - 2-D scans for all root growth parameters for trial 3 (PDF, 676 KB)
- Figure S4 - Trait-by-cytoplasm effects for the RILs (PDF, 72 KB)
- Figure S5 - Skewing phenotypes for chromosome 4 NILs (PDF, 54 KB)
- Figure S6 - Output from POLYMORPH in GBrowse (http://polymorph.weigelworld.org/; CLARK et al. 2007; ZELLER et al. 2008) for the regions near At2g23400 and At2g23410 for Cvi and *Ler* (PDF, 280 KB)
- Table S1 - Parental and RIL means, RIL mean ranges, and heritabilities over 3 trials for root growth behavior traits (PDF, 40 KB)
- Table S2 - Significant QTL positions over three trials for root growth behavior traits (PDF, 128 KB)
- Table S3 - Significance values for DOG 17 root trait means compared to *Ler* (PDF, 36 KB)
- Table S4 - Primers used for chromosome 2 fine mapping, indel, SSLP, and CAPs (PDF, 80 KB)
- Table S5 - Chromosome 2 Cvi introgression mapping in Cvi/*Ler* NILs (PDF, 44 KB)
- Table S6 - 699 Probe Sets at 95% Confidence for Differential Expression between Cvi and *Ler* (PDF, 384 KB)
- Table S7 - (Microsoft Excel, .xls, 252 KB)
- Table S8 - (Microsoft Excel, .xls, 556 KB)
- Table S9 - (Microsoft Excel, .xls, 492 KB)
