# Supplementary material for: A QTL Study for Regions Contributing to Arabidopsis thaliana Root Skewing on Tilted Surfaces
Source: G3 (Bethesda). 2011 Jul 1;1(2):105–15. doi: 10.1534/g3.111.000331 (PMC3276130; doi:10.1534/g3.111.000331)
Supplement: Supporting Information [file supp_1.2.105_000331SI.pdf]

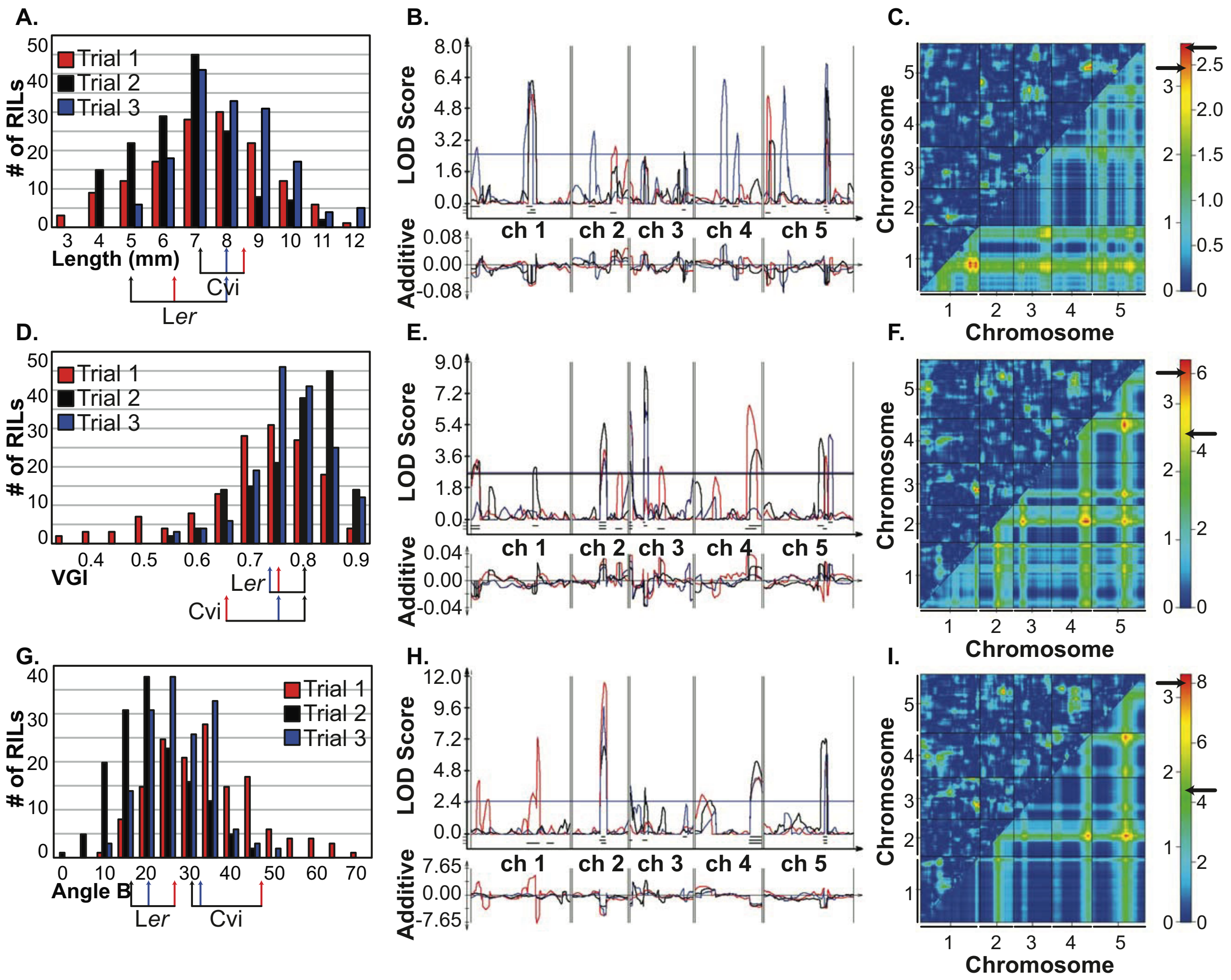

Figure S1 QTL analysis of L, VGI, and angle B in the Cvi/L*er* RIL population. Panels A, D, and G show the histograms of RIL means over the three QTL trials for L, VGI, and angle B, respectively. The color-coded arrows indicate the means for the Cvi and L*er* parents for each trial, "C" is next to the Cvi arrow and "L" is next to those for L*er*. Composite interval mapping (CIM) analysis over three trials for L is shown in panel B, VGI in E, and angle B in H. Trial 1 is red, trial 2 is black, and trial 3 is blue. The top portion of the graph gives the LOD score across the five chromosomes of Arabidopsis. LOD significance thresholds determined by 1000 permutations for each trial are shown as horizontal line across the graph. 2-LOD intervals for significant QTL peaks are black bars beneath the peaks. The bottom graph is an indication of the additive value towards the phenotype of each genomic region with respect to the L*er* allele. Two-dimensional QTL scans are shown in panel C for length, panel F for VGI, and panel I for angle B. Plots are for trial 2. The region of the plot below the diagonal gives the additive QTL model, while the region above the diagonal shows epistatic interaction analysis. Heat map LOD significance thresholds were determined by 1000 permutations. The black arrow on the left LOD scale is the significance threshold for the epistatic portion of the plot, and the arrow on the right side is the threshold for the additive part.

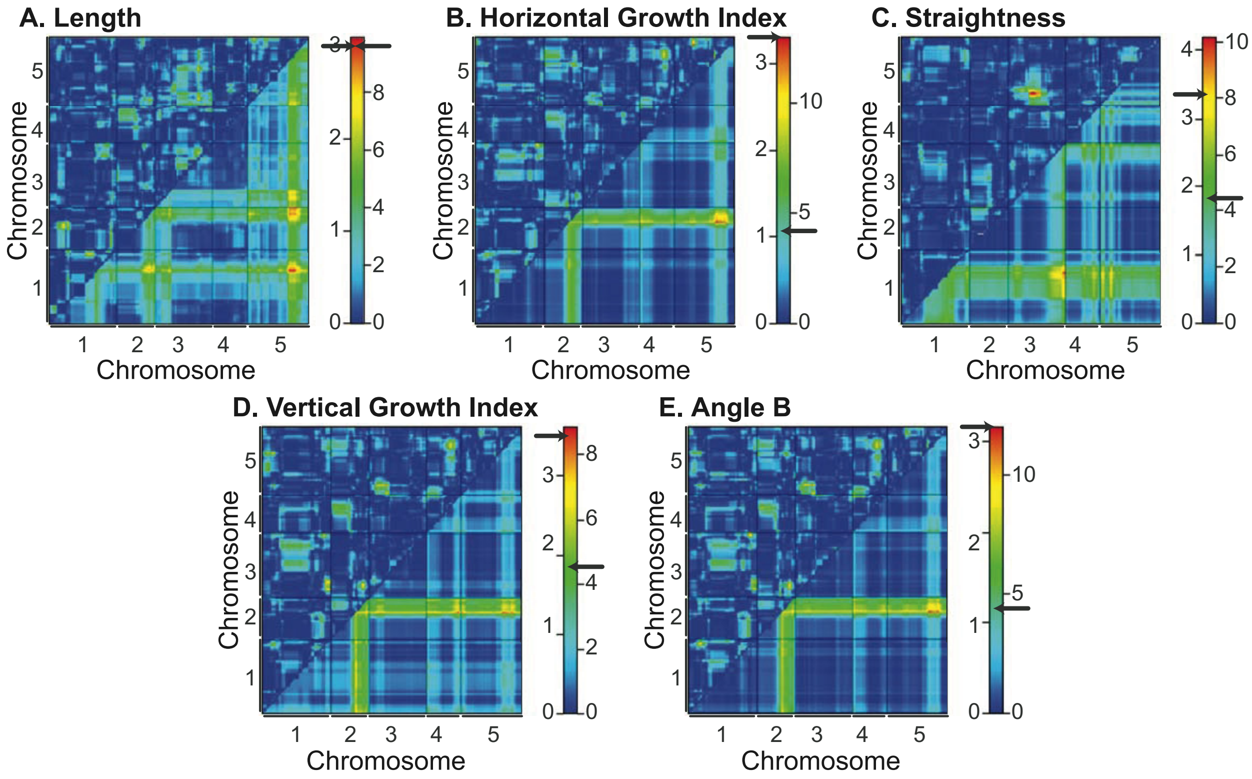

Figure S2 2-D scans for all root growth parameters for trial 1. Two-dimensional QTL scans are shown in panel A for L, B for HGI, C for Lc/L, D for VGI, and E for angle B. The axes represent positions along the five Arabidopsis chromosomes. The region of the plot below the diagonal gives the additive QTL model, while the region above the diagonal shows epistatic interaction analysis. Black arrows indicate significance thresholds with additive on the right and epistatic on the left. Thresholds were determined by 1000 permutation.

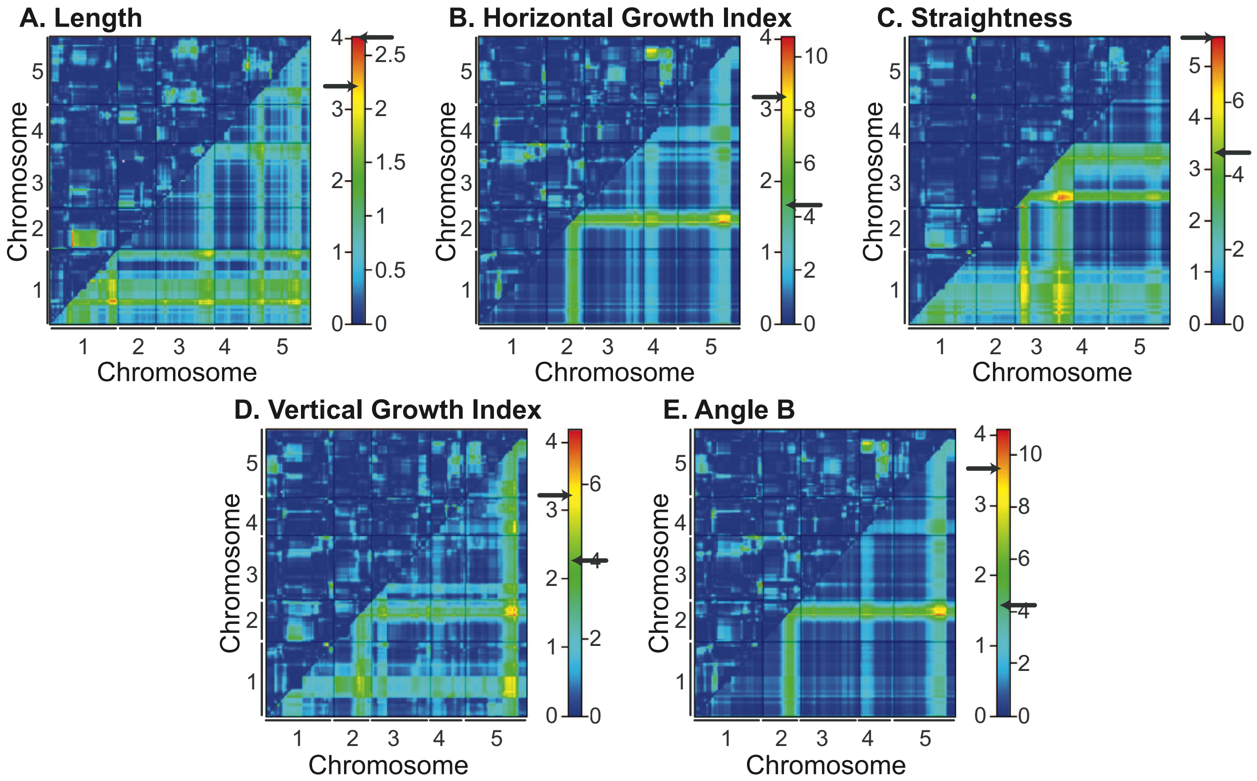

Figure S3 2-D scans for all root growth parameters for trial 3. Two-dimensional QTL scans are shown in panel A for length, B for horizontal growth index (HGI), C for straightness, D for vertical growth index (VGI), and E for angle B. The region of the plot below the diagonal gives the additive QTL model, while the region above the diagonal shows epistatic interaction analysis. Black arrows indicate significance thresholds with additive on the right and epistatic on the left. Thresholds were determined by 1000 permutations.

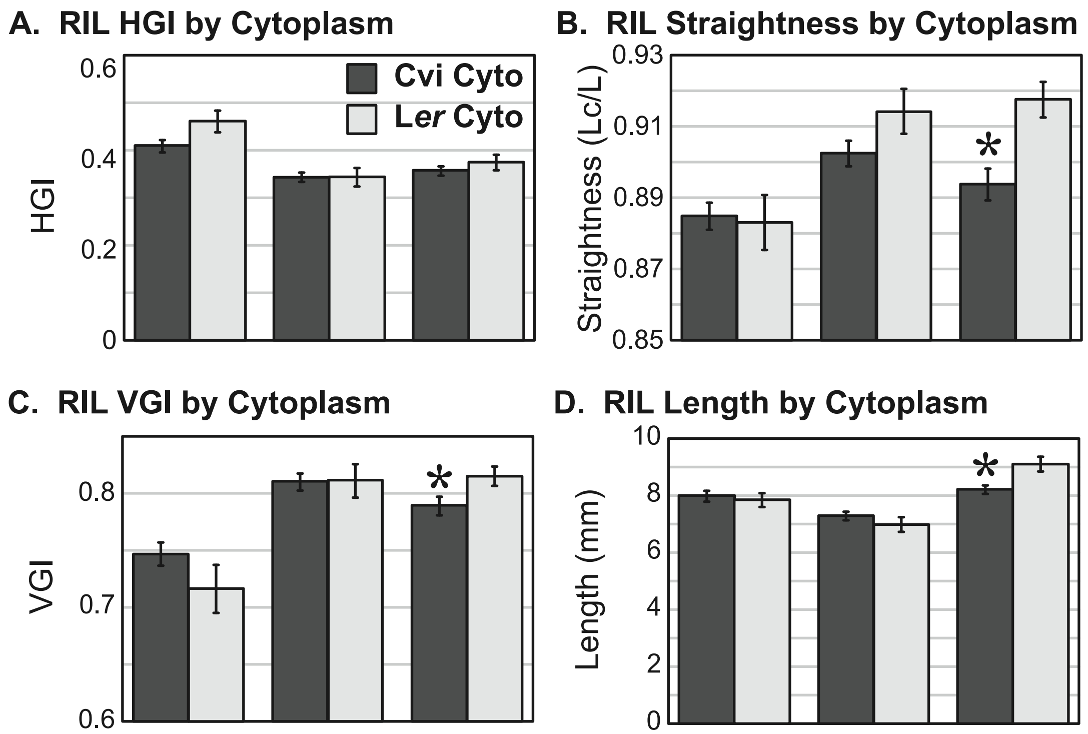

Figure S4 Trait-by-cytoplasm effects for the RILs. For each of three QTL trials, the root morphology measurements are compared based on Cvi and L*er* cytoplasm in the RILs. Traits shown include horizontal growth index (A), straightness (Lc/L), B, vertical growth index (C), and length (D). * indicates p-values less than 0.05 for comparison between the cytoplasm donor. Bars are +/- standard error.

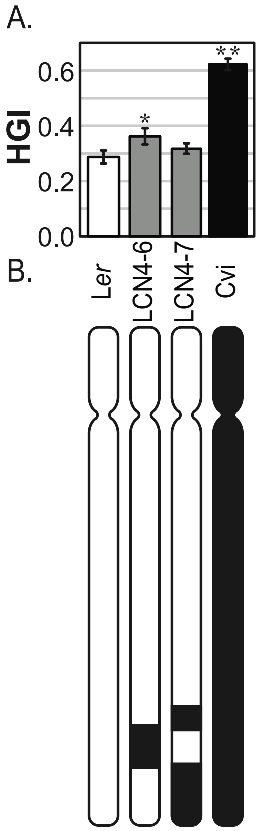

Figure S5 Skewing phenotypes for chromosome 4 NILs. Horizontal growth index (HGI) means for 2 NILs with Cvi introgressions near the end of chromosome 4 are shown in panel A. * indicates a p=0.05 for a skewing difference between L*er* and LCN4-6. ** indicates p<0.0001 between Cvi and L*er* for HGI. At least 30 seedlings were measured in each group. Bars are +/- standard error. Rough schematics of the Cvi introgression (in black) into chromosome 4 are shown in panel B based on the data in Keurentjes *et al.* 2006. LCN4-6 has one Cvi segment and LCN4-7 has two.

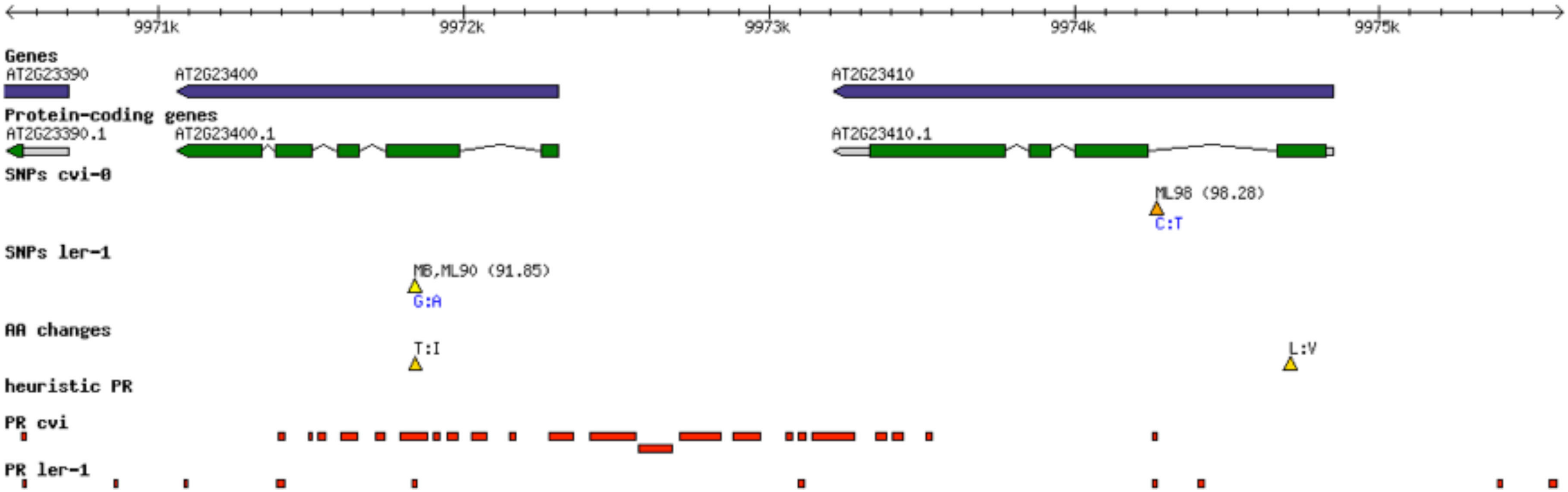

Figure S6 Output from POLYMORPH in GBrowse (http://polymorph.weigelworld.org/; CLARK *et al.* 2007; ZELLER *et al.* 2008) for the regions near At2g23400 and At2g23410 for Cvi and L*er*. Blue boxes represent annotated genes, and protein-coding genes are also displayed in green. Predicted SNPs are shown as triangles, and regions predicted to be polymorphic are highlighted by red bars. Cvi is predicted to be highly polymorphic relative to the Columbia accession over the sequence of At2g23400 and at the 3' end of At2g23410.

TABLE S1 Parental and RIL means, RIL mean ranges, and heritabilities over 3 trials for root growth behavior traits.

Trial 1	Cvi mean	Ler mean	RIL range	RIL mean	RIL ANOVA	H^2
Length	8.5	6.43	3.3 to 12.9	7.96	***	0.62
VGI	0.66	0.75	.36 to .91	0.74	***	0.52
HGI	0.62	0.22	.17 to .80	0.42	***	0.47
angle B	47.9°	25.8°	8.1° to 67°	33.9°	***	0.54
Straightness	0.92	0.81	.78 to .96	0.89	***	0.32

Trial 2	Cvi mean	Ler mean	RIL range	RIL mean	RIL ANOVA	H^2
Length	7.2	5.1	4.0 to 11.99	7.2	***	0.49
VGI	0.8	0.81	.56 to .96	0.81	***	0.36
HGI	0.44	0.24	0 to .61	0.34	***	0.29
angle B	28.5°	17.3°	0° to 48.7°	23.6°	***	0.31
Straightness	0.95	0.87	.75 to .98	0.91	***	0.31

Trial 3	Cvi mean	Ler mean	RIL range	RIL mean	RIL ANOVA	H^2
Length	8.5	8.5	4.0 to 13.2	8.4	***	0.45
VGI	0.77	0.75	.42 to .95	0.8	***	0.37
HGI	0.52	0.29	.11 to .65	0.36	***	0.31
angle B	34.2°	20.7°	7.2° to 55.8°	24.7°	***	0.32
Straightness	0.95	0.84	.64 to .97	0.9	***	0.34

*** indicates p-value less than .000001 for variance between RILs versus within a RIL

Broad sense heritability (H^2) determined by variance within RILs divided by variance between RILs

Table S2 Significant QTL positions over three trials for root growth behavior traits. Significance levels were determined by 1000 permutations of the data.

Length, Trial 1

Chromosome	Position (cM)	LOD	Phenotypic Effect (R^2)
1	76.5	7.3	0.12
2	69.5	2.7	0.05
3	18.5	5.2	0.08
5	4.5	6.4	0.10
5	78	3.7	0.05

Length, Trial 2

Chromosome	Position (cM)	LOD	Phenotypic Effect (R^2)
1	65	8.1	0.13
3	47.5	2.6	0.04
3	56.5	3.6	0.05
5	6	5.1	0.08
5	7.5	4.9	0.08
5	10.5	5.7	0.10
5	77.5	6.6	0.10
5	106.5	3.3	0.05

Length, Trial 3

Chromosome	Position (cM)	LOD	Phenotypic Effect (R^2)
1	70	2.9	0.05
1	72	3.5	0.05
2	52	4.5	0.08
2	54	4.8	0.08
4	26	3.3	0.05
5	3.5	3.2	0.05
5	8	3.1	0.05
5	78	8.5	0.15

Vertical Growth Index (VGI), Trial 1

Chromosome	Position (cM)	LOD	Phenotypic Effect (R^2)
1	7	3.7	0.05
2	40	4.1	0.06
2	60	2.7	0.04
3	0	5.7	0.09
3	39	3.4	0.05
4	25.5	2.7	0.04
5	78	3.9	0.06

Vertical Growth Index (VGI), Trial 2

Chromosome	Position (cM)	LOD	Phenotypic Effect (R^2)
1	5.5	2.6	0.05
1	10.5	3.1	0.05
2	40	4.9	0.08
3	0	4.1	0.07
3	18.5	7.9	0.13
4	75	3.8	0.07
5	71.5	3.9	0.07

Vertical Growth Index (VGI), Trial 3

Chromosome	Position (cM)	LOD	Phenotypic Effect (R^2)
2	40	3.3	0.06
3	0	3.5	0.06
3	21	3.5	0.07
3	24	4.3	0.08
5	82	6.9	0.12
5	83	7.1	0.13

Horizontal Growth Index (HGI), Trial 1

Chromosome	Position (cM)	LOD	Phenotypic Effect (R^2)
1	84	5.7	0.08
2	40.5	12.5	0.20
3	71	3.1	0.04
4	8	2.6	0.03
4	78	2.8	0.04
5	78	8.3	0.12

Horizontal Growth Index (HGI), Trial 2

Chromosome	Position (cM)	LOD	Phenotypic Effect (R^2)
1	118	2.8	0.04
2	39	10.1	0.19
3	0	4.2	0.07
3	19	4.1	0.06
4	77.5	6.3	0.1
5	79	4.7	0.07

Horizontal Growth Index (HGI), Trial 3

Chromosome	Position (cM)	LOD	Phenotypic Effect (R^2)
2	40	10.2	0.16
3	0	3.4	0.05
3	11.5	3.2	0.05
3	13.5	3	0.05
3	70	3.8	0.06
4	25	3.2	0.05
4	80	2.8	0.05
5	78.5	6.3	0.1
5	80.5	6.2	0.1

Angle B, Trial 1

Chromosome	Position (cM)	LOD	Phenotypic Effect (R^2)
1	90.5	4.4	0.08
2	40.5	3.9	0.06
2	62.5	3	0.05
4	8	2.7	0.04
4	73	4.4	0.07
5	78	5.5	0.08

Angle B, Trial 2

Chromosome	Position (cM)	LOD	Phenotypic Effect (R^2)
1	119	4.8	0.09
2	40	7.2	0.12
3	0	4.5	0.07
3	19	5	0.08
4	76	5.3	0.09

Angle B, Trial 3

Chromosome	Position (cM)	LOD	Phenotypic Effect (R^2)
2	39.5	10.9	0.2
3	0	4	0.06
3	21.5	3	0.05
3	23	4.2	0.06
4	76.5	3.2	0.05
4	80.5	3.3	0.05
5	79	6.3	0.09
5	80.5	6.3	0.1

Straightness, Trial 1

Chromosome	Position (cM)	LOD	Phenotypic Effect (R^2)
1	7	4.7	0.07
1	68	7.4	0.13
3	0	5.5	0.08
3	18.5	7.2	0.11
3	79.5	6.8	0.10
5	24.5	4.7	0.07

Straightness, Trial 2

Chromosome	Position (cM)	LOD	Phenotypic Effect (R^2)
1	12	4.5	0.07
1	72	5.6	0.08
3	17.5	6.7	0.11
3	70	3.8	0.06
5	25	2.7	0.04
5	70	3.5	0.06

Straightness, Trial 3

Chromosome	Position (cM)	LOD	Phenotypic Effect (R^2)
3	0	4.1	0.08
3	18.5	2.7	0.05
3	30	3.2	0.06
3	66.5	3.1	0.06
3	69.5	3.4	0.06

Table S3 Significance values for DOG 17 root trait means compared to L*er*

	Length	VGI	HGI	Straightness	Angle B
DOG 17-1	**		**		**
DOG 17-2	**	*			*
DOG 17 -3					*
DOG 17 -4		**	**		**

* = p<0.05

**=p<0.001

Table S4 Primers used for chromosome 2 fine mapping, indel, SSLP, and CAPs

CHROMOSOME 2 INDEL PRIMERS

Forward Primer	Reverse Primer	TAIR build 9 Forward Primer Start (bp)	Cvi product size	*Ler* product size	Source
GAGAGTCGCAAAAACCTTGG	AGAAGAAACCGCGACGAATA	2943966	1291	542	a
CTAGCAAGACGCATCAAGGG	CTAATGACGACTTCGAGAAAGTT	5797945	247	202	a
CAATGTTTTGTGGGCTGTTG	CGCAATTGTTAATGTGGTTCC	6370922	529	481	a
GGTGTCAAAGTTTCCGTCACACAC	ACTACAGAGGCTTACGAGGCCAAA	7342710	200	155	a
CTGGGGTGGAAAGAATCTGA	CACCGGTGTAACctttagcc	7715949	136	100	b
cgtctccgataactagtggatg	TCTTCGGTTATTAGGAGTGTG	8139986	160	172	b
TTGTGAAGGGTTTGAGTCCA	GCGCCGGAGAAGACTATGTA	8997379	384	423	a
GCCATGTTTGAAGGCATTGT	GATCTGCAGACGAGGGAAAG	9267216	717	550	a
aaccctatcaacgggcagaag	TATGGACATAGTCTCGGCATG	9347455	388	335	b
AACCAGTTATGGCCTTTCCTT	TTCCACGTAAGCCAAATTCC	9290093	550	422	a
GTTAGCTCACTCCAAATCGAGAAG	GTTTCATCGTACTTGCTTGGGAGG	9347566	353	300	a
AGCCAAATGCCAAATTCAAG	CTGGAATTCAGTTGGCCATT	9659301	332	284	a
ccttatatgaaaacagagagggtca	GGACAAGACCCGACTTCTGA	10020562	410	~380	c
TGTTGGTAGGTCGCCCTCT	GCCATTGCAAAAGAACATCA	10026853	437	470	a
cgacttcttactcttcaccgaca	tgttgtgtcattgtgaacatgg	10046432	~450	499	c
cggtcggatctgaatacattg	gccacatagggatagttgcag	10199101	200	177	a
aagtttcaacccatcaaataaataga	tatattggcgttgccgacac	10430711	200	179	a
ggaattttccttcgtgttttca	cagtttgtgtacgggatgaat	10560001	400	361	a
GATTTACATATGCCAATCCG	CTTCCGTCTCTGTCTCAAACTG	10866073	251	226	a
TCGACCGGAAAACAAGATTC	acaaattcaggtgttagctgttt	10934438	400	347	a
tcgtactgaccgacctttga	tggaatcgtgtttctcctga	11194426	166	154	a
TTCTCACCGAAATCGATGCT	TCAGTGCATAGTGAGTAAgattgga	11221229	391	~360	c
GCTTCTCTGGACACACCTCA	GCGAATCTCGTGGTTACTCC	11232714	195	179	a
TGAATATGCAGGATACGACTGTG	TGCTAATTTATACGCATCTGTCTC	11446331	381	~360	c
cactttatgttttgggtggtaca	cgttccaagataatgtaagtgct	12404780	226	210	a

caacggcctttcaagagttc	ttagcggcagtggttatcaa	13237730	187	172	a
tggcaaatatgtagccagaaa	acacatccccacatgccta	13498360	109	100	a
tccattcaatatttcgtctcg	acacggtttagcaacgatct	13786899	114	104	a
TGATTTCTCCTTACCTGAAAAAGG	TTCAACTGTTGTCATTTCATCAT	14118356	466	419	a
CTTTATGTGCCGAAGTTCAAA	CATGGTTAGGCCCGTTAAGA	15293730	475	444	a
cactagtaagatcttgttgatg	gatggtcattttgagattcatag	15579317	244	172	a
ATCCCTGAACTTCACATCTGCTGC	TCACTCCTCTTGGTTTCAGTAGTGG	16465929	200	161	a
GCCATTGTTCGATTTGCTTTGGTTTGG	GGGCTTGCAACATTGTAAATAGGC	16474309	318	293	a
tgttgagtgttcaaacggtga	aacgttcgtggagtatacttttct	17061306	127	113	a
ttctgaaattagtatttgtgccgta	tcaatcattaaaaccgtttcctg	17421092	208	192	a
CGTCACCGCAATTTCACTAA	TGATCCTGATTTGGGTCTCC	18307407	630	574	a
TTGTGCATCGCTTTCAGCATTCGG	AGTTGTAATATGGCAACCGCAGCC	18891919	360	317	a

CHROMOSOME 2 CAPs PRIMERS

		**TAIR build 9**				
		**Forward Primer**				
**Forward Primer**	**Reverse Primer**	**Start (bp)**	**Cvi product size**	**L*er* product size**	**Source**	**Enzyme**
TGGGTGTTCACAGGATCTCA	AGCTCTGACGCAACCTCATT	9957327	~280 and 520	807	d	BanI
agccacgaaccaaaaattatc	cttttggctctccagctgat	11039713	176 and 374	550	a	SspI
CATTGTTCTCTTCCAGGAAACTT	AAACAAAGCATGACTCTGATCAAT	11148407	122 and 224	346	a	AluI
TTACAAGCATCATGGAGACAGAg	TCCAAGTAGTGGCGGTCTTT	11153101	216 and 384	600	a	BsbRI
AGCTTCACCTCCAACTTCCA	CGTGAAGAAGCCTCTACAGTGA	11171336	617	~180 and 440	d	Hpy188I
AAACTCCACAAAAGCACAAACA	TCCAAACCCAAATCTTGACC	11683970	940	~350 and 590	d	Tsp45I
GCTTGGAGCAGCCACATTAC	GCGCTAATTATTTCGGGACA	13082931	~180 and 150	334	d	BbvI
Cctgcaagaatcgagatcca	ATCAGGGCTTGTGGTGGTAG	13748944	~400 and 550	946	d	BsmAI

a: Source is CEREON/Monsanto Col/L*er* polymorphisms

b: Source is Julin Maloof

c: Source is POLYMORPH Bur/Tsu PRs

d: Source is TAIR polymorphism bank

Table S5 Chromosome 2 Cvi introgression mapping in Cvi/L*er* NILs

NIL Name	L*er* to Cvi beakpoint unknown (bps)	Known Cvi introgression (bps)	Cvi to L*er* breakpoint unknown (bps)
LCN2-4	0 - 2943966	2943966 - 8997379	8997379 - 9347566
LCN2-6	6370922 - 7342710	7342710 - 8997379	8997379 - 9347566
LCN2-7	6370922 - 7342710	7342710 - 16465929	16465929 - 17421092
HGI2.3	6370922 - 7342710	7342710 - 8139986	8139986 - 8997379
HGI2.4	7342710 - 7715949	7715949 - 9347566	9347566 - 9659301
HGI2.5	7342710 - 7715949	7715949 - 9347566	9347566 - 9659301
HGI2.2	7715949 - 8139986	8139986 - 16465929	16465929 - 17421092
HGI2.1	7715949 - 8139986	8139986 - 16465929	16465929 - 17421092
LCN2-8	10934438 - 11194426	11194426 - 13082931	13082931 - 13748944
LCN2-9	10934438 - 11194426	11194426 - 15293730	15293730 - 15579317

Table S6 699 Probe Sets at 95% Confidence for Differential Expression between Cvi and L*er*

			L*er* over Cvi	log2 Expression Level		
Probe Set ID	Gene Symbol	AGI	Fold change	HGI2.1	L*er*	Cvi
261577_at		AT1G01080	2.177 down	7.18	7.24	8.37
261581_at	CIPK9	AT1G01140	1.798 down	8.15	8.25	9.09
261537_at		AT1G01800	2.266 up	12.90	12.94	11.76
259416_at		AT1G02305	3.198 up	12.73	12.84	11.16
259415_at		AT1G02330	1.486 up	9.32	9.54	8.97
262107_at		AT1G02750	3.100 up	9.66	9.61	7.98
262118_at	BGLU11	AT1G02850	2.236 up	11.03	11.13	9.97
264354_s_at		AT1G03200	6.361 up	6.69	7.52	4.85
264830_at		AT1G03710	116.089 down	4.05	4.26	11.12
263182_at		AT1G05575	2.903 up	9.07	8.88	7.34
262625_at		AT1G06440	2.098 down	6.80	6.45	7.52
262616_at		AT1G06620	2.387 up	10.31	11.04	9.79
262637_at		AT1G06640	4.746 up	12.82	12.82	10.57
256053_at	UGT71C3	AT1G07260	2.358 up	8.78	8.90	7.66
261785_at		AT1G08230	26.732 up	6.19	6.67	1.93
261720_at	HDA08	AT1G08460	1.327 down	8.89	9.08	9.49
261696_at		AT1G08470	1.549 down	10.60	10.44	11.07
264802_at	SYP111	AT1G08560	1.968 up	11.38	11.43	10.45
264811_at		AT1G08640	1.724 down	10.04	9.90	10.69
264665_at		AT1G09660	1.306 down	10.82	10.72	11.10
260480_at	SNRK2.4	AT1G10940	2.997 up	10.12	10.28	8.70
262452_at		AT1G11210	2.180 up	8.22	8.86	7.74
262456_at	STP1	AT1G11260	1.834 up	12.25	12.52	11.64
261846_at		AT1G11540	2.733 up	9.13	9.40	7.95
262811_at		AT1G11700	1.778 down	8.61	8.45	9.28
260967_at		AT1G12230	1.530 down	11.23	11.30	11.91
259536_s_at	ATCOAB	AT1G12350	1.644 up	10.23	10.14	9.42
259529_at		AT1G12400	11.508 down	6.31	5.27	8.80
261201_at		AT1G12850	2.478 down	9.73	9.88	11.19
259366_at	AOC4	AT1G13280	2.365 up	12.22	12.49	11.25
256134_at	AAPT1	AT1G13560	1.149 up	12.39	12.25	12.05
256070_at		AT1G13730	1.652 down	11.88	11.58	12.30
262655_s_at		AT1G14185	2.510 down	7.52	7.48	8.80
261474_at		AT1G14540	2.776 up	10.53	10.23	8.76
259502_at		AT1G15670	2.343 down	10.76	10.94	12.17
262712_at	ATRDH2	AT1G16460	3.634 up	10.63	10.55	8.69
261087_at		AT1G17350	4.193 up	10.79	10.84	8.77
256073_at	E12A11	AT1G18100	5.518 down	5.36	5.69	8.15
259481_at	GLP4	AT1G18970	4.347 up	10.51	10.35	8.23
259478_at		AT1G18980	8.351 up	13.19	13.05	9.98
256012_at	FMO1	AT1G19250	21.180 up	7.90	8.50	4.09
261226_at	ATEXPA11	AT1G20190	2.584 up	10.90	11.10	9.73
261459_at		AT1G21100	5.048 down	9.34	9.86	12.20
261450_s_at		AT1G21110	21.725 up	11.01	11.14	6.70
261448_at		AT1G21140	2.233 up	9.30	9.07	7.91
260902_at		AT1G21440	2.225 down	9.92	9.99	11.15
255968_at		AT1G22270	2.521 up	11.00	10.79	9.45
255942_at	AtUGT85A2	AT1G22360	9.762 up	12.00	12.06	8.77
261927_at		AT1G22500	1.793 up	10.93	11.17	10.33
264198_at		AT1G22800	1.637 down	9.61	9.39	10.10
264202_at		AT1G22810	2.759 up	6.78	6.63	5.17

Probe Set ID	Gene Symbol	AGI	L*er* over Cvi Fold change	log_2 HGI2.1	Expression L*er*	Level Cvi
264773_at		AT1G22900	10.017 up	7.12	6.78	3.46
257412_at		AT1G22980	4.590 down	6.38	6.27	8.47
264893_at		AT1G23140	3.076 up	9.06	8.96	7.34
263032_at		AT1G23850	10.596 up	8.09	8.61	5.21
263033_s_at		AT1G23950	2.184 down	11.36	11.24	12.37
263028_at		AT1G24030	4.125 down	6.91	6.81	8.85
265017_at		AT1G24390	16.790 up	6.88	6.43	2.36
245650_at		AT1G24735	5.543 down	6.91	6.76	9.23
245651_s_at		AT1G24793	2.539 up	11.52	11.65	10.30
245646_x_at		AT1G24851	6.189 up	8.41	8.54	5.91
255731_at	RCN1	AT1G25490	1.364 down	12.68	12.56	13.01
245873_at	CIB5	AT1G26260	1.800 down	7.56	7.53	8.38
265003_at		AT1G26970	3.914 up	8.52	8.75	6.78
264987_at		AT1G27030	2.518 up	13.10	13.28	11.94
264977_at		AT1G27090	1.546 down	11.27	11.10	11.73
264979_s_at		AT1G27170	3.144 down	6.60	6.51	8.16
262319_s_at		AT1G27540	13.053 up	13.07	13.30	9.59
259606_at	MAP65-8	AT1G27920	4.194 up	8.45	8.67	6.60
255995_at	RKF1	AT1G29750	1.871 down	7.91	7.57	8.47
257418_at		AT1G30850	2.033 down	8.14	8.22	9.25
265130_at		AT1G30890	2.257 down	10.50	10.49	11.66
265155_at		AT1G30990	14.989 down	7.07	7.25	11.16
263701_at		AT1G31160	4.205 up	9.45	9.46	7.39
263695_at		AT1G31220	2.436 down	8.24	7.77	9.05
256492_at		AT1G31490	7.800 down	4.86	4.26	7.23
256493_at		AT1G31600	2.812 down	8.54	8.66	10.15
246259_at		AT1G31830	1.671 down	9.53	9.53	10.27
245792_at	PRR1	AT1G32100	1.760 up	12.02	12.11	11.29
252522_at		AT1G32337	9.993 down	1.00	-1.12	2.20
260652_at		AT1G32360	1.953 up	9.06	9.23	8.27
261239_at		AT1G32930	2.560 up	9.09	9.25	7.89
261616_at	ANAC014	AT1G33060	1.505 down	7.89	7.88	8.47
261596_at		AT1G33080	3.077 down	6.68	6.86	8.48
256531_at		AT1G33320	5.602 down	6.20	6.04	8.53
261985_at		AT1G33750	203.229 up	10.40	10.36	2.70
262564_at		AT1G34330	5.544 up	9.00	9.22	6.75
262561_at		AT1G34340	2.101 down	8.28	8.22	9.29
261156_s_at		AT1G34490	4.827 up	9.03	8.94	6.66
261157_at		AT1G34510	434.134 up	10.92	11.19	2.42
259576_at		AT1G35330	3.325 down	8.76	8.63	10.37
259577_at		AT1G35340	3.640 up	8.82	8.91	7.05
263192_at	ACC1	AT1G36160	6.885 up	10.41	10.46	7.68
251905_at	GAPB	AT1G42970	4.804 down	8.11	8.44	10.71
264415_at	RAP2.6	AT1G43160	7.020 up	9.23	9.20	6.39
264421_at	ARP1	AT1G43170	3.019 down	13.30	13.32	14.91
262725_at		AT1G43580	1.587 down	10.18	10.17	10.84
245737_at		AT1G44160	4.260 up	7.78	8.12	6.03
245245_at	hemb2	AT1G44318	3.964 up	10.85	10.25	8.26
261335_at		AT1G44800	3.921 down	12.19	11.68	13.65
245803_at	RD21	AT1G47128	1.405 down	13.30	13.36	13.85
260730_at	mtLPD1	AT1G48030	1.617 down	13.35	13.18	13.87
262416_at		AT1G49390	5.889 up	8.09	8.38	5.82
261603_at	ATRBP47A	AT1G49600	1.317 down	12.84	12.71	13.11
261609_at		AT1G49740	2.095 up	9.57	9.76	8.69

262421_at		AT1G50290	7.695 up	9.10	9.47	6.53

Probe Set ID	Gene Symbol	AGI	*Ler* over Cvi Fold change	log$_2$ HGI2.1	Expression *Ler*	Level Cvi
261878_at	CYP705A25	AT1G50560	2.776 up	11.22	11.38	9.90
265136_at		AT1G51270	13.588 up	4.68	5.64	1.88
265142_at	DABB1	AT1G51360	2.965 down	8.23	7.96	9.53
260486_at		AT1G51550	1.441 down	7.05	6.75	7.28
256178_s_at	ILL5	AT1G51760	1.301 up	9.80	10.06	9.68
259841_at		AT1G52200	3.238 up	12.37	12.47	10.77
261342_at		AT1G52950	342.777 down	0.88	1.25	9.67
260617_at		AT1G53345	3.064 down	6.42	6.23	7.85
262198_at	ATPME2	AT1G53830	2.322 up	10.56	10.75	9.54
263156_at		AT1G54030	1.727 up	12.75	13.05	12.26
263145_at	ATEXO70D2	AT1G54090	4.950 up	9.67	9.91	7.60
263007_at		AT1G54260	10.597 up	9.05	9.11	5.71
263008_at	RPP27	AT1G54470	4.438 up	3.54	4.11	1.96
264191_at		AT1G54730	2.044 up	8.35	8.59	7.56
264185_at		AT1G54780	2.083 up	8.91	9.41	8.35
256352_at	ATPRP1	AT1G54970	2.782 up	11.43	11.62	10.14
265123_at		AT1G55440	5.128 up	7.95	7.68	5.32
265070_at	BCDH BETA1	AT1G55510	2.949 up	9.33	9.31	7.75
264536_at	PRA1.G1	AT1G55640	9.851 up	8.06	7.59	4.29
262095_at		AT1G56090	2.337 up	9.14	9.14	7.92
245676_at		AT1G56670	2.120 down	6.43	6.11	7.19
245657_at		AT1G56720	3.110 up	9.17	9.53	7.89
246410_at		AT1G57760	2.106 up	9.04	9.75	8.68
246399_at		AT1G58110	1.649 up	9.97	10.00	9.28
246394_at		AT1G58160	6.614 down	6.10	6.41	9.13
262897_at	CCB4	AT1G59840	2.062 down	6.23	6.18	7.23
262908_at	AT-E1 ALPHA	AT1G59900	22.342 down	7.34	7.06	11.54
262913_at		AT1G59960	2.580 down	9.06	8.96	10.33
264217_at		AT1G60190	2.365 up	7.29	7.82	6.57
264920_at	ECHID	AT1G60550	3.290 up	9.04	9.27	7.56
264932_at		AT1G61240	3.712 down	7.88	7.86	9.75
264881_s_at		AT1G61270	2.383 up	6.54	6.86	5.60
264754_at		AT1G61400	2.502 down	6.65	5.91	7.23
265035_at		AT1G61620	1.303 up	10.54	10.38	10.00
264398_at		AT1G61730	5.140 down	9.09	8.95	11.31
264731_at		AT1G62150	2.549 down	8.36	8.31	9.66
265114_at	LRX2	AT1G62440	2.331 down	5.94	6.00	7.22
265120_at	KAS III	AT1G62640	2.469 down	10.91	11.11	12.42
259692_at		AT1G63080	2.409 up	7.13	6.84	5.57
262340_at	ANAC027	AT1G64100	3.413 up	9.28	9.30	7.52
262353_at		AT1G64210	3.505 down	5.90	5.80	7.61
261957_at	ATMGL	AT1G64660	3.056 up	7.84	7.39	5.78
262865_at	CYP89A7	AT1G64930	3.021 down	8.40	7.84	9.43
266155_at	CYP89A5	AT1G64940	107.667 down	4.68	4.59	11.34
262875_at	G-TMT	AT1G64970	1.709 down	8.11	7.72	8.49
264158_at	PTAC4	AT1G65260	2.017 up	10.05	10.33	9.32
264159_at		AT1G65270	1.582 up	11.56	11.73	11.07
262933_at	ATPAO4	AT1G65840	2.109 down	12.37	12.30	13.38
257583_at	PMI2	AT1G66480	1.830 up	9.44	9.63	8.75
256376_s_at	PXMT1	AT1G66690	2.947 down	7.55	7.41	8.97
264970_at		AT1G67280	2.029 up	10.75	10.83	9.81
264229_at		AT1G67480	1.399 down	11.77	12.01	12.50
245215_at	ATFXG1	AT1G67830	6.045 down	5.28	5.93	8.53

260401_at		AT1G69840	2.472 down	9.07	8.92	10.23

			Ler over Cvi	log_2	Expression	Level
**Probe Set ID**	**Gene Symbol**	**AGI**	**Fold change**	**HGI2.1**	***Ler***	**Cvi**
260411_at		AT1G69890	3.363 up	7.37	7.63	5.88
264720_at		AT1G70080	20.814 up	10.13	10.26	5.88
260207_at		AT1G70730	4.658 up	10.90	10.84	8.62
259900_at	CSN5B	AT1G71230	1.591 down	8.57	8.45	9.12
259941_s_at		AT1G71280	4.430 down	5.38	5.23	7.38
260172_s_at		AT1G71920	1.944 down	10.37	10.27	11.23
259911_at	CAD1	AT1G72680	1.398 down	11.28	11.30	11.78
262362_at		AT1G72840	3.957 up	7.54	7.42	5.44
260097_at	AtOCT1	AT1G73220	72.674 down	4.30	3.71	9.89
260101_at		AT1G73260	12.186 up	12.65	12.57	8.96
245736_at	ATDR4	AT1G73330	5.139 down	10.66	10.91	13.28
245731_at	MKK9	AT1G73500	8.916 up	9.90	10.07	6.91
260072_at		AT1G73650	1.603 up	11.29	11.38	10.70
260234_at		AT1G74460	1.433 down	12.12	11.96	12.48
262171_at	TIFY10B	AT1G74950	1.544 up	10.81	11.08	10.46
262686_at		AT1G75990	1.343 down	10.97	10.94	11.37
259977_at		AT1G76590	3.106 up	8.15	8.29	6.65
259979_at		AT1G76600	1.612 up	10.50	10.20	9.51
259866_at		AT1G76640	14.251 up	6.37	7.44	3.61
264957_at	SKP2B	AT1G77000	3.710 up	9.68	10.06	8.17
259705_at	anac032	AT1G77450	2.008 up	12.09	12.13	11.12
260058_at		AT1G78100	1.494 down	10.13	10.10	10.67
260806_at		AT1G78260	2.915 down	9.38	9.41	10.95
264100_at	LUP1	AT1G78970	48.544 down	2.49	1.91	7.51
264131_at		AT1G79150	1.780 down	10.86	10.88	11.72
264144_at	AtMC6	AT1G79320	2.195 down	7.38	7.40	8.54
257475_at		AT1G80880	1.846 down	7.35	7.48	8.36
261901_at	J8	AT1G80920	3.384 up	12.06	12.38	10.62
265741_at		AT2G01320	1.703 down	7.52	7.74	8.51
266202_at		AT2G02400	5.737 up	10.92	10.82	8.30
267220_at	ISPD	AT2G02500	2.882 down	8.60	8.56	10.09
267240_at		AT2G02680	13.843 up	8.65	8.82	5.03
267479_at		AT2G02690	30.411 down	3.16	2.45	7.38
266743_at	RNS1	AT2G02990	3.002 down	7.97	8.48	10.06
265718_at	WRKY3	AT2G03340	2.016 up	8.80	9.29	8.28
265707_at		AT2G03390	1.787 up	9.51	9.55	8.72
264032_at	GEK1	AT2G03800	1.381 down	9.66	9.51	9.97
265651_at		AT2G13900	4.285 down	3.07	4.25	6.35
263276_at	CYP705A13	AT2G14100	23.289 up	10.20	10.61	6.07
265575_at	PIP	AT2G14260	6.049 down	9.30	9.17	11.76
266588_at	AGP9	AT2G14890	4.332 up	13.72	13.65	11.53
265486_at		AT2G15560	18.358 down	5.08	6.84	11.04
263098_at		AT2G16005	8.224 up	13.77	13.99	10.95
263607_at		AT2G16270	2.492 up	7.44	8.01	6.70
266525_at	MEE15	AT2G16970	9.058 down	7.74	7.97	11.15
263417_at		AT2G17180	1.435 down	2.25	1.51	2.03
263419_at		AT2G17220	3.201 down	8.40	8.44	10.12
264877_at	CYP51G2	AT2G17330	3.707 up	9.40	9.12	7.23
264904_s_at	NTRA	AT2G17420	3.011 down	11.65	11.51	13.11
264594_at	ATSERAT3;1	AT2G17640	1.946 down	7.79	8.19	9.15
264790_at	ATHK1	AT2G17820	2.272 down	8.79	8.67	9.86
265817_at	HIS1-3	AT2G18050	7.628 up	7.37	7.94	5.01
265929_s_at		AT2G18560	1.861 down	8.36	8.17	9.07

266693_at	MIOX2	AT2G19800	2.283 up	7.95	8.87	7.67
			**L*er* over Cvi**	**log_2**	**Expression**	**Level**
**Probe Set ID**	**Gene Symbol**	**AGI**	**Fold change**	**HGI2.1**	**L*er***	**Cvi**
266680_s_at		AT2G19850	314.826 down	3.77	2.02	10.32
265586_at	PR-1-LIKE	AT2G19990	13.491 down	4.77	3.02	6.77
265395_at	SRF1	AT2G20850	3.022 down	8.52	7.49	9.08
265438_at		AT2G20970	5.989 down	3.29	0.60	3.18
257432_at		AT2G21850	7.834 up	6.81	10.35	7.38
267263_at		AT2G23110	1.628 down	5.19	4.72	5.42
267131_at		AT2G23400	28.735 up	1.40	6.63	1.78
267137_at	ACPT	AT2G23410	4.785 up	8.92	10.51	8.25
267286_at		AT2G23640	4.172 down	5.00	3.97	6.04
267288_at		AT2G23680	1.961 down	8.79	7.91	8.88
267297_at		AT2G23780	1.349 up	10.26	10.55	10.12
265689_at		AT2G24310	1.581 up	7.20	7.87	7.21
263788_at		AT2G24580	2.695 down	10.28	9.52	10.95
263537_at	COL3	AT2G24790	2.768 up	9.79	11.13	9.66
263526_at		AT2G24830	2.655 up	7.95	9.22	7.81
263539_at	TAT3	AT2G24850	5.468 up	6.34	7.98	5.53
265897_at	MOT1	AT2G25680	5.404 down	6.84	5.45	7.88
265898_at		AT2G25690	2.051 down	8.58	7.81	8.85
266647_at		AT2G25870	1.632 up	7.59	8.37	7.66
266895_at		AT2G26040	3.323 up	8.42	8.92	7.19
267374_at		AT2G26230	2.331 up	10.11	11.56	10.34
267606_at	KCS11	AT2G26640	2.522 up	9.09	10.80	9.47
266310_at	FUS12	AT2G26990	2.368 up	9.24	10.89	9.64
250612_s_at	ARR13	AT2G27070	6.329 up	3.96	5.38	2.72
263083_at	PAP12	AT2G27190	5.856 up	10.02	12.18	9.63
265666_at		AT2G27440	9.117 down	7.25	4.87	8.06
266209_at	ATC	AT2G27550	2.496 up	10.18	10.61	9.29
266262_at		AT2G27590	2.593 up	9.34	10.39	9.01
266260_at	SKD1	AT2G27600	3.318 up	10.08	11.84	10.11
263438_at		AT2G28660	3.579 up	7.07	9.02	7.18
266276_at	TRI	AT2G29330	5.580 up	7.56	10.14	7.66
266296_at	ATGSTU7	AT2G29420	2.951 up	11.16	13.19	11.63
266271_at	ATGSTU6	AT2G29440	4.074 up	9.88	11.21	9.18
266294_at		AT2G29500	5.825 up	6.54	9.33	6.78
267575_at		AT2G30690	7.124 down	4.54	2.13	4.97
267199_at		AT2G30990	4.168 up	7.55	9.86	7.80
263446_at	ATX1	AT2G31650	1.747 down	8.55	8.45	9.26
263461_at		AT2G31800	4.337 up	8.18	10.43	8.32
267549_at		AT2G32640	1.960 down	5.98	5.89	6.86
267646_at	PHT5	AT2G32830	1.940 down	6.08	5.33	6.29
256725_at		AT2G34070	2.217 up	11.17	11.93	10.78
267428_at		AT2G34840	44.103 down	7.54	1.82	7.28
266546_at		AT2G35270	2.867 up	9.61	10.39	8.87
265846_at	scpl28	AT2G35770	1.912 down	10.13	9.72	10.66
265795_at	scpl26	AT2G35780	9.552 up	7.70	10.78	7.52
263907_at	ABI5	AT2G36270	3.579 down	7.89	6.27	8.11
263921_at		AT2G36460	1.791 down	11.89	11.53	12.37
265224_at		AT2G36710	7.096 up	5.29	7.14	4.31
263840_at		AT2G36885	4.268 up	8.33	10.40	8.31
265959_at		AT2G37240	3.340 down	9.86	8.17	9.91
265962_at		AT2G37460	2.118 down	10.68	10.02	11.10
265965_at		AT2G37500	1.591 down	10.12	9.84	10.51
266200_at		AT2G38920	11.231 down	4.96	3.06	6.55

266183_at	ATPT2	AT2G38940	1.793 down	6.93	6.56	7.40

			Ler over Cvi	log_2	Expression	Level
**Probe Set ID**	**Gene Symbol**	**AGI**	**Fold change**	**HGI2.1**	***Ler***	**Cvi**
266191_at		AT2G39040	40.803 up	6.96	11.91	6.55
266194_at		AT2G39090	1.496 up	8.49	8.95	8.37
266965_at		AT2G39510	7.287 down	8.07	8.59	11.45
267590_at	ATEXPA4	AT2G39700	1.939 down	11.28	10.78	11.74
267349_at		AT2G40010	1.491 down	10.05	9.03	9.60
263831_at	ATFER4	AT2G40300	2.377 up	9.95	9.71	8.47
245098_at	ERS1	AT2G40940	5.218 up	11.08	11.28	8.89
260560_at		AT2G43590	22.258 down	6.84	6.37	10.84
267214_at		AT2G43970	14.413 up	11.55	11.68	7.83
267371_at		AT2G44510	2.417 down	8.63	8.54	9.81
266815_at		AT2G44900	1.696 up	9.72	9.55	8.79
266820_at		AT2G44940	1.666 up	9.50	9.59	8.85
267505_at	CYP76C1	AT2G45560	17.667 down	4.17	2.99	7.13
265448_at	DAG2	AT2G46590	1.612 down	8.26	8.09	8.78
266735_at		AT2G46930	1.593 down	11.07	10.94	11.62
245152_at		AT2G47490	1.412 down	10.43	10.41	10.91
265768_at		AT2G48020	1.738 down	11.01	11.12	11.92
265766_at		AT2G48080	2.359 down	10.44	10.19	11.43
259274_at	ATHB20	AT3G01220	2.349 down	9.43	9.43	10.67
259116_at		AT3G01350	1.380 down	9.12	8.82	9.29
258857_at	scpl25	AT3G02110	2.366 up	9.64	9.59	8.34
259120_at		AT3G02240	228.894 up	9.61	10.29	2.45
257530_at		AT3G03040	3.388 down	8.09	8.12	9.88
259220_at		AT3G03550	2.136 up	9.68	9.85	8.75
259340_at	PYK10	AT3G03870	2.200 up	8.23	8.32	7.18
258816_at		AT3G03960	1.388 down	12.61	12.38	12.86
258810_at		AT3G03970	3.184 down	7.50	7.81	9.48
258912_at		AT3G06460	41.405 up	12.52	12.47	7.09
258510_at		AT3G06600	1.775 up	8.68	8.48	7.65
258631_at	QRT2	AT3G07970	1.708 down	6.89	7.11	7.88
259211_at		AT3G09020	3.183 up	9.10	9.31	7.64
259205_at		AT3G09030	1.856 up	8.65	8.70	7.81
259040_at	ATGSTU8	AT3G09270	4.189 up	13.95	13.88	11.81
257526_s_at		AT3G09330	8.105 down	6.27	5.78	8.79
259033_at		AT3G09405	2.181 down	8.53	8.18	9.30
258939_at		AT3G10020	5.757 up	10.78	11.16	8.64
259154_at		AT3G10260	2.149 up	11.82	12.03	10.93
258758_at		AT3G10810	2.146 up	9.97	9.92	8.82
256433_at	SAG20	AT3G10985	1.997 up	11.62	11.64	10.64
256413_at		AT3G11100	3.191 up	8.47	8.65	6.98
256251_at		AT3G11330	2.513 down	10.62	10.38	11.71
259283_at	MYB65	AT3G11440	1.757 down	8.04	8.00	8.81
258755_at		AT3G11945	2.067 up	11.06	11.34	10.29
256289_s_at	scpl14	AT3G12230	2.514 up	10.09	10.39	9.06
256237_at	DRT100	AT3G12610	1.495 up	12.97	13.18	12.60
257689_at	AtMYB10	AT3G12820	7.283 down	3.26	2.58	5.44
256647_at		AT3G13610	1.355 down	13.54	13.27	13.71
256771_at		AT3G13700	3.837 up	9.98	10.42	8.48
258203_at		AT3G13950	5.875 down	7.60	7.06	9.62
257277_at		AT3G14470	14.145 up	8.30	8.52	4.70
258121_s_at	GGPS3	AT3G14530	4.972 up	8.78	8.64	6.33
257209_at		AT3G14920	6.328 up	9.63	9.74	7.08
258395_at	ANAC055	AT3G15500	1.677 down	6.55	6.33	7.07

258049_at		AT3G16220	14.023 up	8.93	9.25	5.44

Probe Set ID	Gene Symbol	AGI	L*er* over Cvi Fold change	log$_2$ HGI2.1	Expression L*er*	Level Cvi
258048_at	EMB2083	AT3G16290	1.550 down	8.84	8.72	9.36
259379_at		AT3G16350	5.638 up	9.12	10.00	7.51
259384_at		AT3G16450	1.982 up	12.04	12.67	11.68
258424_at		AT3G16750	67.458 down	0.84	0.53	6.61
257888_at		AT3G16990	2.792 up	10.04	9.95	8.47
257874_at		AT3G17110	2.884 up	10.14	10.02	8.49
256852_at	ATRANGAP1	AT3G18610	16.570 down	5.65	4.42	8.47
257022_at	AZF2	AT3G19580	1.582 up	9.58	9.88	9.21
257113_at	CYP705A22	AT3G20130	3.282 up	10.06	10.41	8.70
257675_at	ERF7	AT3G20310	1.320 up	10.37	10.52	10.12
257668_at		AT3G20460	2.081 down	9.36	9.22	10.28
257083_s_at		AT3G20590	5.821 up	8.37	8.85	6.31
256804_at		AT3G20920	1.164 up	12.00	12.04	11.82
256970_at		AT3G21090	3.555 up	6.31	6.25	4.42
258037_at	4CL5	AT3G21230	4.690 up	10.80	10.90	8.67
258170_at		AT3G21600	6.399 down	6.18	5.97	8.65
257949_at	UGT71B1	AT3G21750	2.520 up	9.53	9.46	8.13
256935_at		AT3G22570	137.941 up	12.20	12.36	5.25
258322_at	HMT3	AT3G22740	1.951 up	8.89	9.00	8.03
257766_at	IAA2	AT3G23030	1.736 up	9.56	10.03	9.24
257545_at		AT3G23200	2.098 up	9.20	9.63	8.56
257175_s_at		AT3G23470	112.308 up	9.27	9.17	2.35
258106_at	RNR2A	AT3G23580	1.738 down	8.99	8.92	9.72
257163_at	MYB305	AT3G24310	5.328 down	4.94	4.80	7.21
257868_at	RIN4	AT3G25070	15.437 up	10.59	10.73	6.78
257642_at	BHLH32	AT3G25710	1.513 down	9.26	9.02	9.62
256870_at	CYP71B34	AT3G26300	13.074 up	9.14	9.37	5.66
256875_at	CYP71B37	AT3G26330	15.324 down	6.16	6.05	9.99
256869_at		AT3G26420	11.776 up	9.43	9.47	5.91
256877_at		AT3G26470	4.012 down	10.57	9.94	11.95
257151_at		AT3G27200	13.975 up	11.68	11.71	7.90
257714_at		AT3G27360	6.832 up	10.65	10.58	7.81
257712_at		AT3G27420	1.742 up	9.36	9.70	8.90
258228_at		AT3G27610	5.137 down	6.31	6.50	8.86
256601_s_at	AT14A	AT3G28290	394.924 down	4.98	3.87	12.49
256631_at		AT3G28320	9.109 down	6.68	6.06	9.24
257873_at	PGP17	AT3G28380	47.046 up	6.36	6.34	0.78
256589_at	CYP81D1	AT3G28740	5.983 up	13.57	13.46	10.88
257140_at	MYB30	AT3G28910	2.418 down	7.38	7.53	8.80
258059_at	ATNAC3	AT3G29035	32.599 down	6.82	6.94	11.97
258245_at		AT3G29075	2.504 up	9.96	10.44	9.11
256740_at		AT3G29330	3.211 up	7.14	6.83	5.15
256736_at		AT3G29410	59.583 up	11.50	11.30	5.40
256923_at		AT3G29635	3.020 up	7.59	7.40	5.80
256563_at	RALFL27	AT3G29780	13.473 up	10.80	10.74	6.99
256940_at	QQS	AT3G30720	1174.861 down	3.27	2.58	12.77
257315_at	ERD5	AT3G30775	1.457 down	11.09	11.15	11.69
256605_at		AT3G32940	1.856 down	8.42	8.44	9.34
252703_at		AT3G43740	11.681 down	4.61	5.46	9.01
252662_at	CEF	AT3G44340	3.586 down	9.93	9.88	11.72
252639_at	FAR5	AT3G44550	3.449 down	10.24	9.95	11.73
252551_at		AT3G45880	2.045 up	7.45	7.12	6.09
252563_at	ATEXLA1	AT3G45970	5.588 up	10.65	10.60	8.12

252487_at	UGT76E12	AT3G46660	9.711 up	4.32	6.03	2.75

			L*er* over Cvi	log_2	Expression	Level
**Probe Set ID**	**Gene Symbol**	**AGI**	**Fold change**	**HGI2.1**	**L*er***	**Cvi**
252464_at		AT3G47160	3.823 up	11.29	11.73	9.79
252457_at		AT3G47180	2.209 up	7.70	7.76	6.61
252406_at		AT3G47670	1.761 up	10.64	10.79	9.97
252382_at	ATH5	AT3G47770	16.961 down	4.33	2.67	6.76
252395_at	AHA4	AT3G47950	3.042 down	8.00	7.83	9.43
252367_at	BT2	AT3G48360	16.489 up	9.08	9.80	5.76
252323_at	KING1	AT3G48530	1.496 up	12.25	12.44	11.85
252327_at		AT3G48740	2.584 up	10.72	11.69	10.32
252303_at		AT3G49210	2.168 down	9.47	9.80	10.92
252202_at		AT3G50300	3.223 up	11.20	11.00	9.31
252180_at	KRP2	AT3G50630	1.677 up	10.29	10.14	9.39
252183_at	UGT72E1	AT3G50740	2.079 down	9.09	9.05	10.10
252179_at	GATL2	AT3G50760	1.561 up	8.94	8.84	8.20
252102_at	LTI30	AT3G50970	10.218 up	12.55	12.64	9.28
252095_at		AT3G51000	1.410 down	10.13	10.20	10.69
252114_at		AT3G51450	2.280 up	9.74	9.89	8.70
252070_at		AT3G51680	2.981 down	7.52	6.87	8.44
256671_at	IQD3	AT3G52290	2.593 up	9.60	9.70	8.33
251948_at		AT3G53580	1.207 up	11.64	11.66	11.39
251902_at	PUMP1	AT3G54110	2.250 down	11.98	12.00	13.17
251895_at	ATEP3	AT3G54420	2.846 up	10.58	10.49	8.98
251870_at		AT3G54510	5.446 down	6.49	6.56	9.01
251861_at	BME3	AT3G54810	1.620 down	10.67	10.78	11.47
251835_at		AT3G55180	2.177 up	7.47	7.87	6.74
251797_at	AGF2	AT3G55560	2.066 down	7.89	7.98	9.03
251770_at		AT3G55970	70.066 up	6.69	7.06	0.93
251746_at		AT3G56060	2.087 up	8.24	8.54	7.48
251677_at	BHLH039	AT3G56980	7.421 down	9.64	8.79	11.68
251634_at		AT3G57480	1.768 down	9.27	9.35	10.17
251597_at		AT3G57750	2.334 up	9.51	9.45	8.23
251620_at		AT3G58060	5.494 down	4.99	4.83	7.29
251542_at		AT3G58760	2.591 up	9.49	9.68	8.30
251454_at		AT3G60080	1.253 up	10.82	10.71	10.38
251373_at		AT3G60530	3.146 up	10.93	10.87	9.21
251378_at		AT3G60660	2.142 down	7.47	7.36	8.46
251345_at		AT3G60940	18.952 down	3.57	2.31	6.55
251346_at		AT3G60980	2.093 down	8.77	8.78	9.84
251358_at		AT3G61160	2.322 up	8.48	8.59	7.37
251255_at		AT3G62280	3.146 up	9.94	10.30	8.64
251183_at		AT3G62630	2.864 up	9.50	9.68	8.16
251231_at	ATGSTF13	AT3G62760	3.194 up	9.37	9.18	7.51
251235_at		AT3G62860	2.509 up	10.24	10.25	8.92
251169_at	MARD1	AT3G63210	1.797 up	10.30	10.57	9.73
255702_at	XSP1	AT4G00230	1.922 up	9.26	9.05	8.11
255653_at		AT4G00960	8.650 down	3.20	4.06	7.17
255625_at	GBF2	AT4G01120	13.345 up	10.10	10.22	6.48
255621_at		AT4G01390	6.786 up	10.12	10.17	7.41
255578_at		AT4G01450	4.457 up	11.88	12.01	9.85
255597_at		AT4G01730	3.025 up	9.04	9.14	7.54
255517_at	AtGH9B13	AT4G02290	1.804 up	11.72	11.78	10.93
255473_at		AT4G02450	8.576 down	9.53	9.28	12.38
255407_at		AT4G03480	17.214 down	2.66	2.72	6.82
255377_at		AT4G03500	15.270 down	4.89	5.20	9.14

255385_at		AT4G03610	1.848 down	9.60	9.61	10.49

			L*er* over Cvi	$\log_2$ Expression Level		
**Probe Set ID**	**Gene Symbol**	**AGI**	**Fold change**	**HGI2.1**	**L*er***	**Cvi**
255290_at	ATPC1	AT4G04640	2.596 down	9.08	9.31	10.68
255310_at	ATALN	AT4G04955	2.151 up	11.43	11.74	10.64
255253_at	VPS28-2	AT4G05000	3.023 up	10.11	10.15	8.56
255220_at	UBQ10	AT4G05320	2.833 down	12.26	12.29	13.80
255233_at	MFDX1	AT4G05450	3.346 down	9.06	8.86	10.61
255171_at		AT4G07990	3.175 up	9.27	9.36	7.69
255064_at	EXO	AT4G08950	2.045 up	11.10	11.19	10.16
254998_at		AT4G09760	10.148 down	5.93	5.40	8.74
255013_at		AT4G10000	2.263 up	7.73	7.79	6.61
255010_at		AT4G10050	3.406 up	11.01	10.94	9.17
255812_at	HKT1	AT4G10310	8.053 up	4.51	5.28	2.27
254990_at	CID12	AT4G10610	1.772 up	11.33	11.50	10.67
254945_at		AT4G10940	1.566 down	7.42	7.56	8.20
254952_at	UGE5	AT4G10955	2.061 up	10.39	10.48	9.44
254959_at	UGE5	AT4G10955	2.796 down	8.89	8.40	9.89
254915_s_at		AT4G11310	6.155 up	9.62	9.77	7.15
254844_at		AT4G11790	2.501 up	10.36	10.36	9.03
254804_at		AT4G13010	2.758 up	10.95	10.71	9.25
254798_at		AT4G13050	4.743 down	7.86	7.99	10.24
254749_at		AT4G13130	10.646 up	9.71	9.88	6.47
254766_at	TPS12	AT4G13280	11.704 up	3.71	5.24	1.69
254770_at		AT4G13340	2.293 up	8.02	7.85	6.65
254742_at	IIL1	AT4G13430	1.566 down	12.63	12.57	13.21
254774_at		AT4G13440	3.963 up	9.21	9.25	7.26
254726_at	PRR2	AT4G13660	2.164 down	10.91	10.95	12.06
245595_at		AT4G14170	1.824 down	6.68	6.13	7.00
245566_at		AT4G14610	12.703 up	7.04	7.03	3.36
245543_at		AT4G15260	25.649 down	4.95	5.49	10.17
245561_at	UGT84A4	AT4G15500	3.701 down	9.13	9.36	11.25
245476_at		AT4G16090	6.011 down	3.65	2.11	4.69
245393_at		AT4G16260	3.820 up	11.35	11.39	9.46
245350_at		AT4G16830	2.809 down	9.34	9.40	10.89
245318_at		AT4G16980	12.638 down	5.17	5.99	9.65
245324_at		AT4G17260	10.527 up	10.79	10.64	7.24
245412_at		AT4G17280	2.186 up	11.68	11.57	10.44
245413_at	NS1	AT4G17300	2.217 down	8.24	8.37	9.52
245422_at		AT4G17470	62.857 down	3.20	2.24	8.21
245381_at	MYB39	AT4G17785	3.243 up	7.35	7.14	5.45
254692_at		AT4G17860	17.116 down	4.33	2.73	6.83
254694_at		AT4G17900	2.934 up	11.78	11.80	10.25
254596_at		AT4G18975	5.456 up	9.73	10.06	7.61
254558_at		AT4G19185	1.702 up	10.89	10.81	10.04
254571_at		AT4G19370	7.264 down	6.56	6.18	9.04
254550_at	IRT1	AT4G19690	12.385 down	9.07	8.40	12.03
254536_at		AT4G19720	2.558 down	6.94	7.09	8.45
254537_at		AT4G19730	112.539 down	5.60	4.78	11.60
254540_s_at		AT4G19770	46.959 up	7.77	7.43	1.88
254512_at		AT4G20230	30.758 down	6.02	6.10	11.05
254474_at		AT4G20390	3.063 down	11.76	11.55	13.16
254405_at	PUB8	AT4G21350	3.750 up	9.96	9.72	7.81
254391_at	ENDO3	AT4G21590	6.650 up	8.74	8.82	6.08
254392_at	ENDO5	AT4G21600	12.993 up	11.87	12.30	8.60
254346_at	APG8A	AT4G21980	2.401 up	12.29	12.50	11.24

254361_at		AT4G22212	470.422 up	13.47	13.66	4.79

			L*er* over Cvi	log_2	Expression	Level
Probe Set ID	Gene Symbol	AGI	Fold change	HGI2.1	L*er*	Cvi
254351_at	SOBER1	AT4G22300	2.187 up	10.57	10.61	9.48
254309_s_at		AT4G22390	23.054 down	2.08	2.68	7.20
254326_at		AT4G22610	14.652 up	8.97	10.02	6.15
254276_at		AT4G22820	5.049 up	11.58	11.74	9.40
254299_at	NYE1	AT4G22920	3.250 down	7.70	7.79	9.49
254287_at		AT4G22960	3.844 up	9.81	9.91	7.96
254263_at		AT4G23493	3.982 up	7.93	8.05	6.06
254264_at		AT4G23510	2.704 up	6.35	6.51	5.07
254211_at	SGT1A	AT4G23570	2.587 up	8.30	8.31	6.94
254225_at		AT4G23670	5.110 up	13.66	13.56	11.21
254125_at	TAR2	AT4G24670	2.775 down	7.28	7.12	8.59
254090_at		AT4G25010	7.660 down	6.04	5.27	8.21
254098_at	FSD1	AT4G25100	4.027 down	11.48	11.07	13.08
254093_at	AtMC2	AT4G25110	2.616 down	7.29	6.50	7.89
254107_at		AT4G25220	1.546 down	10.48	10.45	11.08
254020_at	BETA-OHASE 1	AT4G25700	1.645 up	8.92	9.46	8.74
254078_at		AT4G25710	1.360 down	9.52	9.58	10.02
254042_at	XTR6	AT4G25810	3.326 down	7.21	6.47	8.20
253958_at		AT4G26400	1.335 up	9.99	10.13	9.71
253982_at	ATCBL3	AT4G26570	2.282 up	11.44	11.64	10.45
253928_at	PPX1	AT4G26720	1.481 up	10.64	10.57	10.01
253935_at		AT4G26870	3.246 up	11.30	11.20	9.50
253937_at	MAPKKK16	AT4G26890	2.161 up	6.26	6.57	5.46
253950_at		AT4G26910	16.599 up	13.02	13.00	8.95
253914_at		AT4G27400	12.349 down	7.38	7.55	11.18
253872_at	RD26	AT4G27410	2.610 up	11.29	11.75	10.36
253835_at	BGLU9	AT4G27820	5.573 down	5.41	5.24	7.72
253841_at	BGLU10	AT4G27830	34.323 up	9.36	9.30	4.20
253839_at		AT4G27890	9.043 up	9.42	9.46	6.28
253794_at		AT4G28720	2.455 up	9.14	9.40	8.10
253755_at	RPT2a	AT4G29040	1.557 down	12.22	12.30	12.94
253751_at		AT4G29070	1.508 up	9.53	9.81	9.21
253735_at	SNF7.1	AT4G29160	1.275 up	10.19	10.37	10.02
253690_at		AT4G29550	6.480 down	7.08	7.18	9.87
253660_at		AT4G30140	5.731 down	8.21	7.84	10.36
253628_at	XTH18	AT4G30280	9.460 up	11.78	11.96	8.71
253453_at		AT4G31860	1.868 down	10.58	10.61	11.52
253502_at	CYP82C4	AT4G31940	100.899 down	3.59	0.25	6.90
253413_at	ZIP9	AT4G33020	1.681 down	8.02	7.79	8.54
253373_at		AT4G33150	2.578 up	10.26	10.61	9.24
253367_at		AT4G33180	1.617 down	8.52	8.46	9.15
253372_at	PME44	AT4G33220	5.455 down	6.19	5.79	8.24
253344_at		AT4G33550	6.599 up	11.84	12.53	9.80
253312_s_at		AT4G33850	48.213 down	1.78	0.89	6.48
253270_at	CYCD3;1	AT4G34160	2.104 up	8.96	9.05	7.97
253287_at		AT4G34270	2.093 up	11.15	11.10	10.03
253206_at	SQS1	AT4G34640	2.738 up	12.29	12.22	10.77
253253_at		AT4G34750	1.265 up	10.43	10.44	10.10
253195_at		AT4G35420	4.558 down	8.26	8.34	10.52
253104_at		AT4G36010	2.942 down	8.36	8.16	9.72
246270_at		AT4G36500	2.044 up	11.19	11.30	10.27
246272_at	MES9	AT4G37150	2.897 down	9.53	9.25	10.78
253052_at	CYP81H1	AT4G37310	4.708 down	8.58	8.48	10.72

			Ler over Cvi	log2	Expression	Level
**Probe Set ID**	**Gene Symbol**	**AGI**	**Fold change**	**HGI2.1**	***Ler***	**Cvi**
253097_at	CYP81D5	AT4G37320	3.206 down	8.64	8.49	10.17
253053_at		AT4G37470	1.659 up	11.57	11.78	11.05
252979_at		AT4G38225	1.543 down	8.17	8.16	8.78
252997_at	ATEXLA2	AT4G38400	1.844 down	9.22	9.03	9.92
252870_at	AKN2	AT4G39940	1.793 up	11.85	11.62	10.78
251109_at	FER1	AT5G01600	3.611 up	12.88	12.76	10.91
251033_at		AT5G01960	1.348 down	9.80	9.93	10.36
251031_at	OHP	AT5G02120	4.081 up	10.00	10.01	7.98
251023_at		AT5G02170	4.562 up	9.40	10.25	8.06
251008_at		AT5G02710	2.326 down	6.05	5.84	7.05
250931_at		AT5G03200	20.069 down	5.06	4.80	9.13
250920_at		AT5G03390	7.612 down	3.95	3.69	6.62
250952_at	ATIREG2	AT5G03570	2.101 up	9.57	9.84	8.77
250832_at	NAS1	AT5G04950	2.700 down	11.75	11.49	12.92
250704_at		AT5G06265	8.322 up	10.16	10.39	7.33
250689_at		AT5G06610	1.767 up	9.76	9.85	9.03
250611_at	GA20OX3	AT5G07200	4.832 down	7.07	6.97	9.24
250607_at	IPK2a	AT5G07370	1.553 up	10.58	10.85	10.22
250557_at	CPuORF16	AT5G07840	2.591 up	9.27	9.38	8.01
250558_at	TT7	AT5G07990	2.454 up	9.84	9.52	8.23
250547_at		AT5G08100	1.638 up	10.99	10.92	10.20
246055_at	AtAGAL1	AT5G08380	2.895 up	9.82	10.18	8.65
250528_at		AT5G08600	5.886 down	5.43	5.11	7.66
245884_at		AT5G09300	3.346 up	10.46	10.31	8.57
245885_at	EXL4	AT5G09440	2.324 up	12.52	12.71	11.49
250500_at		AT5G09530	1.789 down	13.88	13.66	14.50
250515_at		AT5G09570	3.314 down	8.23	7.33	9.06
250494_at	HAM2	AT5G09740	2.753 up	9.08	9.11	7.65
250474_at	ANNAT7	AT5G10230	2.809 up	10.43	10.59	9.10
250450_at	ATMYB92	AT5G10280	2.066 up	9.43	9.35	8.30
245905_at		AT5G11090	1.574 up	11.64	12.02	11.37
245204_at		AT5G12270	17.643 down	3.13	3.48	7.63
250242_at		AT5G13620	4.533 down	2.97	2.86	5.04
246595_at	FDH	AT5G14780	2.997 up	10.96	11.24	9.66
250152_at		AT5G15120	13.132 down	3.52	2.42	6.14
250136_at	DRM1	AT5G15380	4.256 down	6.26	6.09	8.18
246515_at		AT5G15710	1.833 down	8.68	8.74	9.62
250113_at	FRL1	AT5G16320	1.590 down	8.09	8.23	8.90
250100_at	GLN1;4	AT5G16570	3.216 up	8.98	8.79	7.10
250105_at	RAD4	AT5G16630	2.180 down	7.94	7.98	9.11
246455_at		AT5G16790	4.086 up	4.79	5.03	3.00
250084_at	SDG40	AT5G17240	3.273 down	6.84	6.82	8.53
250091_at		AT5G17340	3.600 up	8.96	8.93	7.08
246427_at	ER-ANT1	AT5G17400	1.437 up	9.73	9.85	9.33
250046_at		AT5G17720	10.641 up	6.44	6.88	3.46
249984_at		AT5G18400	1.830 down	10.47	10.23	11.10
249983_at		AT5G18470	6.449 down	8.11	8.24	10.93
249957_at		AT5G18900	1.763 up	11.69	11.72	10.91
249922_at	AILP1	AT5G19140	2.423 down	10.56	10.54	11.82
249918_at		AT5G19240	5.305 up	12.70	12.31	9.90
249920_at		AT5G19260	1.763 down	9.10	8.62	9.44
245951_at	ASP2	AT5G19550	1.187 down	12.83	12.61	12.86
245945_at	ROPGEF10	AT5G19560	1.757 down	7.10	7.37	8.19
246141_at		AT5G19920	1.369 down	8.28	8.07	8.53

			Ler over Cvi	log$_2$	Expression	Level
Probe Set ID	Gene Symbol	AGI	Fold change	HGI2.1	*Ler*	Cvi
246098_at		AT5G20400	1.761 up	11.42	11.61	10.79
245993_at		AT5G20700	1.510 down	12.63	12.59	13.18
246181_at		AT5G20860	14.370 down	7.00	6.94	10.79
249900_at	emb1211	AT5G22640	1.118 down	9.38	9.39	9.55
249850_at		AT5G23240	2.474 down	4.64	5.30	6.60
249817_at		AT5G23820	14.180 up	13.46	13.82	9.99
249767_at		AT5G24090	3.365 up	11.18	11.36	9.61
249773_at	SQP2	AT5G24140	71.069 down	5.65	5.96	12.12
249737_at		AT5G24480	6.257 down	6.43	6.11	8.76
249738_at		AT5G24510	5.193 down	5.18	4.70	7.07
246957_at		AT5G24670	2.774 down	9.08	9.19	10.66
246944_at		AT5G25450	1.984 up	8.76	8.50	7.52
246901_at		AT5G25630	2.801 down	7.20	7.49	8.98
246909_at		AT5G25770	1.672 up	11.68	11.98	11.24
246831_at	MSS1	AT5G26340	3.142 down	10.85	10.48	12.13
246822_at		AT5G26960	2.603 down	7.81	7.63	9.01
246765_at		AT5G27330	5.105 down	8.05	7.59	9.94
255917_at		AT5G28560	19.946 down	4.80	5.28	9.60
255860_at	AtGUS3	AT5G34940	3.215 up	11.12	11.06	9.38
246651_at		AT5G35170	2.792 up	7.89	8.46	6.98
246610_at		AT5G35400	1.721 down	7.83	7.74	8.52
249675_at		AT5G35940	18.209 up	12.01	12.43	8.24
249690_at		AT5G36210	5.921 up	10.93	10.93	8.36
246617_at		AT5G36270	5.146 up	9.41	9.36	6.99
249636_at	BGLU42	AT5G36890	2.888 down	9.16	9.36	10.90
249576_at		AT5G37690	2.343 down	11.29	11.09	12.32
249584_s_at	NIP4;1	AT5G37810	2.580 down	7.39	6.92	8.28
249595_at		AT5G37930	3.490 up	10.08	10.24	8.43
249601_at		AT5G37980	5.266 up	8.40	8.14	5.74
249567_at		AT5G38020	6.560 down	9.23	9.14	11.86
249466_at	RPL5B	AT5G39740	2.397 up	13.84	13.69	12.43
249437_at		AT5G39990	4.272 down	9.51	9.53	11.62
249388_at		AT5G40090	1.715 up	8.28	8.21	7.43
249357_at		AT5G40490	2.142 down	9.22	9.20	10.30
249331_at	RPL27	AT5G40950	4.337 down	8.17	8.36	10.48
249255_at	CHX18	AT5G41610	2.213 up	7.37	7.51	6.36
249279_at		AT5G41920	1.436 up	6.70	7.17	6.64
249165_at	ATIPK1	AT5G42810	2.900 down	9.31	9.16	10.70
249136_at		AT5G43180	4.346 up	10.73	10.84	8.72
249097_at		AT5G43520	26.931 up	8.31	8.05	3.30
249101_at		AT5G43580	32.022 up	10.52	10.86	5.86
249096_at		AT5G43910	5.725 up	9.47	9.57	7.05
249032_at		AT5G44910	2.000 up	9.41	9.38	8.38
249000_at		AT5G44980	3.399 down	3.29	4.56	6.32
248978_at	AtPP2-A8	AT5G45070	36.577 down	5.80	5.46	10.65
248994_at	RPS4	AT5G45250	106.227 up	7.20	7.62	0.89
248996_at	RRS1	AT5G45260	16.505 up	7.49	7.97	3.93
248944_at		AT5G45500	160.073 down	2.98	3.05	10.37
248945_at		AT5G45510	10.640 down	9.21	9.31	12.73
248819_at		AT5G47050	3.853 down	8.64	8.74	10.69
248796_at		AT5G47180	1.619 up	10.90	11.02	10.32
248793_at	atnudt8	AT5G47240	3.410 up	8.96	9.60	7.83
248790_at	TIP2;3	AT5G47450	5.124 up	11.63	12.54	10.18
248758_at		AT5G47620	1.363 down	11.52	11.45	11.89

			Ler over Cvi	log2	Expression	Level
Probe Set ID	Gene Symbol	AGI	Fold change	HGI2.1	Ler	Cvi
248772_at		AT5G47800	38.917 down	3.30	1.56	6.84
248725_at		AT5G47980	5.645 up	13.73	13.67	11.17
248736_at		AT5G48110	13.792 up	10.77	11.42	7.64
248693_at		AT5G48330	1.923 down	8.62	8.48	9.43
248695_at		AT5G48350	40.940 up	7.64	7.62	2.26
248673_at		AT5G48780	15.503 up	7.72	8.16	4.20
248624_at		AT5G48790	6.504 up	8.34	8.35	5.65
248681_at		AT5G48900	4.841 up	11.64	11.71	9.43
248647_at	SUS2	AT5G49190	2.120 down	5.72	6.03	7.11
248523_s_at	SAE1B	AT5G50580	1.507 up	9.21	9.26	8.67
248407_at		AT5G51500	2.017 down	11.13	10.98	11.99
248336_at		AT5G52420	2.505 up	8.66	8.77	7.44
248366_at		AT5G52510	4.757 up	10.86	11.03	8.77
248327_at		AT5G52750	2.718 down	6.31	5.15	6.59
248245_at		AT5G53190	2.551 up	7.95	8.77	7.42
248267_at	GLT1	AT5G53460	1.699 down	12.92	12.73	13.49
248187_at		AT5G53940	5.182 up	9.70	9.51	7.14
248183_at		AT5G54040	32.134 down	5.39	4.96	9.97
248132_at	SGP1	AT5G54840	5.324 up	10.63	10.82	8.41
248140_at		AT5G54980	4.166 up	10.04	10.47	8.41
248060_at		AT5G55560	2.100 down	7.00	7.27	8.34
248003_at		AT5G56220	2.809 up	10.08	9.92	8.43
247988_at		AT5G56910	3.834 down	6.51	6.08	8.02
247879_at		AT5G57770	1.702 down	8.26	8.16	8.93
247797_at		AT5G58780	19.056 down	3.19	2.14	6.39
247742_at		AT5G58980	3.494 down	7.28	7.44	9.25
247706_at		AT5G59480	4.038 down	8.83	8.99	11.01
247555_at	ECT3	AT5G61020	1.600 down	12.66	12.53	13.21
247577_at		AT5G61290	5.696 down	4.69	5.66	8.17
247519_at	ANAC100	AT5G61430	3.602 down	7.64	7.51	9.36
247497_at	PPAN	AT5G61770	1.829 down	9.82	9.70	10.57
247477_at		AT5G62340	4.075 up	13.25	13.50	11.48
247390_at		AT5G63520	2.111 down	8.17	7.88	8.96
247284_at	OPT4	AT5G64410	4.185 up	9.58	10.07	8.00
247235_at		AT5G64580	2.463 down	7.98	7.67	8.97
247250_at	FAS2	AT5G64630	1.626 up	8.89	8.92	8.22
247213_at	PROPEP1	AT5G64900	3.308 up	8.29	8.51	6.78
247215_at	PROPEP3	AT5G64905	3.672 up	9.08	8.99	7.12
247151_at	bHLH093	AT5G65640	2.207 down	10.13	10.02	11.16
247114_at		AT5G65910	3.891 down	8.33	8.33	10.29
247124_at		AT5G66060	2.150 down	8.11	8.37	9.48
247127_at		AT5G66100	1.416 down	9.56	9.48	9.98
247092_at	ATFOLT1	AT5G66380	1.812 down	9.28	9.19	10.04
247073_at	PSBO1	AT5G66570	2.027 up	10.40	10.75	9.73
259796_at			5.877 down	5.18	5.46	8.01
252754_at			18.212 up	9.07	8.94	4.75
251458_at			31.850 down	2.30	2.16	7.15
245222_at			308.092 down	1.08	1.44	9.71
245032_at			5.485 up	6.92	7.90	5.45
245050_at			4.420 down	7.18	7.49	9.63
245023_at			3.434 down	6.80	7.82	9.60
245021_at			3.285 up	8.83	9.27	7.55
244935_at			7.628 up	11.56	11.77	8.84
244936_at			5.016 up	8.82	9.53	7.20

Table S7-S9

Tables S7-9 are available for download as an Excel file at
http://www.g3journal.org/lookup/suppl/doi:10.1534/g3.111.000331/-/DC1/TableS7.xls.
