# Supplementary material for: A QTL Study for Regions Contributing to Arabidopsis thaliana Root Skewing on Tilted Surfaces
Source: G3 (Bethesda). 2011 Jul 1;1(2):105–15. doi: 10.1534/g3.111.000331 (PMC3276130; doi:10.1534/g3.111.000331)
Supplement: Supporting Information [file supp_1.2.105_FigureS1.pdf]

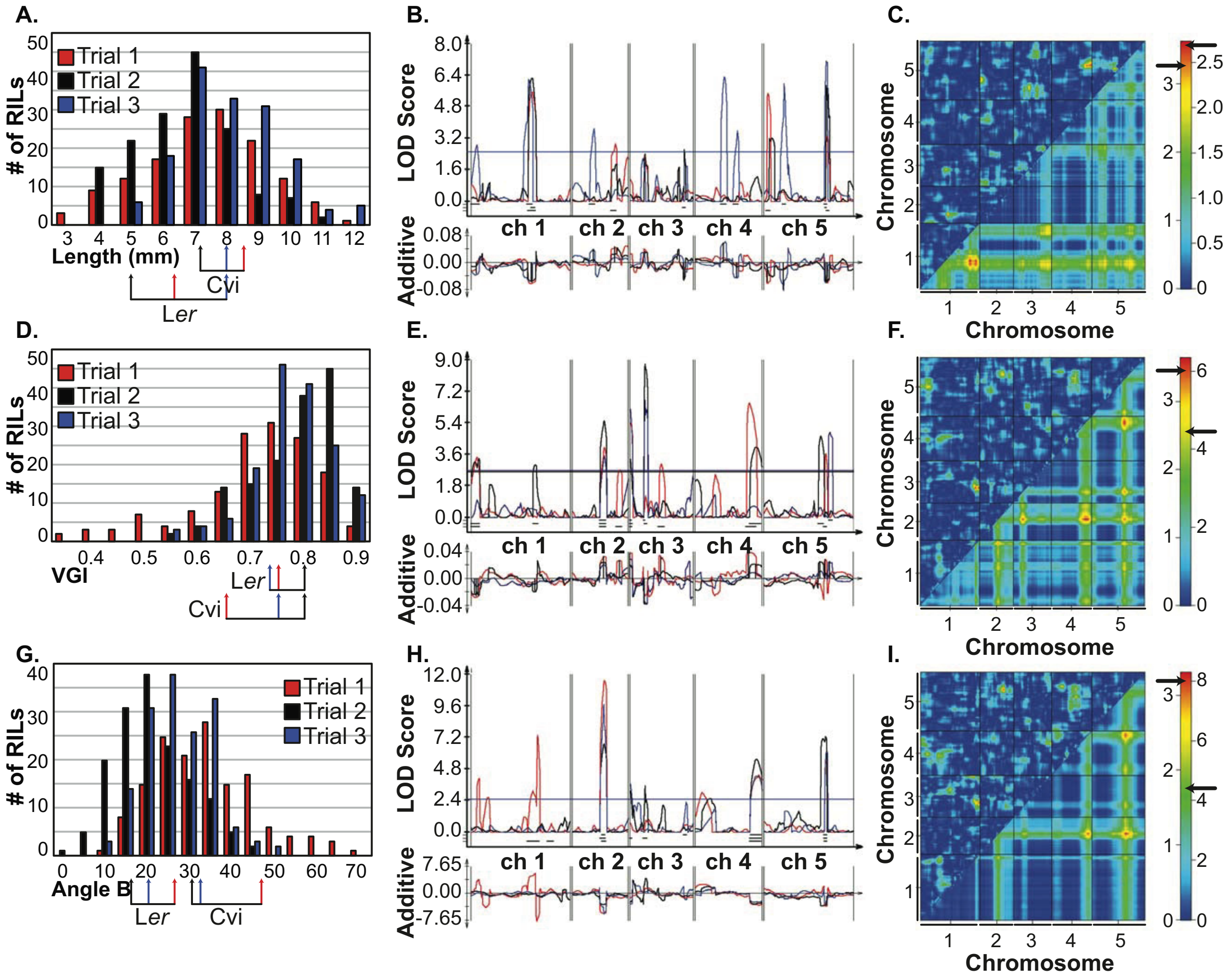

Figure S1 QTL analysis of L, VGI, and angle B in the Cvi/L*er* RIL population. Panels A, D, and G show the histograms of RIL means over the three QTL trials for L, VGI, and angle B, respectively. The color-coded arrows indicate the means for the Cvi and L*er* parents for each trial, "C" is next to the Cvi arrow and "L" is next to those for L*er*. Composite interval mapping (CIM) analysis over three trials for L is shown in panel B, VGI in E, and angle B in H. Trial 1 is red, trial 2 is black, and trial 3 is blue. The top portion of the graph gives the LOD score across the five chromosomes of Arabidopsis. LOD significance thresholds determined by 1000 permutations for each trial are shown as horizontal line across the graph. 2-LOD intervals for significant QTL peaks are black bars beneath the peaks. The bottom graph is an indication of the additive value towards the phenotype of each genomic region with respect to the L*er* allele. Two-dimensional QTL scans are shown in panel C for length, panel F for VGI, and panel I for angle B. Plots are for trial 2. The region of the plot below the diagonal gives the additive QTL model, while the region above the diagonal shows epistatic interaction analysis. Heat map LOD significance thresholds were determined by 1000 permutations. The black arrow on the left LOD scale is the significance threshold for the epistatic portion of the plot, and the arrow on the right side is the threshold for the additive part.
