# Supplementary material for: A QTL Study for Regions Contributing to Arabidopsis thaliana Root Skewing on Tilted Surfaces
Source: G3 (Bethesda). 2011 Jul 1;1(2):105–15. doi: 10.1534/g3.111.000331 (PMC3276130; doi:10.1534/g3.111.000331)
Supplement: Supporting Information [file supp_1.2.105_FigureS2.pdf]

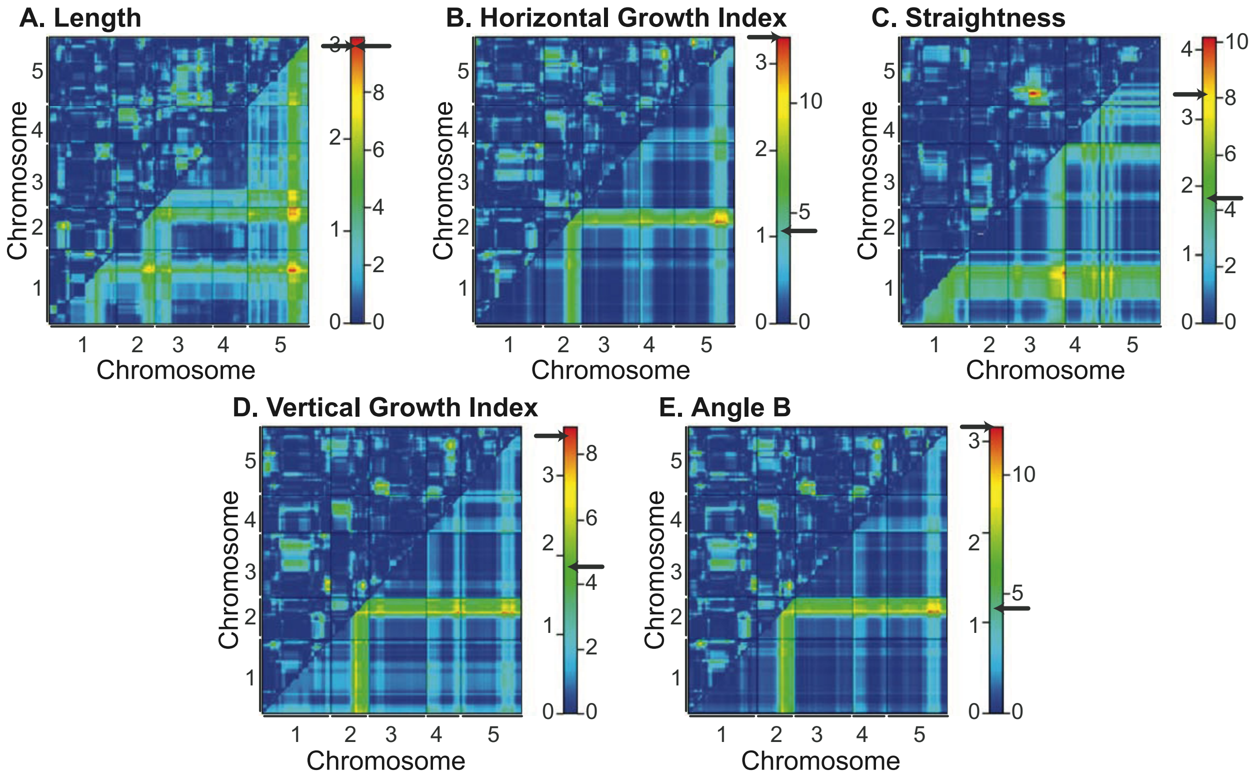

Figure S2 2-D scans for all root growth parameters for trial 1. Two-dimensional QTL scans are shown in panel A for L, B for HGI, C for Lc/L, D for VGI, and E for angle B. The axes represent positions along the five Arabidopsis chromosomes. The region of the plot below the diagonal gives the additive QTL model, while the region above the diagonal shows epistatic interaction analysis. Black arrows indicate significance thresholds with additive on the right and epistatic on the left. Thresholds were determined by 1000 permutation.
