# Supplementary material for: A QTL Study for Regions Contributing to Arabidopsis thaliana Root Skewing on Tilted Surfaces
Source: G3 (Bethesda). 2011 Jul 1;1(2):105–15. doi: 10.1534/g3.111.000331 (PMC3276130; doi:10.1534/g3.111.000331)
Supplement: Supporting Information [file supp_1.2.105_FigureS3.pdf]

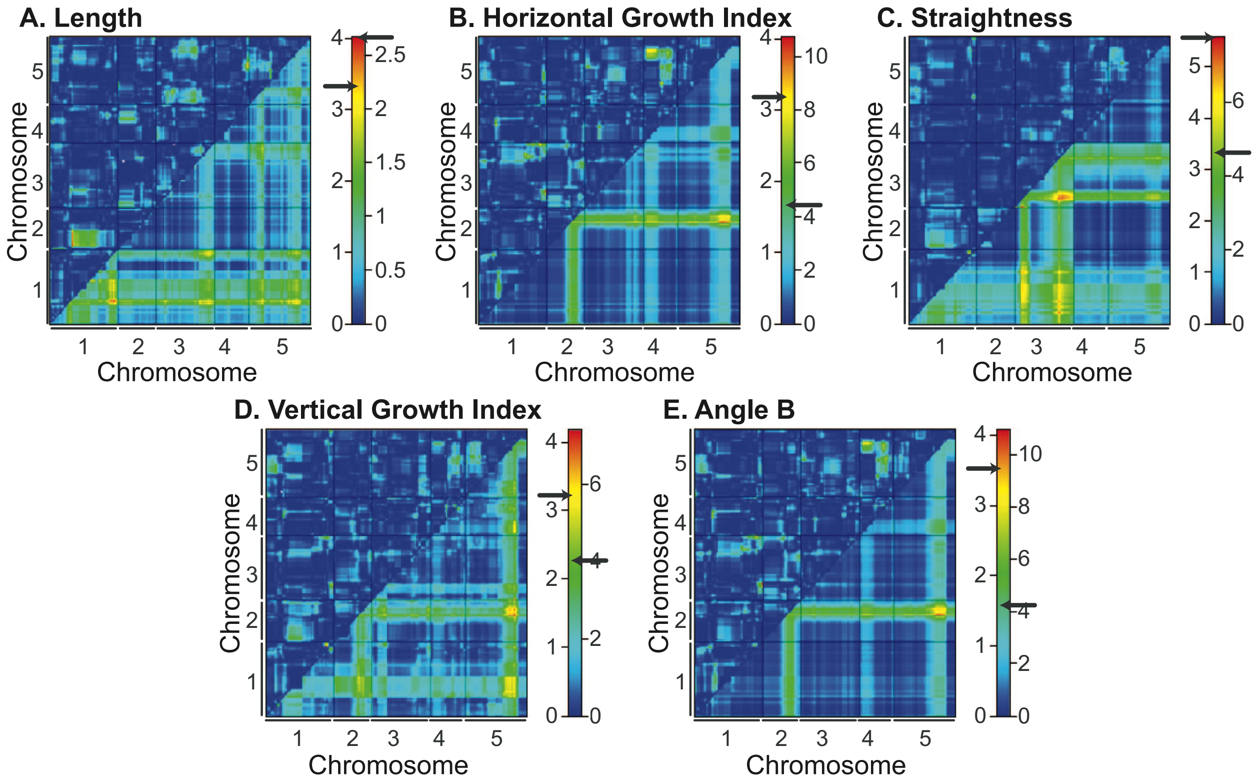

Figure S3 2-D scans for all root growth parameters for trial 3. Two-dimensional QTL scans are shown in panel A for length, B for horizontal growth index (HGI), C for straightness, D for vertical growth index (VGI), and E for angle B. The region of the plot below the diagonal gives the additive QTL model, while the region above the diagonal shows epistatic interaction analysis. Black arrows indicate significance thresholds with additive on the right and epistatic on the left. Thresholds were determined by 1000 permutations.
