# Supplementary material for: A QTL Study for Regions Contributing to Arabidopsis thaliana Root Skewing on Tilted Surfaces
Source: G3 (Bethesda). 2011 Jul 1;1(2):105–15. doi: 10.1534/g3.111.000331 (PMC3276130; doi:10.1534/g3.111.000331)
Supplement: Supporting Information [file supp_1.2.105_FigureS4.pdf]

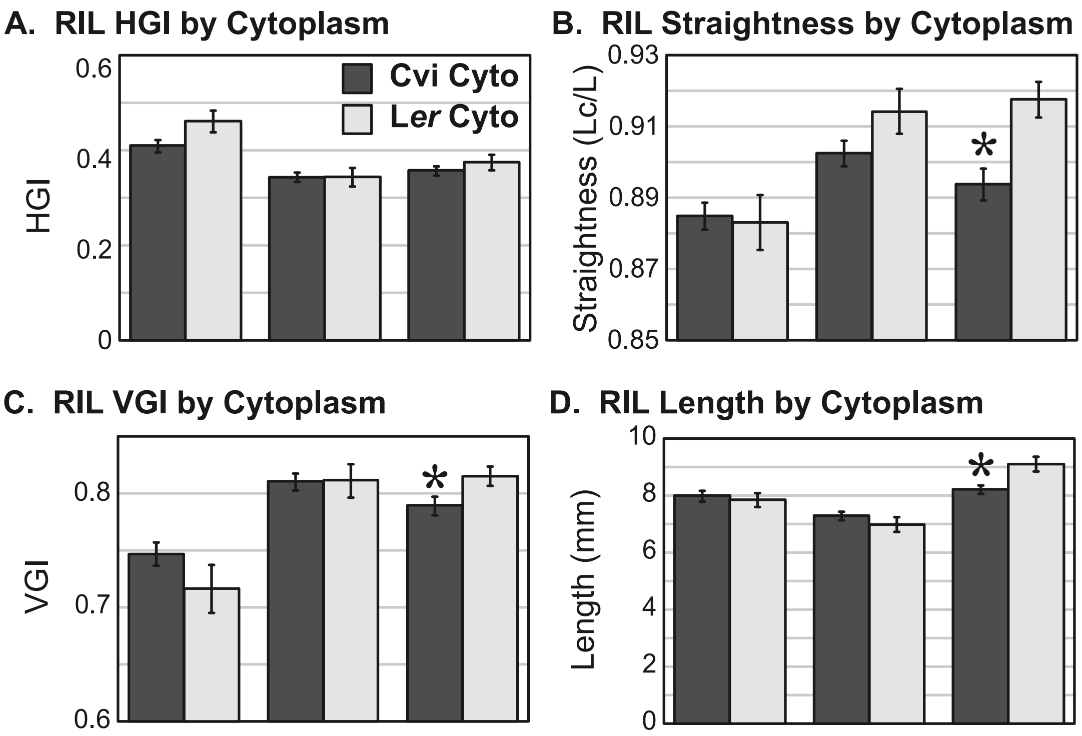

Figure S4 Trait-by-cytoplasm effects for the RILs. For each of three QTL trials, the root morphology measurements are compared based on Cvi and L*er* cytoplasm in the RILs. Traits shown include horizontal growth index (A), straightness (Lc/L), B, vertical growth index (C), and length (D). * indicates p-values less than 0.05 for comparison between the cytoplasm donor. Bars are +/- standard error.
