# Supplementary material for: A QTL Study for Regions Contributing to Arabidopsis thaliana Root Skewing on Tilted Surfaces
Source: G3 (Bethesda). 2011 Jul 1;1(2):105–15. doi: 10.1534/g3.111.000331 (PMC3276130; doi:10.1534/g3.111.000331)
Supplement: Supporting Information [file supp_1.2.105_FigureS5.pdf]

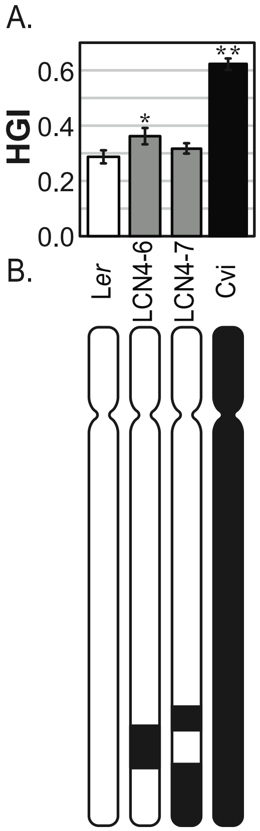

Figure S5 Skewing phenotypes for chromosome 4 NILs. Horizontal growth index (HGI) means for 2 NILs with Cvi introgressions near the end of chromosome 4 are shown in panel A. * indicates a p=0.05 for a skewing difference between L*er* and LCN4-6. ** indicates p<0.0001 between Cvi and L*er* for HGI. At least 30 seedlings were measured in each group. Bars are +/- standard error. Rough schematics of the Cvi introgression (in black) into chromosome 4 are shown in panel B based on the data in Keurentjes *et al.* 2006. LCN4-6 has one Cvi segment and LCN4-7 has two.
