# Supplementary material for: A QTL Study for Regions Contributing to Arabidopsis thaliana Root Skewing on Tilted Surfaces
Source: G3 (Bethesda). 2011 Jul 1;1(2):105–15. doi: 10.1534/g3.111.000331 (PMC3276130; doi:10.1534/g3.111.000331)
Supplement: Supporting Information [file supp_1.2.105_FigureS6.pdf]

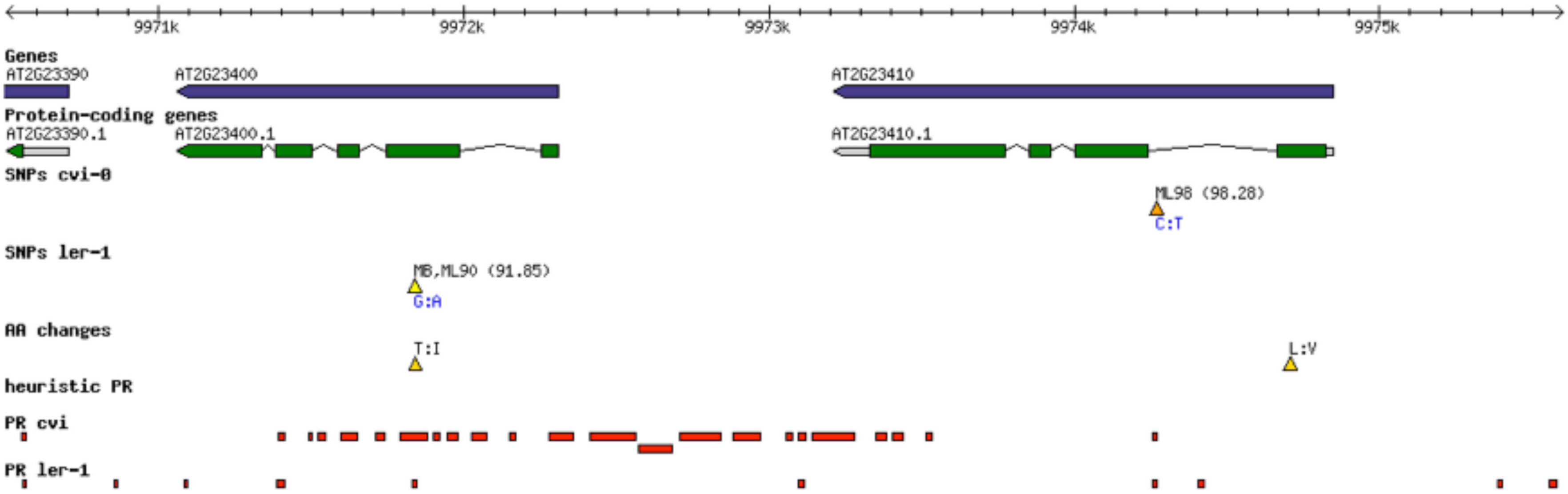

Figure S6 Output from POLYMORPH in GBrowse (http://polymorph.weigelworld.org/; CLARK *et al.* 2007; ZELLER *et al.* 2008) for the regions near At2g23400 and At2g23410 for Cvi and L*er*. Blue boxes represent annotated genes, and protein-coding genes are also displayed in green. Predicted SNPs are shown as triangles, and regions predicted to be polymorphic are highlighted by red bars. Cvi is predicted to be highly polymorphic relative to the Columbia accession over the sequence of At2g23400 and at the 3' end of At2g23410.
