# Supplementary material for: A QTL Study for Regions Contributing to Arabidopsis thaliana Root Skewing on Tilted Surfaces
Source: G3 (Bethesda). 2011 Jul 1;1(2):105–15. doi: 10.1534/g3.111.000331 (PMC3276130; doi:10.1534/g3.111.000331)
Supplement: Supporting Information [file supp_1.2.105_TableS1.pdf]

TABLE S1 Parental and RIL means, RIL mean ranges, and heritabilities over 3 trials for root growth behavior traits.

Trial 1	Cvi mean	Ler mean	RIL range	RIL mean	RIL ANOVA	H^2
Length	8.5	6.43	3.3 to 12.9	7.96	***	0.62
VGI	0.66	0.75	.36 to .91	0.74	***	0.52
HGI	0.62	0.22	.17 to .80	0.42	***	0.47
angle B	47.9°	25.8°	8.1° to 67°	33.9°	***	0.54
Straightness	0.92	0.81	.78 to .96	0.89	***	0.32

Trial 2	Cvi mean	Ler mean	RIL range	RIL mean	RIL ANOVA	H^2
Length	7.2	5.1	4.0 to 11.99	7.2	***	0.49
VGI	0.8	0.81	.56 to .96	0.81	***	0.36
HGI	0.44	0.24	0 to .61	0.34	***	0.29
angle B	28.5°	17.3°	0° to 48.7°	23.6°	***	0.31
Straightness	0.95	0.87	.75 to .98	0.91	***	0.31

Trial 3	Cvi mean	Ler mean	RIL range	RIL mean	RIL ANOVA	H^2
Length	8.5	8.5	4.0 to 13.2	8.4	***	0.45
VGI	0.77	0.75	.42 to .95	0.8	***	0.37
HGI	0.52	0.29	.11 to .65	0.36	***	0.31
angle B	34.2°	20.7°	7.2° to 55.8°	24.7°	***	0.32
Straightness	0.95	0.84	.64 to .97	0.9	***	0.34

*** indicates p-value less than .000001 for variance between RILs versus within a RIL

Broad sense heritability (H^2) determined by variance within RILs divided by variance between RILs
