# Supplementary material for: A QTL Study for Regions Contributing to Arabidopsis thaliana Root Skewing on Tilted Surfaces
Source: G3 (Bethesda). 2011 Jul 1;1(2):105–15. doi: 10.1534/g3.111.000331 (PMC3276130; doi:10.1534/g3.111.000331)
Supplement: Supporting Information [file supp_1.2.105_TableS2.pdf]

Table S2 Significant QTL positions over three trials for root growth behavior traits. Significance levels were determined by 1000 permutations of the data.

Length, Trial 1

Chromosome	Position (cM)	LOD	Phenotypic Effect (R^2)
1	76.5	7.3	0.12
2	69.5	2.7	0.05
3	18.5	5.2	0.08
5	4.5	6.4	0.10
5	78	3.7	0.05

Length, Trial 2

Chromosome	Position (cM)	LOD	Phenotypic Effect (R^2)
1	65	8.1	0.13
3	47.5	2.6	0.04
3	56.5	3.6	0.05
5	6	5.1	0.08
5	7.5	4.9	0.08
5	10.5	5.7	0.10
5	77.5	6.6	0.10
5	106.5	3.3	0.05

Length, Trial 3

Chromosome	Position (cM)	LOD	Phenotypic Effect (R^2)
1	70	2.9	0.05
1	72	3.5	0.05
2	52	4.5	0.08
2	54	4.8	0.08
4	26	3.3	0.05
5	3.5	3.2	0.05
5	8	3.1	0.05
5	78	8.5	0.15

Vertical Growth Index (VGI), Trial 1

Chromosome	Position (cM)	LOD	Phenotypic Effect (R^2)
1	7	3.7	0.05
2	40	4.1	0.06
2	60	2.7	0.04
3	0	5.7	0.09
3	39	3.4	0.05
4	25.5	2.7	0.04
5	78	3.9	0.06

Vertical Growth Index (VGI), Trial 2

Chromosome	Position (cM)	LOD	Phenotypic Effect (R^2)
1	5.5	2.6	0.05
1	10.5	3.1	0.05
2	40	4.9	0.08
3	0	4.1	0.07
3	18.5	7.9	0.13
4	75	3.8	0.07
5	71.5	3.9	0.07

Vertical Growth Index (VGI), Trial 3

Chromosome	Position (cM)	LOD	Phenotypic Effect (R^2)
2	40	3.3	0.06
3	0	3.5	0.06
3	21	3.5	0.07
3	24	4.3	0.08
5	82	6.9	0.12
5	83	7.1	0.13

Horizontal Growth Index (HGI), Trial 1

Chromosome	Position (cM)	LOD	Phenotypic Effect (R^2)
1	84	5.7	0.08
2	40.5	12.5	0.20
3	71	3.1	0.04
4	8	2.6	0.03
4	78	2.8	0.04
5	78	8.3	0.12

Horizontal Growth Index (HGI), Trial 2

Chromosome	Position (cM)	LOD	Phenotypic Effect (R^2)
1	118	2.8	0.04
2	39	10.1	0.19
3	0	4.2	0.07
3	19	4.1	0.06
4	77.5	6.3	0.1
5	79	4.7	0.07

Horizontal Growth Index (HGI), Trial 3

Chromosome	Position (cM)	LOD	Phenotypic Effect (R^2)
2	40	10.2	0.16
3	0	3.4	0.05
3	11.5	3.2	0.05
3	13.5	3	0.05
3	70	3.8	0.06
4	25	3.2	0.05
4	80	2.8	0.05
5	78.5	6.3	0.1
5	80.5	6.2	0.1

Angle B, Trial 1

Chromosome	Position (cM)	LOD	Phenotypic Effect (R^2)
1	90.5	4.4	0.08
2	40.5	3.9	0.06
2	62.5	3	0.05
4	8	2.7	0.04
4	73	4.4	0.07
5	78	5.5	0.08

Angle B, Trial 2

Chromosome	Position (cM)	LOD	Phenotypic Effect (R^2)
1	119	4.8	0.09
2	40	7.2	0.12
3	0	4.5	0.07
3	19	5	0.08
4	76	5.3	0.09

Angle B, Trial 3

Chromosome	Position (cM)	LOD	Phenotypic Effect (R^2)
2	39.5	10.9	0.2
3	0	4	0.06
3	21.5	3	0.05
3	23	4.2	0.06
4	76.5	3.2	0.05
4	80.5	3.3	0.05
5	79	6.3	0.09
5	80.5	6.3	0.1

Straightness, Trial 1

Chromosome	Position (cM)	LOD	Phenotypic Effect (R^2)
1	7	4.7	0.07
1	68	7.4	0.13
3	0	5.5	0.08
3	18.5	7.2	0.11
3	79.5	6.8	0.10
5	24.5	4.7	0.07

Straightness, Trial 2

Chromosome	Position (cM)	LOD	Phenotypic Effect (R^2)
1	12	4.5	0.07
1	72	5.6	0.08
3	17.5	6.7	0.11
3	70	3.8	0.06
5	25	2.7	0.04
5	70	3.5	0.06

Straightness, Trial 3

Chromosome	Position (cM)	LOD	Phenotypic Effect (R^2)
3	0	4.1	0.08
3	18.5	2.7	0.05
3	30	3.2	0.06
3	66.5	3.1	0.06
3	69.5	3.4	0.06
