# Supplementary material for: A QTL Study for Regions Contributing to Arabidopsis thaliana Root Skewing on Tilted Surfaces
Source: G3 (Bethesda). 2011 Jul 1;1(2):105–15. doi: 10.1534/g3.111.000331 (PMC3276130; doi:10.1534/g3.111.000331)
Supplement: Supporting Information [file supp_1.2.105_TableS3.pdf]

Table S3 Significance values for DOG 17 root trait means compared to L*er*

	Length	VGI	HGI	Straightness	Angle B
DOG 17-1	**		**		**
DOG 17-2	**	*			*
DOG 17 -3					*
DOG 17 -4		**	**		**

* = p<0.05

**=p<0.001
