# Supplementary material for: A QTL Study for Regions Contributing to Arabidopsis thaliana Root Skewing on Tilted Surfaces
Source: G3 (Bethesda). 2011 Jul 1;1(2):105–15. doi: 10.1534/g3.111.000331 (PMC3276130; doi:10.1534/g3.111.000331)
Supplement: Supporting Information [file supp_1.2.105_TableS4.pdf]

Table S4 Primers used for chromosome 2 fine mapping, indel, SSLP, and CAPs

CHROMOSOME 2 INDEL PRIMERS

Forward Primer	Reverse Primer	TAIR build 9 Forward Primer Start (bp)	Cvi product size	*Ler* product size	Source
GAGAGTCGCAAAAACCTTGG	AGAAGAAACCGCGACGAATA	2943966	1291	542	a
CTAGCAAGACGCATCAAGGG	CTAATGACGACTTCGAGAAAGTT	5797945	247	202	a
CAATGTTTTGTGGGCTGTTG	CGCAATTGTTAATGTGGTTCC	6370922	529	481	a
GGTGTCAAAGTTTCCGTCACACAC	ACTACAGAGGCTTACGAGGCCAAA	7342710	200	155	a
CTGGGGTGGAAAGAATCTGA	CACCGGTGTAACctttagcc	7715949	136	100	b
cgtctccgataactagtggatg	TCTTCGGTTATTAGGAGTGTG	8139986	160	172	b
TTGTGAAGGGTTTGAGTCCA	GCGCCGGAGAAGACTATGTA	8997379	384	423	a
GCCATGTTTGAAGGCATTGT	GATCTGCAGACGAGGGAAAG	9267216	717	550	a
aaccctatcaacgggcagaag	TATGGACATAGTCTCGGCATG	9347455	388	335	b
AACCAGTTATGGCCTTTCCTT	TTCCACGTAAGCCAAATTCC	9290093	550	422	a
GTTAGCTCACTCCAAATCGAGAAG	GTTTCATCGTACTTGCTTGGGAGG	9347566	353	300	a
AGCCAAATGCCAAATTCAAG	CTGGAATTCAGTTGGCCATT	9659301	332	284	a
ccttatatgaaaacagagagggtca	GGACAAGACCCGACTTCTGA	10020562	410	~380	c
TGTTGGTAGGTCGCCCTCT	GCCATTGCAAAAGAACATCA	10026853	437	470	a
cgacttcttactcttcaccgaca	tgttgtgtcattgtgaacatgg	10046432	~450	499	c
cggtcggatctgaatacattg	gccacatagggatagttgcag	10199101	200	177	a
aagtttcaacccatcaaataaataga	tatattggcgttgccgacac	10430711	200	179	a
ggaattttccttcgtgttttca	cagtttgtgtacgggatgaat	10560001	400	361	a
GATTTACATATGCCAATCCG	CTTCCGTCTCTGTCTCAAACTG	10866073	251	226	a
TCGACCGGAAAACAAGATTC	acaaattcaggtgttagctgttt	10934438	400	347	a
tcgtactgaccgacctttga	tggaatcgtgtttctcctga	11194426	166	154	a
TTCTCACCGAAATCGATGCT	TCAGTGCATAGTGAGTAAgattgga	11221229	391	~360	c
GCTTCTCTGGACACACCTCA	GCGAATCTCGTGGTTACTCC	11232714	195	179	a
TGAATATGCAGGATACGACTGTG	TGCTAATTTATACGCATCTGTCTC	11446331	381	~360	c
cactttatgttttgggtggtaca	cgttccaagataatgtaagtgct	12404780	226	210	a

caacggcctttcaagagttc	ttagcggcagtggttatcaa	13237730	187	172	a
tggcaaatatgtagccagaaa	acacatccccacatgccta	13498360	109	100	a
tccattcaatatttcgtctcg	acacggtttagcaacgatct	13786899	114	104	a
TGATTTCTCCTTACCTGAAAAAGG	TTCAACTGTTGTCATTTCATCAT	14118356	466	419	a
CTTTATGTGCCGAAGTTCAAA	CATGGTTAGGCCCGTTAAGA	15293730	475	444	a
cactagtaagatcttgttgatg	gatggtcattttgagattcatag	15579317	244	172	a
ATCCCTGAACTTCACATCTGCTGC	TCACTCCTCTTGGTTTCAGTAGTGG	16465929	200	161	a
GCCATTGTTCGATTTGCTTTGGTTTGG	GGGCTTGCAACATTGTAAATAGGC	16474309	318	293	a
tgttgagtgttcaaacggtga	aacgttcgtggagtatacttttct	17061306	127	113	a
ttctgaaattagtatttgtgccgta	tcaatcattaaaaccgtttcctg	17421092	208	192	a
CGTCACCGCAATTTCACTAA	TGATCCTGATTTGGGTCTCC	18307407	630	574	a
TTGTGCATCGCTTTCAGCATTCGG	AGTTGTAATATGGCAACCGCAGCC	18891919	360	317	a

CHROMOSOME 2 CAPs PRIMERS

**Forward Primer**	**Reverse Primer**	**TAIR build 9 Forward Primer Start (bp)**	**Cvi product size**	**L*er* product size**	**Source**	**Enzyme**
TGGGTGTTCACAGGATCTCA	AGCTCTGACGCAACCTCATT	9957327	~280 and 520	807	d	BanI
agccacgaaccaaaaattatc	cttttggctctccagctgat	11039713	176 and 374	550	a	SspI
CATTGTTCTCTTCCAGGAAACTT	AAACAAAGCATGACTCTGATCAAT	11148407	122 and 224	346	a	AluI
TTACAAGCATCATGGAGACAGAg	TCCAAGTAGTGGCGGTCTTT	11153101	216 and 384	600	a	BsbRI
AGCTTCACCTCCAACTTCCA	CGTGAAGAAGCCTCTACAGTGA	11171336	617	~180 and 440	d	Hpy188I
AAACTCCACAAAAGCACAAACA	TCCAAACCCAAATCTTGACC	11683970	940	~350 and 590	d	Tsp45I
GCTTGGAGCAGCCACATTAC	GCGCTAATTATTTCGGGACA	13082931	~180 and 150	334	d	BbvI
Cctgcaagaatcgagatcca	ATCAGGGCTTGTGGTGGTAG	13748944	~400 and 550	946	d	BsmAI

a: Source is CEREON/Monsanto Col/L*er* polymorphisms

b: Source is Julin Maloof

c: Source is POLYMORPH Bur/Tsu PRs

d: Source is TAIR polymorphism bank
