# Supplementary material for: A QTL Study for Regions Contributing to Arabidopsis thaliana Root Skewing on Tilted Surfaces
Source: G3 (Bethesda). 2011 Jul 1;1(2):105–15. doi: 10.1534/g3.111.000331 (PMC3276130; doi:10.1534/g3.111.000331)
Supplement: Supporting Information [file supp_1.2.105_TableS5.pdf]

**Table S5 Chromosome 2 Cvi introgression mapping in Cvi/L*er* NILs**

| NIL Name | L*er* to Cvi beakpoint unknown (bps) | Known Cvi introgression (bps) | Cvi to L*er* breakpoint unknown (bps) |
|---|---|---|---|
| LCN2-4 | 0 - 2943966 | 2943966 - 8997379 | 8997379 - 9347566 |
| LCN2-6 | 6370922 - 7342710 | 7342710 - 8997379 | 8997379 - 9347566 |
| LCN2-7 | 6370922 - 7342710 | 7342710 - 16465929 | 16465929 - 17421092 |
| HGI2.3 | 6370922 - 7342710 | 7342710 - 8139986 | 8139986 - 8997379 |
| HGI2.4 | 7342710 - 7715949 | 7715949 - 9347566 | 9347566 - 9659301 |
| HGI2.5 | 7342710 - 7715949 | 7715949 - 9347566 | 9347566 - 9659301 |
| HGI2.2 | 7715949 - 8139986 | 8139986 - 16465929 | 16465929 - 17421092 |
| HGI2.1 | 7715949 - 8139986 | 8139986 - 16465929 | 16465929 - 17421092 |
| LCN2-8 | 10934438 - 11194426 | 11194426 - 13082931 | 13082931 - 13748944 |
| LCN2-9 | 10934438 - 11194426 | 11194426 - 15293730 | 15293730 - 15579317 |
