# Supplementary material for: A QTL Study for Regions Contributing to Arabidopsis thaliana Root Skewing on Tilted Surfaces
Source: G3 (Bethesda). 2011 Jul 1;1(2):105–15. doi: 10.1534/g3.111.000331 (PMC3276130; doi:10.1534/g3.111.000331)
Supplement: Supporting Information [file supp_1.2.105_TableS6.pdf]

Table S6   699 Probe Sets at 95% Confidence for Differential Expression between Cvi and L*er*

Probe Set ID	Gene Symbol	AGI	L*er* over Cvi Fold change	log_2 Expression Level HGI2.1	L*er*	Cvi
261577_at		AT1G01080	2.177 down	7.18	7.24	8.37
261581_at	CIPK9	AT1G01140	1.798 down	8.15	8.25	9.09
261537_at		AT1G01800	2.266 up	12.90	12.94	11.76
259416_at		AT1G02305	3.198 up	12.73	12.84	11.16
259415_at		AT1G02330	1.486 up	9.32	9.54	8.97
262107_at		AT1G02750	3.100 up	9.66	9.61	7.98
262118_at	BGLU11	AT1G02850	2.236 up	11.03	11.13	9.97
264354_s_at		AT1G03200	6.361 up	6.69	7.52	4.85
264830_at		AT1G03710	116.089 down	4.05	4.26	11.12
263182_at		AT1G05575	2.903 up	9.07	8.88	7.34
262625_at		AT1G06440	2.098 down	6.80	6.45	7.52
262616_at		AT1G06620	2.387 up	10.31	11.04	9.79
262637_at		AT1G06640	4.746 up	12.82	12.82	10.57
256053_at	UGT71C3	AT1G07260	2.358 up	8.78	8.90	7.66
261785_at		AT1G08230	26.732 up	6.19	6.67	1.93
261720_at	HDA08	AT1G08460	1.327 down	8.89	9.08	9.49
261696_at		AT1G08470	1.549 down	10.60	10.44	11.07
264802_at	SYP111	AT1G08560	1.968 up	11.38	11.43	10.45
264811_at		AT1G08640	1.724 down	10.04	9.90	10.69
264665_at		AT1G09660	1.306 down	10.82	10.72	11.10
260480_at	SNRK2.4	AT1G10940	2.997 up	10.12	10.28	8.70
262452_at		AT1G11210	2.180 up	8.22	8.86	7.74
262456_at	STP1	AT1G11260	1.834 up	12.25	12.52	11.64
261846_at		AT1G11540	2.733 up	9.13	9.40	7.95
262811_at		AT1G11700	1.778 down	8.61	8.45	9.28
260967_at		AT1G12230	1.530 down	11.23	11.30	11.91
259536_s_at	ATCOAB	AT1G12350	1.644 up	10.23	10.14	9.42
259529_at		AT1G12400	11.508 down	6.31	5.27	8.80
261201_at		AT1G12850	2.478 down	9.73	9.88	11.19
259366_at	AOC4	AT1G13280	2.365 up	12.22	12.49	11.25
256134_at	AAPT1	AT1G13560	1.149 up	12.39	12.25	12.05
256070_at		AT1G13730	1.652 down	11.88	11.58	12.30
262655_s_at		AT1G14185	2.510 down	7.52	7.48	8.80
261474_at		AT1G14540	2.776 up	10.53	10.23	8.76
259502_at		AT1G15670	2.343 down	10.76	10.94	12.17
262712_at	ATRDH2	AT1G16460	3.634 up	10.63	10.55	8.69
261087_at		AT1G17350	4.193 up	10.79	10.84	8.77
256073_at	E12A11	AT1G18100	5.518 down	5.36	5.69	8.15
259481_at	GLP4	AT1G18970	4.347 up	10.51	10.35	8.23
259478_at		AT1G18980	8.351 up	13.19	13.05	9.98
256012_at	FMO1	AT1G19250	21.180 up	7.90	8.50	4.09
261226_at	ATEXPA11	AT1G20190	2.584 up	10.90	11.10	9.73
261459_at		AT1G21100	5.048 down	9.34	9.86	12.20
261450_s_at		AT1G21110	21.725 up	11.01	11.14	6.70
261448_at		AT1G21140	2.233 up	9.30	9.07	7.91
260902_at		AT1G21440	2.225 down	9.92	9.99	11.15
255968_at		AT1G22270	2.521 up	11.00	10.79	9.45
255942_at	AtUGT85A2	AT1G22360	9.762 up	12.00	12.06	8.77
261927_at		AT1G22500	1.793 up	10.93	11.17	10.33
264198_at		AT1G22800	1.637 down	9.61	9.39	10.10
264202_at		AT1G22810	2.759 up	6.78	6.63	5.17

			Ler over Cvi	log$_2$	Expression	Level
**Probe Set ID**	**Gene Symbol**	**AGI**	**Fold change**	**HGI2.1**	***Ler***	**Cvi**
264773_at		AT1G22900	10.017 up	7.12	6.78	3.46
257412_at		AT1G22980	4.590 down	6.38	6.27	8.47
264893_at		AT1G23140	3.076 up	9.06	8.96	7.34
263032_at		AT1G23850	10.596 up	8.09	8.61	5.21
263033_s_at		AT1G23950	2.184 down	11.36	11.24	12.37
263028_at		AT1G24030	4.125 down	6.91	6.81	8.85
265017_at		AT1G24390	16.790 up	6.88	6.43	2.36
245650_at		AT1G24735	5.543 down	6.91	6.76	9.23
245651_s_at		AT1G24793	2.539 up	11.52	11.65	10.30
245646_x_at		AT1G24851	6.189 up	8.41	8.54	5.91
255731_at	RCN1	AT1G25490	1.364 down	12.68	12.56	13.01
245873_at	CIB5	AT1G26260	1.800 down	7.56	7.53	8.38
265003_at		AT1G26970	3.914 up	8.52	8.75	6.78
264987_at		AT1G27030	2.518 up	13.10	13.28	11.94
264977_at		AT1G27090	1.546 down	11.27	11.10	11.73
264979_s_at		AT1G27170	3.144 down	6.60	6.51	8.16
262319_s_at		AT1G27540	13.053 up	13.07	13.30	9.59
259606_at	MAP65-8	AT1G27920	4.194 up	8.45	8.67	6.60
255995_at	RKF1	AT1G29750	1.871 down	7.91	7.57	8.47
257418_at		AT1G30850	2.033 down	8.14	8.22	9.25
265130_at		AT1G30890	2.257 down	10.50	10.49	11.66
265155_at		AT1G30990	14.989 down	7.07	7.25	11.16
263701_at		AT1G31160	4.205 up	9.45	9.46	7.39
263695_at		AT1G31220	2.436 down	8.24	7.77	9.05
256492_at		AT1G31490	7.800 down	4.86	4.26	7.23
256493_at		AT1G31600	2.812 down	8.54	8.66	10.15
246259_at		AT1G31830	1.671 down	9.53	9.53	10.27
245792_at	PRR1	AT1G32100	1.760 up	12.02	12.11	11.29
252522_at		AT1G32337	9.993 down	1.00	-1.12	2.20
260652_at		AT1G32360	1.953 up	9.06	9.23	8.27
261239_at		AT1G32930	2.560 up	9.09	9.25	7.89
261616_at	ANAC014	AT1G33060	1.505 down	7.89	7.88	8.47
261596_at		AT1G33080	3.077 down	6.68	6.86	8.48
256531_at		AT1G33320	5.602 down	6.20	6.04	8.53
261985_at		AT1G33750	203.229 up	10.40	10.36	2.70
262564_at		AT1G34330	5.544 up	9.00	9.22	6.75
262561_at		AT1G34340	2.101 down	8.28	8.22	9.29
261156_s_at		AT1G34490	4.827 up	9.03	8.94	6.66
261157_at		AT1G34510	434.134 up	10.92	11.19	2.42
259576_at		AT1G35330	3.325 down	8.76	8.63	10.37
259577_at		AT1G35340	3.640 up	8.82	8.91	7.05
263192_at	ACC1	AT1G36160	6.885 up	10.41	10.46	7.68
251905_at	GAPB	AT1G42970	4.804 down	8.11	8.44	10.71
264415_at	RAP2.6	AT1G43160	7.020 up	9.23	9.20	6.39
264421_at	ARP1	AT1G43170	3.019 down	13.30	13.32	14.91
262725_at		AT1G43580	1.587 down	10.18	10.17	10.84
245737_at		AT1G44160	4.260 up	7.78	8.12	6.03
245245_at	hemb2	AT1G44318	3.964 up	10.85	10.25	8.26
261335_at		AT1G44800	3.921 down	12.19	11.68	13.65
245803_at	RD21	AT1G47128	1.405 down	13.30	13.36	13.85
260730_at	mtLPD1	AT1G48030	1.617 down	13.35	13.18	13.87
262416_at		AT1G49390	5.889 up	8.09	8.38	5.82
261603_at	ATRBP47A	AT1G49600	1.317 down	12.84	12.71	13.11
261609_at		AT1G49740	2.095 up	9.57	9.76	8.69

262421_at		AT1G50290	7.695 up	9.10	9.47	6.53

			**L*er* over Cvi**	**log_2**	**Expression**	**Level**
**Probe Set ID**	**Gene Symbol**	**AGI**	**Fold change**	**HGI2.1**	**L*er***	**Cvi**
261878_at	CYP705A25	AT1G50560	2.776 up	11.22	11.38	9.90
265136_at		AT1G51270	13.588 up	4.68	5.64	1.88
265142_at	DABB1	AT1G51360	2.965 down	8.23	7.96	9.53
260486_at		AT1G51550	1.441 down	7.05	6.75	7.28
256178_s_at	ILL5	AT1G51760	1.301 up	9.80	10.06	9.68
259841_at		AT1G52200	3.238 up	12.37	12.47	10.77
261342_at		AT1G52950	342.777 down	0.88	1.25	9.67
260617_at		AT1G53345	3.064 down	6.42	6.23	7.85
262198_at	ATPME2	AT1G53830	2.322 up	10.56	10.75	9.54
263156_at		AT1G54030	1.727 up	12.75	13.05	12.26
263145_at	ATEXO70D2	AT1G54090	4.950 up	9.67	9.91	7.60
263007_at		AT1G54260	10.597 up	9.05	9.11	5.71
263008_at	RPP27	AT1G54470	4.438 up	3.54	4.11	1.96
264191_at		AT1G54730	2.044 up	8.35	8.59	7.56
264185_at		AT1G54780	2.083 up	8.91	9.41	8.35
256352_at	ATPRP1	AT1G54970	2.782 up	11.43	11.62	10.14
265123_at		AT1G55440	5.128 up	7.95	7.68	5.32
265070_at	BCDH BETA1	AT1G55510	2.949 up	9.33	9.31	7.75
264536_at	PRA1.G1	AT1G55640	9.851 up	8.06	7.59	4.29
262095_at		AT1G56090	2.337 up	9.14	9.14	7.92
245676_at		AT1G56670	2.120 down	6.43	6.11	7.19
245657_at		AT1G56720	3.110 up	9.17	9.53	7.89
246410_at		AT1G57760	2.106 up	9.04	9.75	8.68
246399_at		AT1G58110	1.649 up	9.97	10.00	9.28
246394_at		AT1G58160	6.614 down	6.10	6.41	9.13
262897_at	CCB4	AT1G59840	2.062 down	6.23	6.18	7.23
262908_at	AT-E1 ALPHA	AT1G59900	22.342 down	7.34	7.06	11.54
262913_at		AT1G59960	2.580 down	9.06	8.96	10.33
264217_at		AT1G60190	2.365 up	7.29	7.82	6.57
264920_at	ECHID	AT1G60550	3.290 up	9.04	9.27	7.56
264932_at		AT1G61240	3.712 down	7.88	7.86	9.75
264881_s_at		AT1G61270	2.383 up	6.54	6.86	5.60
264754_at		AT1G61400	2.502 down	6.65	5.91	7.23
265035_at		AT1G61620	1.303 up	10.54	10.38	10.00
264398_at		AT1G61730	5.140 down	9.09	8.95	11.31
264731_at		AT1G62150	2.549 down	8.36	8.31	9.66
265114_at	LRX2	AT1G62440	2.331 down	5.94	6.00	7.22
265120_at	KAS III	AT1G62640	2.469 down	10.91	11.11	12.42
259692_at		AT1G63080	2.409 up	7.13	6.84	5.57
262340_at	ANAC027	AT1G64100	3.413 up	9.28	9.30	7.52
262353_at		AT1G64210	3.505 down	5.90	5.80	7.61
261957_at	ATMGL	AT1G64660	3.056 up	7.84	7.39	5.78
262865_at	CYP89A7	AT1G64930	3.021 down	8.40	7.84	9.43
266155_at	CYP89A5	AT1G64940	107.667 down	4.68	4.59	11.34
262875_at	G-TMT	AT1G64970	1.709 down	8.11	7.72	8.49
264158_at	PTAC4	AT1G65260	2.017 up	10.05	10.33	9.32
264159_at		AT1G65270	1.582 up	11.56	11.73	11.07
262933_at	ATPAO4	AT1G65840	2.109 down	12.37	12.30	13.38
257583_at	PMI2	AT1G66480	1.830 up	9.44	9.63	8.75
256376_s_at	PXMT1	AT1G66690	2.947 down	7.55	7.41	8.97
264970_at		AT1G67280	2.029 up	10.75	10.83	9.81
264229_at		AT1G67480	1.399 down	11.77	12.01	12.50
245215_at	ATFXG1	AT1G67830	6.045 down	5.28	5.93	8.53

260401_at		AT1G69840	2.472 down	9.07	8.92	10.23

			Ler over Cvi	log_2	Expression	Level
**Probe Set ID**	**Gene Symbol**	**AGI**	**Fold change**	**HGI2.1**	***Ler***	**Cvi**
260411_at		AT1G69890	3.363 up	7.37	7.63	5.88
264720_at		AT1G70080	20.814 up	10.13	10.26	5.88
260207_at		AT1G70730	4.658 up	10.90	10.84	8.62
259900_at	CSN5B	AT1G71230	1.591 down	8.57	8.45	9.12
259941_s_at		AT1G71280	4.430 down	5.38	5.23	7.38
260172_s_at		AT1G71920	1.944 down	10.37	10.27	11.23
259911_at	CAD1	AT1G72680	1.398 down	11.28	11.30	11.78
262362_at		AT1G72840	3.957 up	7.54	7.42	5.44
260097_at	AtOCT1	AT1G73220	72.674 down	4.30	3.71	9.89
260101_at		AT1G73260	12.186 up	12.65	12.57	8.96
245736_at	ATDR4	AT1G73330	5.139 down	10.66	10.91	13.28
245731_at	MKK9	AT1G73500	8.916 up	9.90	10.07	6.91
260072_at		AT1G73650	1.603 up	11.29	11.38	10.70
260234_at		AT1G74460	1.433 down	12.12	11.96	12.48
262171_at	TIFY10B	AT1G74950	1.544 up	10.81	11.08	10.46
262686_at		AT1G75990	1.343 down	10.97	10.94	11.37
259977_at		AT1G76590	3.106 up	8.15	8.29	6.65
259979_at		AT1G76600	1.612 up	10.50	10.20	9.51
259866_at		AT1G76640	14.251 up	6.37	7.44	3.61
264957_at	SKP2B	AT1G77000	3.710 up	9.68	10.06	8.17
259705_at	anac032	AT1G77450	2.008 up	12.09	12.13	11.12
260058_at		AT1G78100	1.494 down	10.13	10.10	10.67
260806_at		AT1G78260	2.915 down	9.38	9.41	10.95
264100_at	LUP1	AT1G78970	48.544 down	2.49	1.91	7.51
264131_at		AT1G79150	1.780 down	10.86	10.88	11.72
264144_at	AtMC6	AT1G79320	2.195 down	7.38	7.40	8.54
257475_at		AT1G80880	1.846 down	7.35	7.48	8.36
261901_at	J8	AT1G80920	3.384 up	12.06	12.38	10.62
265741_at		AT2G01320	1.703 down	7.52	7.74	8.51
266202_at		AT2G02400	5.737 up	10.92	10.82	8.30
267220_at	ISPD	AT2G02500	2.882 down	8.60	8.56	10.09
267240_at		AT2G02680	13.843 up	8.65	8.82	5.03
267479_at		AT2G02690	30.411 down	3.16	2.45	7.38
266743_at	RNS1	AT2G02990	3.002 down	7.97	8.48	10.06
265718_at	WRKY3	AT2G03340	2.016 up	8.80	9.29	8.28
265707_at		AT2G03390	1.787 up	9.51	9.55	8.72
264032_at	GEK1	AT2G03800	1.381 down	9.66	9.51	9.97
265651_at		AT2G13900	4.285 down	3.07	4.25	6.35
263276_at	CYP705A13	AT2G14100	23.289 up	10.20	10.61	6.07
265575_at	PIP	AT2G14260	6.049 down	9.30	9.17	11.76
266588_at	AGP9	AT2G14890	4.332 up	13.72	13.65	11.53
265486_at		AT2G15560	18.358 down	5.08	6.84	11.04
263098_at		AT2G16005	8.224 up	13.77	13.99	10.95
263607_at		AT2G16270	2.492 up	7.44	8.01	6.70
266525_at	MEE15	AT2G16970	9.058 down	7.74	7.97	11.15
263417_at		AT2G17180	1.435 down	2.25	1.51	2.03
263419_at		AT2G17220	3.201 down	8.40	8.44	10.12
264877_at	CYP51G2	AT2G17330	3.707 up	9.40	9.12	7.23
264904_s_at	NTRA	AT2G17420	3.011 down	11.65	11.51	13.11
264594_at	ATSERAT3;1	AT2G17640	1.946 down	7.79	8.19	9.15
264790_at	ATHK1	AT2G17820	2.272 down	8.79	8.67	9.86
265817_at	HIS1-3	AT2G18050	7.628 up	7.37	7.94	5.01
265929_s_at		AT2G18560	1.861 down	8.36	8.17	9.07

266693_at	MIOX2	AT2G19800	2.283 up	7.95	8.87	7.67

			**L*er* over Cvi**	**log$_2$**	**Expression**	**Level**
**Probe Set ID**	**Gene Symbol**	**AGI**	**Fold change**	**HGI2.1**	**L*er***	**Cvi**
266680_s_at		AT2G19850	314.826 down	3.77	2.02	10.32
265586_at	PR-1-LIKE	AT2G19990	13.491 down	4.77	3.02	6.77
265395_at	SRF1	AT2G20850	3.022 down	8.52	7.49	9.08
265438_at		AT2G20970	5.989 down	3.29	0.60	3.18
257432_at		AT2G21850	7.834 up	6.81	10.35	7.38
267263_at		AT2G23110	1.628 down	5.19	4.72	5.42
267131_at		AT2G23400	28.735 up	1.40	6.63	1.78
267137_at	ACPT	AT2G23410	4.785 up	8.92	10.51	8.25
267286_at		AT2G23640	4.172 down	5.00	3.97	6.04
267288_at		AT2G23680	1.961 down	8.79	7.91	8.88
267297_at		AT2G23780	1.349 up	10.26	10.55	10.12
265689_at		AT2G24310	1.581 up	7.20	7.87	7.21
263788_at		AT2G24580	2.695 down	10.28	9.52	10.95
263537_at	COL3	AT2G24790	2.768 up	9.79	11.13	9.66
263526_at		AT2G24830	2.655 up	7.95	9.22	7.81
263539_at	TAT3	AT2G24850	5.468 up	6.34	7.98	5.53
265897_at	MOT1	AT2G25680	5.404 down	6.84	5.45	7.88
265898_at		AT2G25690	2.051 down	8.58	7.81	8.85
266647_at		AT2G25870	1.632 up	7.59	8.37	7.66
266895_at		AT2G26040	3.323 up	8.42	8.92	7.19
267374_at		AT2G26230	2.331 up	10.11	11.56	10.34
267606_at	KCS11	AT2G26640	2.522 up	9.09	10.80	9.47
266310_at	FUS12	AT2G26990	2.368 up	9.24	10.89	9.64
250612_s_at	ARR13	AT2G27070	6.329 up	3.96	5.38	2.72
263083_at	PAP12	AT2G27190	5.856 up	10.02	12.18	9.63
265666_at		AT2G27440	9.117 down	7.25	4.87	8.06
266209_at	ATC	AT2G27550	2.496 up	10.18	10.61	9.29
266262_at		AT2G27590	2.593 up	9.34	10.39	9.01
266260_at	SKD1	AT2G27600	3.318 up	10.08	11.84	10.11
263438_at		AT2G28660	3.579 up	7.07	9.02	7.18
266276_at	TRI	AT2G29330	5.580 up	7.56	10.14	7.66
266296_at	ATGSTU7	AT2G29420	2.951 up	11.16	13.19	11.63
266271_at	ATGSTU6	AT2G29440	4.074 up	9.88	11.21	9.18
266294_at		AT2G29500	5.825 up	6.54	9.33	6.78
267575_at		AT2G30690	7.124 down	4.54	2.13	4.97
267199_at		AT2G30990	4.168 up	7.55	9.86	7.80
263446_at	ATX1	AT2G31650	1.747 down	8.55	8.45	9.26
263461_at		AT2G31800	4.337 up	8.18	10.43	8.32
267549_at		AT2G32640	1.960 down	5.98	5.89	6.86
267646_at	PHT5	AT2G32830	1.940 down	6.08	5.33	6.29
256725_at		AT2G34070	2.217 up	11.17	11.93	10.78
267428_at		AT2G34840	44.103 down	7.54	1.82	7.28
266546_at		AT2G35270	2.867 up	9.61	10.39	8.87
265846_at	scpl28	AT2G35770	1.912 down	10.13	9.72	10.66
265795_at	scpl26	AT2G35780	9.552 up	7.70	10.78	7.52
263907_at	ABI5	AT2G36270	3.579 down	7.89	6.27	8.11
263921_at		AT2G36460	1.791 down	11.89	11.53	12.37
265224_at		AT2G36710	7.096 up	5.29	7.14	4.31
263840_at		AT2G36885	4.268 up	8.33	10.40	8.31
265959_at		AT2G37240	3.340 down	9.86	8.17	9.91
265962_at		AT2G37460	2.118 down	10.68	10.02	11.10
265965_at		AT2G37500	1.591 down	10.12	9.84	10.51
266200_at		AT2G38920	11.231 down	4.96	3.06	6.55

266183_at	ATPT2	AT2G38940	1.793 down	6.93	6.56	7.40
			Ler over Cvi	log2	Expression	Level
**Probe Set ID**	**Gene Symbol**	**AGI**	**Fold change**	**HGI2.1**	***Ler***	**Cvi**
266191_at		AT2G39040	40.803 up	6.96	11.91	6.55
266194_at		AT2G39090	1.496 up	8.49	8.95	8.37
266965_at		AT2G39510	7.287 down	8.07	8.59	11.45
267590_at	ATEXPA4	AT2G39700	1.939 down	11.28	10.78	11.74
267349_at		AT2G40010	1.491 down	10.05	9.03	9.60
263831_at	ATFER4	AT2G40300	2.377 up	9.95	9.71	8.47
245098_at	ERS1	AT2G40940	5.218 up	11.08	11.28	8.89
260560_at		AT2G43590	22.258 down	6.84	6.37	10.84
267214_at		AT2G43970	14.413 up	11.55	11.68	7.83
267371_at		AT2G44510	2.417 down	8.63	8.54	9.81
266815_at		AT2G44900	1.696 up	9.72	9.55	8.79
266820_at		AT2G44940	1.666 up	9.50	9.59	8.85
267505_at	CYP76C1	AT2G45560	17.667 down	4.17	2.99	7.13
265448_at	DAG2	AT2G46590	1.612 down	8.26	8.09	8.78
266735_at		AT2G46930	1.593 down	11.07	10.94	11.62
245152_at		AT2G47490	1.412 down	10.43	10.41	10.91
265768_at		AT2G48020	1.738 down	11.01	11.12	11.92
265766_at		AT2G48080	2.359 down	10.44	10.19	11.43
259274_at	ATHB20	AT3G01220	2.349 down	9.43	9.43	10.67
259116_at		AT3G01350	1.380 down	9.12	8.82	9.29
258857_at	scpl25	AT3G02110	2.366 up	9.64	9.59	8.34
259120_at		AT3G02240	228.894 up	9.61	10.29	2.45
257530_at		AT3G03040	3.388 down	8.09	8.12	9.88
259220_at		AT3G03550	2.136 up	9.68	9.85	8.75
259340_at	PYK10	AT3G03870	2.200 up	8.23	8.32	7.18
258816_at		AT3G03960	1.388 down	12.61	12.38	12.86
258810_at		AT3G03970	3.184 down	7.50	7.81	9.48
258912_at		AT3G06460	41.405 up	12.52	12.47	7.09
258510_at		AT3G06600	1.775 up	8.68	8.48	7.65
258631_at	QRT2	AT3G07970	1.708 down	6.89	7.11	7.88
259211_at		AT3G09020	3.183 up	9.10	9.31	7.64
259205_at		AT3G09030	1.856 up	8.65	8.70	7.81
259040_at	ATGSTU8	AT3G09270	4.189 up	13.95	13.88	11.81
257526_s_at		AT3G09330	8.105 down	6.27	5.78	8.79
259033_at		AT3G09405	2.181 down	8.53	8.18	9.30
258939_at		AT3G10020	5.757 up	10.78	11.16	8.64
259154_at		AT3G10260	2.149 up	11.82	12.03	10.93
258758_at		AT3G10810	2.146 up	9.97	9.92	8.82
256433_at	SAG20	AT3G10985	1.997 up	11.62	11.64	10.64
256413_at		AT3G11100	3.191 up	8.47	8.65	6.98
256251_at		AT3G11330	2.513 down	10.62	10.38	11.71
259283_at	MYB65	AT3G11440	1.757 down	8.04	8.00	8.81
258755_at		AT3G11945	2.067 up	11.06	11.34	10.29
256289_s_at	scpl14	AT3G12230	2.514 up	10.09	10.39	9.06
256237_at	DRT100	AT3G12610	1.495 up	12.97	13.18	12.60
257689_at	AtMYB10	AT3G12820	7.283 down	3.26	2.58	5.44
256647_at		AT3G13610	1.355 down	13.54	13.27	13.71
256771_at		AT3G13700	3.837 up	9.98	10.42	8.48
258203_at		AT3G13950	5.875 down	7.60	7.06	9.62
257277_at		AT3G14470	14.145 up	8.30	8.52	4.70
258121_s_at	GGPS3	AT3G14530	4.972 up	8.78	8.64	6.33
257209_at		AT3G14920	6.328 up	9.63	9.74	7.08
258395_at	ANAC055	AT3G15500	1.677 down	6.55	6.33	7.07

Probe Set ID	Gene Symbol	AGI	*Ler* over Cvi Fold change	log_2 Expression Level HGI2.1	*Ler*	Cvi
258049_at		AT3G16220	14.023 up	8.93	9.25	5.44
258048_at	EMB2083	AT3G16290	1.550 down	8.84	8.72	9.36
259379_at		AT3G16350	5.638 up	9.12	10.00	7.51
259384_at		AT3G16450	1.982 up	12.04	12.67	11.68
258424_at		AT3G16750	67.458 down	0.84	0.53	6.61
257888_at		AT3G16990	2.792 up	10.04	9.95	8.47
257874_at		AT3G17110	2.884 up	10.14	10.02	8.49
256852_at	ATRANGAP1	AT3G18610	16.570 down	5.65	4.42	8.47
257022_at	AZF2	AT3G19580	1.582 up	9.58	9.88	9.21
257113_at	CYP705A22	AT3G20130	3.282 up	10.06	10.41	8.70
257675_at	ERF7	AT3G20310	1.320 up	10.37	10.52	10.12
257668_at		AT3G20460	2.081 down	9.36	9.22	10.28
257083_s_at		AT3G20590	5.821 up	8.37	8.85	6.31
256804_at		AT3G20920	1.164 up	12.00	12.04	11.82
256970_at		AT3G21090	3.555 up	6.31	6.25	4.42
258037_at	4CL5	AT3G21230	4.690 up	10.80	10.90	8.67
258170_at		AT3G21600	6.399 down	6.18	5.97	8.65
257949_at	UGT71B1	AT3G21750	2.520 up	9.53	9.46	8.13
256935_at		AT3G22570	137.941 up	12.20	12.36	5.25
258322_at	HMT3	AT3G22740	1.951 up	8.89	9.00	8.03
257766_at	IAA2	AT3G23030	1.736 up	9.56	10.03	9.24
257545_at		AT3G23200	2.098 up	9.20	9.63	8.56
257175_s_at		AT3G23470	112.308 up	9.27	9.17	2.35
258106_at	RNR2A	AT3G23580	1.738 down	8.99	8.92	9.72
257163_at	MYB305	AT3G24310	5.328 down	4.94	4.80	7.21
257868_at	RIN4	AT3G25070	15.437 up	10.59	10.73	6.78
257642_at	BHLH32	AT3G25710	1.513 down	9.26	9.02	9.62
256870_at	CYP71B34	AT3G26300	13.074 up	9.14	9.37	5.66
256875_at	CYP71B37	AT3G26330	15.324 down	6.16	6.05	9.99
256869_at		AT3G26420	11.776 up	9.43	9.47	5.91
256877_at		AT3G26470	4.012 down	10.57	9.94	11.95
257151_at		AT3G27200	13.975 up	11.68	11.71	7.90
257714_at		AT3G27360	6.832 up	10.65	10.58	7.81
257712_at		AT3G27420	1.742 up	9.36	9.70	8.90
258228_at		AT3G27610	5.137 down	6.31	6.50	8.86
256601_s_at	AT14A	AT3G28290	394.924 down	4.98	3.87	12.49
256631_at		AT3G28320	9.109 down	6.68	6.06	9.24
257873_at	PGP17	AT3G28380	47.046 up	6.36	6.34	0.78
256589_at	CYP81D1	AT3G28740	5.983 up	13.57	13.46	10.88
257140_at	MYB30	AT3G28910	2.418 down	7.38	7.53	8.80
258059_at	ATNAC3	AT3G29035	32.599 down	6.82	6.94	11.97
258245_at		AT3G29075	2.504 up	9.96	10.44	9.11
256740_at		AT3G29330	3.211 up	7.14	6.83	5.15
256736_at		AT3G29410	59.583 up	11.50	11.30	5.40
256923_at		AT3G29635	3.020 up	7.59	7.40	5.80
256563_at	RALFL27	AT3G29780	13.473 up	10.80	10.74	6.99
256940_at	QQS	AT3G30720	1174.861 down	3.27	2.58	12.77
257315_at	ERD5	AT3G30775	1.457 down	11.09	11.15	11.69
256605_at		AT3G32940	1.856 down	8.42	8.44	9.34
252703_at		AT3G43740	11.681 down	4.61	5.46	9.01
252662_at	CEF	AT3G44340	3.586 down	9.93	9.88	11.72
252639_at	FAR5	AT3G44550	3.449 down	10.24	9.95	11.73
252551_at		AT3G45880	2.045 up	7.45	7.12	6.09
252563_at	ATEXLA1	AT3G45970	5.588 up	10.65	10.60	8.12

252487_at	UGT76E12	AT3G46660	9.711 up	4.32	6.03	2.75
			L*er* over Cvi	log_2	Expression	Level
**Probe Set ID**	**Gene Symbol**	**AGI**	**Fold change**	**HGI2.1**	**L*er***	**Cvi**
252464_at		AT3G47160	3.823 up	11.29	11.73	9.79
252457_at		AT3G47180	2.209 up	7.70	7.76	6.61
252406_at		AT3G47670	1.761 up	10.64	10.79	9.97
252382_at	ATH5	AT3G47770	16.961 down	4.33	2.67	6.76
252395_at	AHA4	AT3G47950	3.042 down	8.00	7.83	9.43
252367_at	BT2	AT3G48360	16.489 up	9.08	9.80	5.76
252323_at	KING1	AT3G48530	1.496 up	12.25	12.44	11.85
252327_at		AT3G48740	2.584 up	10.72	11.69	10.32
252303_at		AT3G49210	2.168 down	9.47	9.80	10.92
252202_at		AT3G50300	3.223 up	11.20	11.00	9.31
252180_at	KRP2	AT3G50630	1.677 up	10.29	10.14	9.39
252183_at	UGT72E1	AT3G50740	2.079 down	9.09	9.05	10.10
252179_at	GATL2	AT3G50760	1.561 up	8.94	8.84	8.20
252102_at	LTI30	AT3G50970	10.218 up	12.55	12.64	9.28
252095_at		AT3G51000	1.410 down	10.13	10.20	10.69
252114_at		AT3G51450	2.280 up	9.74	9.89	8.70
252070_at		AT3G51680	2.981 down	7.52	6.87	8.44
256671_at	IQD3	AT3G52290	2.593 up	9.60	9.70	8.33
251948_at		AT3G53580	1.207 up	11.64	11.66	11.39
251902_at	PUMP1	AT3G54110	2.250 down	11.98	12.00	13.17
251895_at	ATEP3	AT3G54420	2.846 up	10.58	10.49	8.98
251870_at		AT3G54510	5.446 down	6.49	6.56	9.01
251861_at	BME3	AT3G54810	1.620 down	10.67	10.78	11.47
251835_at		AT3G55180	2.177 up	7.47	7.87	6.74
251797_at	AGF2	AT3G55560	2.066 down	7.89	7.98	9.03
251770_at		AT3G55970	70.066 up	6.69	7.06	0.93
251746_at		AT3G56060	2.087 up	8.24	8.54	7.48
251677_at	BHLH039	AT3G56980	7.421 down	9.64	8.79	11.68
251634_at		AT3G57480	1.768 down	9.27	9.35	10.17
251597_at		AT3G57750	2.334 up	9.51	9.45	8.23
251620_at		AT3G58060	5.494 down	4.99	4.83	7.29
251542_at		AT3G58760	2.591 up	9.49	9.68	8.30
251454_at		AT3G60080	1.253 up	10.82	10.71	10.38
251373_at		AT3G60530	3.146 up	10.93	10.87	9.21
251378_at		AT3G60660	2.142 down	7.47	7.36	8.46
251345_at		AT3G60940	18.952 down	3.57	2.31	6.55
251346_at		AT3G60980	2.093 down	8.77	8.78	9.84
251358_at		AT3G61160	2.322 up	8.48	8.59	7.37
251255_at		AT3G62280	3.146 up	9.94	10.30	8.64
251183_at		AT3G62630	2.864 up	9.50	9.68	8.16
251231_at	ATGSTF13	AT3G62760	3.194 up	9.37	9.18	7.51
251235_at		AT3G62860	2.509 up	10.24	10.25	8.92
251169_at	MARD1	AT3G63210	1.797 up	10.30	10.57	9.73
255702_at	XSP1	AT4G00230	1.922 up	9.26	9.05	8.11
255653_at		AT4G00960	8.650 down	3.20	4.06	7.17
255625_at	GBF2	AT4G01120	13.345 up	10.10	10.22	6.48
255621_at		AT4G01390	6.786 up	10.12	10.17	7.41
255578_at		AT4G01450	4.457 up	11.88	12.01	9.85
255597_at		AT4G01730	3.025 up	9.04	9.14	7.54
255517_at	AtGH9B13	AT4G02290	1.804 up	11.72	11.78	10.93
255473_at		AT4G02450	8.576 down	9.53	9.28	12.38
255407_at		AT4G03480	17.214 down	2.66	2.72	6.82
255377_at		AT4G03500	15.270 down	4.89	5.20	9.14

255385_at		AT4G03610	1.848 down	9.60	9.61	10.49

			Ler over Cvi	log_2	Expression	Level
**Probe Set ID**	**Gene Symbol**	**AGI**	**Fold change**	**HGI2.1**	***Ler***	**Cvi**
255290_at	ATPC1	AT4G04640	2.596 down	9.08	9.31	10.68
255310_at	ATALN	AT4G04955	2.151 up	11.43	11.74	10.64
255253_at	VPS28-2	AT4G05000	3.023 up	10.11	10.15	8.56
255220_at	UBQ10	AT4G05320	2.833 down	12.26	12.29	13.80
255233_at	MFDX1	AT4G05450	3.346 down	9.06	8.86	10.61
255171_at		AT4G07990	3.175 up	9.27	9.36	7.69
255064_at	EXO	AT4G08950	2.045 up	11.10	11.19	10.16
254998_at		AT4G09760	10.148 down	5.93	5.40	8.74
255013_at		AT4G10000	2.263 up	7.73	7.79	6.61
255010_at		AT4G10050	3.406 up	11.01	10.94	9.17
255812_at	HKT1	AT4G10310	8.053 up	4.51	5.28	2.27
254990_at	CID12	AT4G10610	1.772 up	11.33	11.50	10.67
254945_at		AT4G10940	1.566 down	7.42	7.56	8.20
254952_at	UGE5	AT4G10955	2.061 up	10.39	10.48	9.44
254959_at	UGE5	AT4G10955	2.796 down	8.89	8.40	9.89
254915_s_at		AT4G11310	6.155 up	9.62	9.77	7.15
254844_at		AT4G11790	2.501 up	10.36	10.36	9.03
254804_at		AT4G13010	2.758 up	10.95	10.71	9.25
254798_at		AT4G13050	4.743 down	7.86	7.99	10.24
254749_at		AT4G13130	10.646 up	9.71	9.88	6.47
254766_at	TPS12	AT4G13280	11.704 up	3.71	5.24	1.69
254770_at		AT4G13340	2.293 up	8.02	7.85	6.65
254742_at	IIL1	AT4G13430	1.566 down	12.63	12.57	13.21
254774_at		AT4G13440	3.963 up	9.21	9.25	7.26
254726_at	PRR2	AT4G13660	2.164 down	10.91	10.95	12.06
245595_at		AT4G14170	1.824 down	6.68	6.13	7.00
245566_at		AT4G14610	12.703 up	7.04	7.03	3.36
245543_at		AT4G15260	25.649 down	4.95	5.49	10.17
245561_at	UGT84A4	AT4G15500	3.701 down	9.13	9.36	11.25
245476_at		AT4G16090	6.011 down	3.65	2.11	4.69
245393_at		AT4G16260	3.820 up	11.35	11.39	9.46
245350_at		AT4G16830	2.809 down	9.34	9.40	10.89
245318_at		AT4G16980	12.638 down	5.17	5.99	9.65
245324_at		AT4G17260	10.527 up	10.79	10.64	7.24
245412_at		AT4G17280	2.186 up	11.68	11.57	10.44
245413_at	NS1	AT4G17300	2.217 down	8.24	8.37	9.52
245422_at		AT4G17470	62.857 down	3.20	2.24	8.21
245381_at	MYB39	AT4G17785	3.243 up	7.35	7.14	5.45
254692_at		AT4G17860	17.116 down	4.33	2.73	6.83
254694_at		AT4G17900	2.934 up	11.78	11.80	10.25
254596_at		AT4G18975	5.456 up	9.73	10.06	7.61
254558_at		AT4G19185	1.702 up	10.89	10.81	10.04
254571_at		AT4G19370	7.264 down	6.56	6.18	9.04
254550_at	IRT1	AT4G19690	12.385 down	9.07	8.40	12.03
254536_at		AT4G19720	2.558 down	6.94	7.09	8.45
254537_at		AT4G19730	112.539 down	5.60	4.78	11.60
254540_s_at		AT4G19770	46.959 up	7.77	7.43	1.88
254512_at		AT4G20230	30.758 down	6.02	6.10	11.05
254474_at		AT4G20390	3.063 down	11.76	11.55	13.16
254405_at	PUB8	AT4G21350	3.750 up	9.96	9.72	7.81
254391_at	ENDO3	AT4G21590	6.650 up	8.74	8.82	6.08
254392_at	ENDO5	AT4G21600	12.993 up	11.87	12.30	8.60
254346_at	APG8A	AT4G21980	2.401 up	12.29	12.50	11.24

254361_at		AT4G22212	470.422 up	13.47	13.66	4.79
			L*er* over Cvi	log_2	Expression	Level
Probe Set ID	Gene Symbol	AGI	Fold change	HGI2.1	L*er*	Cvi
254351_at	SOBER1	AT4G22300	2.187 up	10.57	10.61	9.48
254309_s_at		AT4G22390	23.054 down	2.08	2.68	7.20
254326_at		AT4G22610	14.652 up	8.97	10.02	6.15
254276_at		AT4G22820	5.049 up	11.58	11.74	9.40
254299_at	NYE1	AT4G22920	3.250 down	7.70	7.79	9.49
254287_at		AT4G22960	3.844 up	9.81	9.91	7.96
254263_at		AT4G23493	3.982 up	7.93	8.05	6.06
254264_at		AT4G23510	2.704 up	6.35	6.51	5.07
254211_at	SGT1A	AT4G23570	2.587 up	8.30	8.31	6.94
254225_at		AT4G23670	5.110 up	13.66	13.56	11.21
254125_at	TAR2	AT4G24670	2.775 down	7.28	7.12	8.59
254090_at		AT4G25010	7.660 down	6.04	5.27	8.21
254098_at	FSD1	AT4G25100	4.027 down	11.48	11.07	13.08
254093_at	AtMC2	AT4G25110	2.616 down	7.29	6.50	7.89
254107_at		AT4G25220	1.546 down	10.48	10.45	11.08
254020_at	BETA-OHASE 1	AT4G25700	1.645 up	8.92	9.46	8.74
254078_at		AT4G25710	1.360 down	9.52	9.58	10.02
254042_at	XTR6	AT4G25810	3.326 down	7.21	6.47	8.20
253958_at		AT4G26400	1.335 up	9.99	10.13	9.71
253982_at	ATCBL3	AT4G26570	2.282 up	11.44	11.64	10.45
253928_at	PPX1	AT4G26720	1.481 up	10.64	10.57	10.01
253935_at		AT4G26870	3.246 up	11.30	11.20	9.50
253937_at	MAPKKK16	AT4G26890	2.161 up	6.26	6.57	5.46
253950_at		AT4G26910	16.599 up	13.02	13.00	8.95
253914_at		AT4G27400	12.349 down	7.38	7.55	11.18
253872_at	RD26	AT4G27410	2.610 up	11.29	11.75	10.36
253835_at	BGLU9	AT4G27820	5.573 down	5.41	5.24	7.72
253841_at	BGLU10	AT4G27830	34.323 up	9.36	9.30	4.20
253839_at		AT4G27890	9.043 up	9.42	9.46	6.28
253794_at		AT4G28720	2.455 up	9.14	9.40	8.10
253755_at	RPT2a	AT4G29040	1.557 down	12.22	12.30	12.94
253751_at		AT4G29070	1.508 up	9.53	9.81	9.21
253735_at	SNF7.1	AT4G29160	1.275 up	10.19	10.37	10.02
253690_at		AT4G29550	6.480 down	7.08	7.18	9.87
253660_at		AT4G30140	5.731 down	8.21	7.84	10.36
253628_at	XTH18	AT4G30280	9.460 up	11.78	11.96	8.71
253453_at		AT4G31860	1.868 down	10.58	10.61	11.52
253502_at	CYP82C4	AT4G31940	100.899 down	3.59	0.25	6.90
253413_at	ZIP9	AT4G33020	1.681 down	8.02	7.79	8.54
253373_at		AT4G33150	2.578 up	10.26	10.61	9.24
253367_at		AT4G33180	1.617 down	8.52	8.46	9.15
253372_at	PME44	AT4G33220	5.455 down	6.19	5.79	8.24
253344_at		AT4G33550	6.599 up	11.84	12.53	9.80
253312_s_at		AT4G33850	48.213 down	1.78	0.89	6.48
253270_at	CYCD3;1	AT4G34160	2.104 up	8.96	9.05	7.97
253287_at		AT4G34270	2.093 up	11.15	11.10	10.03
253206_at	SQS1	AT4G34640	2.738 up	12.29	12.22	10.77
253253_at		AT4G34750	1.265 up	10.43	10.44	10.10
253195_at		AT4G35420	4.558 down	8.26	8.34	10.52
253104_at		AT4G36010	2.942 down	8.36	8.16	9.72
246270_at		AT4G36500	2.044 up	11.19	11.30	10.27
246272_at	MES9	AT4G37150	2.897 down	9.53	9.25	10.78
253052_at	CYP81H1	AT4G37310	4.708 down	8.58	8.48	10.72

			Ler over Cvi	log2	Expression	Level
**Probe Set ID**	**Gene Symbol**	**AGI**	**Fold change**	**HGI2.1**	***Ler***	**Cvi**
253097_at	CYP81D5	AT4G37320	3.206 down	8.64	8.49	10.17
253053_at		AT4G37470	1.659 up	11.57	11.78	11.05
252979_at		AT4G38225	1.543 down	8.17	8.16	8.78
252997_at	ATEXLA2	AT4G38400	1.844 down	9.22	9.03	9.92
252870_at	AKN2	AT4G39940	1.793 up	11.85	11.62	10.78
251109_at	FER1	AT5G01600	3.611 up	12.88	12.76	10.91
251033_at		AT5G01960	1.348 down	9.80	9.93	10.36
251031_at	OHP	AT5G02120	4.081 up	10.00	10.01	7.98
251023_at		AT5G02170	4.562 up	9.40	10.25	8.06
251008_at		AT5G02710	2.326 down	6.05	5.84	7.05
250931_at		AT5G03200	20.069 down	5.06	4.80	9.13
250920_at		AT5G03390	7.612 down	3.95	3.69	6.62
250952_at	ATIREG2	AT5G03570	2.101 up	9.57	9.84	8.77
250832_at	NAS1	AT5G04950	2.700 down	11.75	11.49	12.92
250704_at		AT5G06265	8.322 up	10.16	10.39	7.33
250689_at		AT5G06610	1.767 up	9.76	9.85	9.03
250611_at	GA20OX3	AT5G07200	4.832 down	7.07	6.97	9.24
250607_at	IPK2a	AT5G07370	1.553 up	10.58	10.85	10.22
250557_at	CPuORF16	AT5G07840	2.591 up	9.27	9.38	8.01
250558_at	TT7	AT5G07990	2.454 up	9.84	9.52	8.23
250547_at		AT5G08100	1.638 up	10.99	10.92	10.20
246055_at	AtAGAL1	AT5G08380	2.895 up	9.82	10.18	8.65
250528_at		AT5G08600	5.886 down	5.43	5.11	7.66
245884_at		AT5G09300	3.346 up	10.46	10.31	8.57
245885_at	EXL4	AT5G09440	2.324 up	12.52	12.71	11.49
250500_at		AT5G09530	1.789 down	13.88	13.66	14.50
250515_at		AT5G09570	3.314 down	8.23	7.33	9.06
250494_at	HAM2	AT5G09740	2.753 up	9.08	9.11	7.65
250474_at	ANNAT7	AT5G10230	2.809 up	10.43	10.59	9.10
250450_at	ATMYB92	AT5G10280	2.066 up	9.43	9.35	8.30
245905_at		AT5G11090	1.574 up	11.64	12.02	11.37
245204_at		AT5G12270	17.643 down	3.13	3.48	7.63
250242_at		AT5G13620	4.533 down	2.97	2.86	5.04
246595_at	FDH	AT5G14780	2.997 up	10.96	11.24	9.66
250152_at		AT5G15120	13.132 down	3.52	2.42	6.14
250136_at	DRM1	AT5G15380	4.256 down	6.26	6.09	8.18
246515_at		AT5G15710	1.833 down	8.68	8.74	9.62
250113_at	FRL1	AT5G16320	1.590 down	8.09	8.23	8.90
250100_at	GLN1;4	AT5G16570	3.216 up	8.98	8.79	7.10
250105_at	RAD4	AT5G16630	2.180 down	7.94	7.98	9.11
246455_at		AT5G16790	4.086 up	4.79	5.03	3.00
250084_at	SDG40	AT5G17240	3.273 down	6.84	6.82	8.53
250091_at		AT5G17340	3.600 up	8.96	8.93	7.08
246427_at	ER-ANT1	AT5G17400	1.437 up	9.73	9.85	9.33
250046_at		AT5G17720	10.641 up	6.44	6.88	3.46
249984_at		AT5G18400	1.830 down	10.47	10.23	11.10
249983_at		AT5G18470	6.449 down	8.11	8.24	10.93
249957_at		AT5G18900	1.763 up	11.69	11.72	10.91
249922_at	AILP1	AT5G19140	2.423 down	10.56	10.54	11.82
249918_at		AT5G19240	5.305 up	12.70	12.31	9.90
249920_at		AT5G19260	1.763 down	9.10	8.62	9.44
245951_at	ASP2	AT5G19550	1.187 down	12.83	12.61	12.86
245945_at	ROPGEF10	AT5G19560	1.757 down	7.10	7.37	8.19
246141_at		AT5G19920	1.369 down	8.28	8.07	8.53

			L*er* over Cvi	log_2	Expression	Level
Probe Set ID	Gene Symbol	AGI	Fold change	HGI2.1	L*er*	Cvi
246098_at		AT5G20400	1.761 up	11.42	11.61	10.79
245993_at		AT5G20700	1.510 down	12.63	12.59	13.18
246181_at		AT5G20860	14.370 down	7.00	6.94	10.79
249900_at	emb1211	AT5G22640	1.118 down	9.38	9.39	9.55
249850_at		AT5G23240	2.474 down	4.64	5.30	6.60
249817_at		AT5G23820	14.180 up	13.46	13.82	9.99
249767_at		AT5G24090	3.365 up	11.18	11.36	9.61
249773_at	SQP2	AT5G24140	71.069 down	5.65	5.96	12.12
249737_at		AT5G24480	6.257 down	6.43	6.11	8.76
249738_at		AT5G24510	5.193 down	5.18	4.70	7.07
246957_at		AT5G24670	2.774 down	9.08	9.19	10.66
246944_at		AT5G25450	1.984 up	8.76	8.50	7.52
246901_at		AT5G25630	2.801 down	7.20	7.49	8.98
246909_at		AT5G25770	1.672 up	11.68	11.98	11.24
246831_at	MSS1	AT5G26340	3.142 down	10.85	10.48	12.13
246822_at		AT5G26960	2.603 down	7.81	7.63	9.01
246765_at		AT5G27330	5.105 down	8.05	7.59	9.94
255917_at		AT5G28560	19.946 down	4.80	5.28	9.60
255860_at	AtGUS3	AT5G34940	3.215 up	11.12	11.06	9.38
246651_at		AT5G35170	2.792 up	7.89	8.46	6.98
246610_at		AT5G35400	1.721 down	7.83	7.74	8.52
249675_at		AT5G35940	18.209 up	12.01	12.43	8.24
249690_at		AT5G36210	5.921 up	10.93	10.93	8.36
246617_at		AT5G36270	5.146 up	9.41	9.36	6.99
249636_at	BGLU42	AT5G36890	2.888 down	9.16	9.36	10.90
249576_at		AT5G37690	2.343 down	11.29	11.09	12.32
249584_s_at	NIP4;1	AT5G37810	2.580 down	7.39	6.92	8.28
249595_at		AT5G37930	3.490 up	10.08	10.24	8.43
249601_at		AT5G37980	5.266 up	8.40	8.14	5.74
249567_at		AT5G38020	6.560 down	9.23	9.14	11.86
249466_at	RPL5B	AT5G39740	2.397 up	13.84	13.69	12.43
249437_at		AT5G39990	4.272 down	9.51	9.53	11.62
249388_at		AT5G40090	1.715 up	8.28	8.21	7.43
249357_at		AT5G40490	2.142 down	9.22	9.20	10.30
249331_at	RPL27	AT5G40950	4.337 down	8.17	8.36	10.48
249255_at	CHX18	AT5G41610	2.213 up	7.37	7.51	6.36
249279_at		AT5G41920	1.436 up	6.70	7.17	6.64
249165_at	ATIPK1	AT5G42810	2.900 down	9.31	9.16	10.70
249136_at		AT5G43180	4.346 up	10.73	10.84	8.72
249097_at		AT5G43520	26.931 up	8.31	8.05	3.30
249101_at		AT5G43580	32.022 up	10.52	10.86	5.86
249096_at		AT5G43910	5.725 up	9.47	9.57	7.05
249032_at		AT5G44910	2.000 up	9.41	9.38	8.38
249000_at		AT5G44980	3.399 down	3.29	4.56	6.32
248978_at	AtPP2-A8	AT5G45070	36.577 down	5.80	5.46	10.65
248994_at	RPS4	AT5G45250	106.227 up	7.20	7.62	0.89
248996_at	RRS1	AT5G45260	16.505 up	7.49	7.97	3.93
248944_at		AT5G45500	160.073 down	2.98	3.05	10.37
248945_at		AT5G45510	10.640 down	9.21	9.31	12.73
248819_at		AT5G47050	3.853 down	8.64	8.74	10.69
248796_at		AT5G47180	1.619 up	10.90	11.02	10.32
248793_at	atnudt8	AT5G47240	3.410 up	8.96	9.60	7.83
248790_at	TIP2;3	AT5G47450	5.124 up	11.63	12.54	10.18
248758_at		AT5G47620	1.363 down	11.52	11.45	11.89

Probe Set ID	Gene Symbol	AGI	L*er* over Cvi<br>Fold change	log$_2$<br>HGI2.1	Expression<br>L*er*	Level<br>Cvi
248772_at		AT5G47800	38.917 down	3.30	1.56	6.84
248725_at		AT5G47980	5.645 up	13.73	13.67	11.17
248736_at		AT5G48110	13.792 up	10.77	11.42	7.64
248693_at		AT5G48330	1.923 down	8.62	8.48	9.43
248695_at		AT5G48350	40.940 up	7.64	7.62	2.26
248673_at		AT5G48780	15.503 up	7.72	8.16	4.20
248624_at		AT5G48790	6.504 up	8.34	8.35	5.65
248681_at		AT5G48900	4.841 up	11.64	11.71	9.43
248647_at	SUS2	AT5G49190	2.120 down	5.72	6.03	7.11
248523_s_at	SAE1B	AT5G50580	1.507 up	9.21	9.26	8.67
248407_at		AT5G51500	2.017 down	11.13	10.98	11.99
248336_at		AT5G52420	2.505 up	8.66	8.77	7.44
248366_at		AT5G52510	4.757 up	10.86	11.03	8.77
248327_at		AT5G52750	2.718 down	6.31	5.15	6.59
248245_at		AT5G53190	2.551 up	7.95	8.77	7.42
248267_at	GLT1	AT5G53460	1.699 down	12.92	12.73	13.49
248187_at		AT5G53940	5.182 up	9.70	9.51	7.14
248183_at		AT5G54040	32.134 down	5.39	4.96	9.97
248132_at	SGP1	AT5G54840	5.324 up	10.63	10.82	8.41
248140_at		AT5G54980	4.166 up	10.04	10.47	8.41
248060_at		AT5G55560	2.100 down	7.00	7.27	8.34
248003_at		AT5G56220	2.809 up	10.08	9.92	8.43
247988_at		AT5G56910	3.834 down	6.51	6.08	8.02
247879_at		AT5G57770	1.702 down	8.26	8.16	8.93
247797_at		AT5G58780	19.056 down	3.19	2.14	6.39
247742_at		AT5G58980	3.494 down	7.28	7.44	9.25
247706_at		AT5G59480	4.038 down	8.83	8.99	11.01
247555_at	ECT3	AT5G61020	1.600 down	12.66	12.53	13.21
247577_at		AT5G61290	5.696 down	4.69	5.66	8.17
247519_at	ANAC100	AT5G61430	3.602 down	7.64	7.51	9.36
247497_at	PPAN	AT5G61770	1.829 down	9.82	9.70	10.57
247477_at		AT5G62340	4.075 up	13.25	13.50	11.48
247390_at		AT5G63520	2.111 down	8.17	7.88	8.96
247284_at	OPT4	AT5G64410	4.185 up	9.58	10.07	8.00
247235_at		AT5G64580	2.463 down	7.98	7.67	8.97
247250_at	FAS2	AT5G64630	1.626 up	8.89	8.92	8.22
247213_at	PROPEP1	AT5G64900	3.308 up	8.29	8.51	6.78
247215_at	PROPEP3	AT5G64905	3.672 up	9.08	8.99	7.12
247151_at	bHLH093	AT5G65640	2.207 down	10.13	10.02	11.16
247114_at		AT5G65910	3.891 down	8.33	8.33	10.29
247124_at		AT5G66060	2.150 down	8.11	8.37	9.48
247127_at		AT5G66100	1.416 down	9.56	9.48	9.98
247092_at	ATFOLT1	AT5G66380	1.812 down	9.28	9.19	10.04
247073_at	PSBO1	AT5G66570	2.027 up	10.40	10.75	9.73
259796_at			5.877 down	5.18	5.46	8.01
252754_at			18.212 up	9.07	8.94	4.75
251458_at			31.850 down	2.30	2.16	7.15
245222_at			308.092 down	1.08	1.44	9.71
245032_at			5.485 up	6.92	7.90	5.45
245050_at			4.420 down	7.18	7.49	9.63
245023_at			3.434 down	6.80	7.82	9.60
245021_at			3.285 up	8.83	9.27	7.55
244935_at			7.628 up	11.56	11.77	8.84
244936_at			5.016 up	8.82	9.53	7.20
